# Modic changes—Their associations with low back pain and activity limitation: A systematic literature review and meta-analysis

**DOI:** 10.1371/journal.pone.0200677

**Published:** 2018-08-01

**Authors:** Christofer Herlin, Per Kjaer, Ansgar Espeland, Jan Sture Skouen, Charlotte Leboeuf-Yde, Jaro Karppinen, Jaakko Niinimäki, Joan Solgaard Sørensen, Kjersti Storheim, Tue Secher Jensen

**Affiliations:** 1 Kiropraktor Kliniken Laurin, Malmö, Sweden; 2 Department of Sports Science and Clinical Biomechanics, University of Southern Denmark, Odense, Denmark; 3 Department of Radiology, Haukeland University Hospital, Bergen, Norway; 4 Department of Clinical Medicine, University of Bergen, Bergen, Norway; 5 Department of Physical Medicine and Rehabilitation, Haukeland University Hospital, Bergen, Norway; 6 Department of Global Public Health and Primary Care, University of Bergen, Norway; 7 The Spine Center of Southern Denmark, Hospital Lillebælt, Middelfart, Denmark; 8 Institute of Regional Health Research, University of Southern Denmark, Odense, Denmark; 9 Center for Life Course Health Research, University of Oulu, Oulu, Finland; 10 Medical Research Center Oulu, Oulu University Hospital and University of Oulu, Oulu, Finland; 11 Finnish Institute of Occupational Health, Oulu, Finland; 12 Research Unit of Medical Imaging, Physics and Technology, University of Oulu, Oulu, Finland; 13 University of Southern Denmark, Odense, Denmark; 14 Communication and Research Unit for Musculoskeletal Disorders (FORMI), Oslo University Hospital, Ullevål, Norway; 15 Faculty of Medicine, University of Oslo, Norway; 16 Nordic Institute of Chiropractic and Clinical Biomechanics, Odense, Denmark; Universita degli Studi di Palermo, ITALY

## Abstract

**Background:**

Previous systematic reviews have reported positive associations between Modic changes (MCs) and low back pain (LBP), but due to their narrow scope and new primary studies, there is a need for a comprehensive systematic review. Our objectives were to investigate if MCs are associated with non-specific LBP and/or activity limitation and if such associations are modified by other factors.

**Methods:**

A protocol for this review was registered at PROSPERO prior to commencing the work (PROSPERO record: CRD42015017350). The MEDLINE, CINAHL and EMBASE databases were searched for relevant studies from first record to June 15th 2016. Prospective or retrospective cross-sectional cohort studies and case-control studies including people of all ages from general, working and clinical study populations were eligible for inclusion. Risk of bias assessment and data extraction for associations and potential modifiers were completed independently by pairs of reviewers. Meta-analysis was performed for homogeneous studies and presented as odds ratios (OR) with 95% CI.

**Results:**

In all, 5210 citations were identified and 31 studies were included. One study had low risk of bias. Fifteen studies (48%) reported statistically significant positive associations between MCs and LBP and one study found a statistically significant negative association. Meta-analysis performed for studies using concordant pain with provocative discography as the clinical outcome resulted in an OR of 4.01 (1.52–10.61). One of seven studies reported a statistically significant positive association between MCs and activity limitation. Lumbar disc level and disc degeneration were found to modify the association between MCs and LBP.

**Conclusions:**

The results from this comprehensive systematic review indicate that the associations between MCs and LBP-related outcomes are inconsistent. The high risk of bias and the heterogeneity in terms of study samples, clinical outcomes and prevalence estimates of MCs and LBP may explain these findings. It is likely that new studies with low risk of bias will affect the direction and strength of these associations.

## 1. Introduction

**Fig 1:**
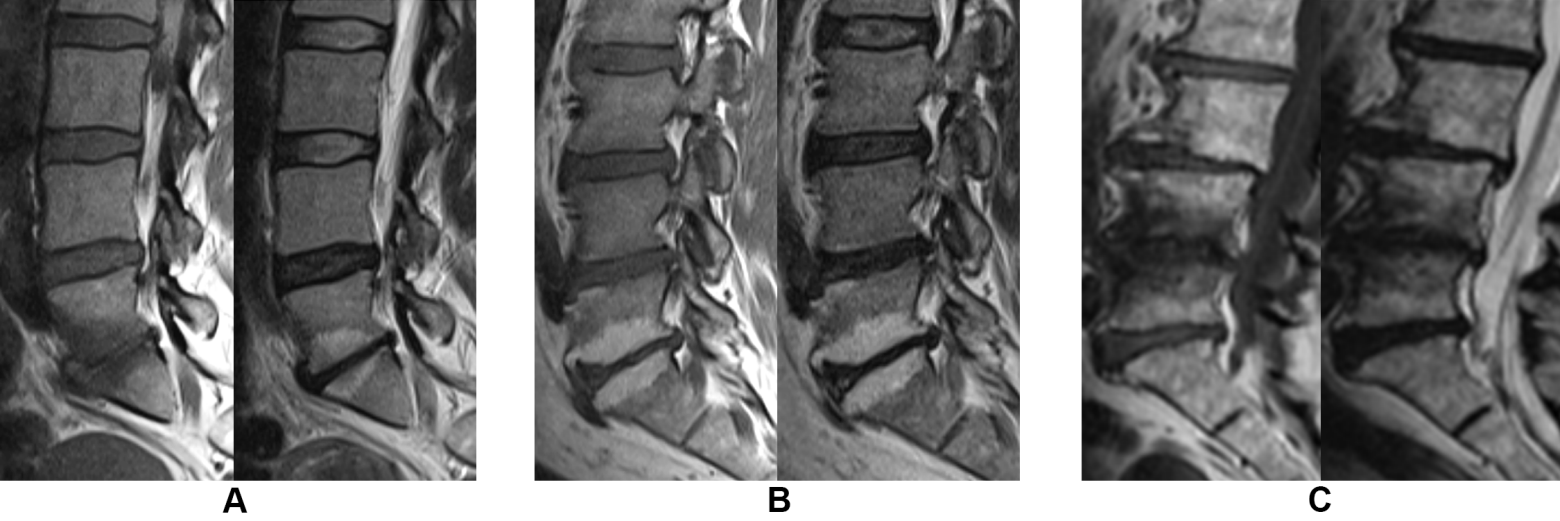
**Modic changes on T1- (left) and T2-weighted (right) images from a 1.5 Tesla MRI scanner.** (A) Modic changes type 1 at level L5-S1 (B) Modic changes type 2 at level L5-S1 (C) Modic changes type 3 at level L4-L5.

Low back pain (LBP) is a very common condition, with a one-year period prevalence of approximately 50% in people from the Nordic populations [1]. It is also the leading cause of years lived with disability worldwide [2]. Identifying the etiology of LBP is challenging and consequently patients are often labelled as having non-specific LBP [3]. In order to better understand non-specific LBP, groups of researchers have begun to test the hypothesis that LBP is not one condition, but more likely the predominant symptom of a number of, as yet, unidentified subgroups [4–6]. In the search for nociceptive contributors to pain, magnetic resonance imaging (MRI) is increasingly used. Two recent systematic reviews have identified a number of lumbar MRI findings that are associated with LBP [7, 8], and Modic Changes (MCs), i.e. endplate related signal changes in the vertebrae, have been proposed to constitute a diagnostic subgroup amongst patients with non-specific LBP [9].

de Roos et al. [10] were the first to describe endplate-related signal changes in the lumbar spine in 1987 and these were further examined by Modic et al., who classified them into three types [11, 12]: Modic changes type 1 (MCs1), Modic changes type 2 (MCs2), and Modic changes type 3 (MCs3), based on their appearance on T1-weighted and T2-weighted MRI. MCs1, seen as high signal on T2-weighted and low signal on T1-weighted magnetic resonance images, are considered to be the earliest stage of MCs, but also the most biologically active, and hypothesized to represent an inflammatory reaction in the bone marrow (edema type) [13] (Fig 1A). MCs2, seen as high signal on T1 images and isointense or slightly hyperintense signal on T2 images, represent a fat infiltration of the bone marrow (Fig 1B). MCs3, seen as low signal on both T1 and T2 images, represent a sclerotic change of the bone marrow (Fig 1C). Histological samples of MCs1 and MCs2 have shown fissuring of the vertebral endplate and trabecular bone along with vascularized fibrous tissue (MCs1) and yellow fat (MCs2) [11, 14]. The reported type and prevalence of MCs may depend on the field strength of the MRI scanner. In one study [15], more MCs overall and more MCs2 but fewer MCs1 were diagnosed in a 1.5 Tesla versus a 0.3 Tesla scanner. The appearance of MCs also depends on the MRI sequences used; e.g. on T2-weighted fat-suppression sequences, fat in MCs2 –but not edema in MCs1 –appears with a suppressed and lower signal.

The association between MCs and non-specific LBP has been investigated in three systematic reviews: Jensen et al. 2008 [16], Zhang et al. 2008 [17] and Brinjikji et al. 2015 [7]. All three reviews found an association between MCs and LBP, but Brinjikji et al. [7] only found it for MCs1. Although the title of the study by Zhang et al. [17] indicates that this is a systematic review, it has the form of a narrative review. The study by Brinjiki et al. [7] had strict inclusion criteria (studies in English only, with both symptomatic and asymptomatic participants between 15 and 50 years of age). Since 2008, when the last comprehensive review was published, many new studies have emerged and there is a need for an updated review. Furthermore, none of the previous reviews addressed the association between MCs and activity limitation, and none of them evaluated potential factors that could modify the associations (e.g. age, sex, MRI parameters, other degenerative findings such as disc degeneration, herniations and facet joint arthrosis). Albert et al. in 2013 found that MCs may have a bacterial etiology [18] and can be treated with antibiotics [19]. These findings created headlines worldwide and much debate among clinicians and researchers [20–26] because of a potential risk of bias, conflicting results in studies investigating a bacterial etiology [18, 27–29], and the prospect of treating a large group of LBP patients with long-term high-dose antibiotics.

The heterogeneous results for associations between specific types of MCs and LBP in previous systematic reviews, our lack of knowledge about the association between MCs and activity limitation, controversies about MCs guiding antibiotic treatment and emerging new studies call for a comprehensive and updated systematic literature review to improve our understanding of the clinical relevance of MCs.

Our objectives were to investigate 1) if the presence of MCs (including types and size) in the lumbar spine region is associated with non-specific LBP and/or activity limitation, and 2) if such associations are modified by other factors.

## 2. Methods

### 2.1 Design

A systematic, critical literature review with meta-analysis was performed.

A research protocol was developed in advance and registered in the PROSPERO: International prospective register of systematic reviews (http://www.crd.york.ac.uk/PROSPERO/display_record.asp?ID=CRD42015017350).

### 2.2 Criteria for considering studies for this review

#### 2.2.1 Types of studies

Prospective or retrospective cross-sectional cohort studies and case-control studies were included.

We chose to exclude studies with fewer than 26 individuals. This cut-off was chosen to minimize the risk of having cells in the 2x2 tables that included zero.

#### 2.2.2 Participants

People of all ages from general, working and clinical study populations were included.

The following exclusion criteria were used:

- Studies including participants diagnosed with specific LBP such as: spondylitis, discitis or spondylodiscitis, spondyloarthropathies (e.g. ankylosing spondylitis), fracture (including isthmic spondylolisthesis), spinal cord infarction, malignancy, hematological conditions and juvenile/idiopathic scoliosis.
- Studies including participants treated with radiotherapy in the lumbar region.
- Studies including participants treated with spinal surgery (although pre-intervention data were eligible for inclusion).

#### 2.2.3 MRI findings and definitions (index test)

We defined MCs as signal changes seen on MRI in the vertebral bone marrow, extending from the endplate. This definition included signal changes regardless of etiology and excluded signal changes only present in the bone marrow away from the endplate.

We chose to include only studies evaluating MCs in the whole lumbar spine, disc levels L1-L2 to L5-S1 (except for studies using provocative discography), based on a previous report that the association between MCs and LBP is dependent upon disc level [30]. We included studies using provocative discography, since this procedure intends to localize LBP to a specific disc level, allowing study of the association between MCs and LBP at that level (rather than between and LBP and MCs).

#### 2.2.4 Target condition

Non-specific LBP of all durations was included.

#### 2.2.5 Outcomes (reference standards)

The following outcomes were measured:

1. Presence and/or intensity of LBP measured by experimental tests (e.g. provocative discography or algometry) or patient-reported outcomes.
2. Presence and/or level of activity limitation, measured by the Oswestry Disability Index (ODI), the Roland Morris Disability Questionnaire (RMDQ) or similar tools.

### 2.3 Search methods for identification of studies

#### 2.3.1 Electronic searches

A systematic search of the literature was performed using a search strategy developed in collaboration with a research librarian. The three terms “lumbar spine”, “MRI” and “Modic changes” and their relevant synonyms were used as search terms, either as free text or as Medical Subject Headings.

The MEDLINE, CINAHL and EMBASE databases were searched for relevant studies from first record to June 15^th^ 2016. No restrictions were used.

The full electronic search strategy can be found in the S1 Appendix.

#### 2.3.2 Searching other resources

Reference lists of all included studies were examined and all authors were asked to review the list of included studies for omissions.

### 2.4 Data collection and analysis

#### 2.4.1 Selection of studies

Two reviewers (CH, TSJ) independently screened the titles and abstracts to exclude clearly irrelevant papers. For each potentially eligible study, the full article was retrieved and independently assessed for inclusion (CH, TSJ). Any discrepancies were resolved by consensus. Where multiple publications used data from the same study sample, we chose the article with the most complete data related to the associations between MCs and LBP and/or activity limitation.

In cases where association data were not presented in a format that we could use for data extraction, we contacted the authors to request additional data, as recommended by the Cochrane Handbook [31].

We assessed the eligibility of non-English papers using Google Translate and, when this was impossible due to incomplete Optical Character Recognition obtained from scans of paper copies, with the help of a native speaker of the language in question.

#### 2.4.2 Data extraction and management

Data extraction and risk of bias assessment were completed by independent reviewers (CH, PK, AE, JStS, CLY, JK, JN, JSoS, KS, TSJ), allocated in pairs (except for non-English papers, where a single assessor was used), using spreadsheets (S2 Appendix, Tables 1–3). All reviewers were pre-trained through pilot-testing of the process. Inconsistencies were resolved by consensus or, if needed, by including a third reviewer (CH or TSJ).

**Table 1:** Study characteristics.

Study	Study sample	Participants (% male)	Mean age (range and/or SD)	MRI field strength and sequences	MC types and prevalences	Outcome measures and LBP prevalence (for non-clinical studies)
**Clinical non-discography studies**						
Kleinstuck 2006* [42]	Patients with chronic nsLBP, representing a subgroup from an RCT on active therapy for LBP	53 (51%)	44 (SD 11)	1.5 T. T2w	MC any: 62%	2 weeks average LBP intensity (0–10), 2 weeks worst LBP intensity (0–10), RMQ
Peterson 2014 [48]	Patients with MRI-confirmed lumbar disc herniations	346 (50%)	59.7 (20–87, SD 14.5)	1.5–3.0 T. T1w, T2w	MC any: 57%	NRS (0–10)
Schistad 2014 [49]	Patients with lumbar radicular pain due to disc herniation, recruited from two hospitals in Norway	243 (53%)	41.3 (SD 10.5)	1.5–3.0 T. T1w, T2w, FLAIR	MC1: 12.3%, MC2/3: 63.4%	VAS back pain (0–10), ODI (0–100)
Bianchi 2015 [50]	Patients recieving lumbar facet injections in a hospital setting	226 (39%)	61.6 (23–88, SD 13.3)	1.5 T. T1w, T2w	MC any: 62.4%, MC1: 36.7%, MC2: 25.7%	NRS (0–10)
Jensen 2015 [38]	Patients with LBP with or without radiculopathy	141 (47%)	41.6 (18–60, SD 10.6)	0.7 T. T1w, T2w	MC any: 60%, MC1: 18%, MC2: 42%	Back pain score (0–30)
Annen 2016 [51]	Patients with MRI-confirmed lumbar disc herniations	72 (76%)	41.9 (23–70, SD 11.4)	Not reported	MC1: 22%, MC2: 33%	NRS (0–10), ODI (0–100)
Nakamae 2016 [47]	Patients with lumbar degenerative scoliosis.	120 (25%)	LBP: 75.0 (SD 5.3) / No LBP: 76.6 (SD 5.1)	1.5 T. Fatsat. & post-gad Tw	MC1: 69%	LBP (>6 mnths, >50/100 VAS) vs. leg pain alone
**TOTAL**		**1201**				
**Discography studies**						
Braithwaite 1998 [52]	Patients referred for investigation of ‘discogenic’ LBP, with or without associated leg pain, as a precursor to spinal fusion.	58 (53%)	42 (21–63)	0.5–1.5 T. T1w, T2w	MC any: 24.2%	Concordant pain on provocative discography
Ito 1998 [53]	Patients with chronic LBP.	39 (44%)	37 (21–57)	1.5 T, T1w, T2w	MC any: 8.9%	Concordant pain on provocative discography
Weishaupt 2001 [54]	Patients with chronic LBP presumed to be of discogenic origin.	50 (54%)	42.4 (28–50)	1.0 T. T1w, T2w	MC any: 22.4%, MC1: 13.8%, MC2: 8.6%^£^	Concordant pain on provocative discography
Kokkonen 2002 [55]	Patients with chronic LBP admitted to Oulu University Hospital, Finland.	36 (61%)	40 (20–58)	Not reported	MC any: 37.9%, MC1: 16.5%, MC2: 19.4%	Concordant pain on provocative discography
Lim 2005 [56]	Patients with chronic LBP.	47 (43%)	43 (25–54)	1.5 T. T1w, T2w	MC any: 14%^£^	Concordant pain on provocative discography
O'Neill 2008 [57]	Patients in a spinal pain speciality center.	143 (64%)	42.6 (21–71)	Unknown. T1w, T2w	MC any: 8%, MC1: 3.7%, MC2: 4.3%^£^	Concordant pain on provocative discography
Kang 2009 [58]	Patients with severe chronic LBP.	62 (?)	46 (17–68)	1.5 T. T1w, T2w	MC any: 12.9%^£^	Concordant pain on provocative discography
Thompson 2009 [59]	Patients who where candidates for surgery or minimally invasive procedures.	736 (?)	43 (22–78)	Unknown. T1w, T2w	MC1: 6.3%, MC2: 5.1%, MC3: 0.9%^£^	Concordant pain on provocative discography
Chen 2011 [60]	Patients who underwent MRI of the lumbar spine and subsequent provocation discography as part of a clinical evaluation of LBP.	93 (69%)	40.1 (30–56)	1.5 T. T1w, T2w	MC any: 22.6%^£^	Concordant pain on provocative discography
**TOTAL**		**1264**				
**Non-clinical studies**						
Jarvik 2001 [36]	Persons from hospital departments (not related to LBP) without LBP more than mildly bothersome in the last 4 months	148 (89%)	54 (36–71)	1.5 T. T1w, T2w	MC any: 26.4%	Previous history of LBP (>5 times): 15%
Kjaer 2005a (adult) [61]	Persons in a cohort of 40-year-olds from Funen, Denmark	412 (48%)	40 (40–41)	0.2 T. T1w, T2w	MC any: 22.3%	LBP last month: 42%, LBP last year: 69%, Seeking care: 28%
Kjaer 2005b (children)* [41]	Children from a cohort in which all 13-year-olds living in Odense, Denmark were invited to participate	439 (47%)	13.1 (12–14)	0.2 T. T2w	MC1: 0.5%	LBP last month: 22%, Seeking care: 8%
Kuisma 2007 [30]	White males (159 train engineers, 69 office workers) in Finland	228 (100%)	47 (36–56)	1.5 T. T1w, T2w, FLAIR	MC any: 56%, MC1: 15%, MC2: 32%	Pain episodes, VAS (1 wk), VAS (1 month)
Takatalo 2012 [62]	Participants of the Northern Finland Birth Cohort (NFBC) 1986.	554 (42%)	21.2 (20–23)	1.5 T. T1w, T2w	MC any: 0.9%	Lifetime LBP: 73%, 6-month LBP: 39%, Consultation for LBP: 7%, Used medication for LBP: 40%
Koyama 2013 [63]	Japanese college gymnasts.	104 (67%)	19.7 (SD 1.0)	0.3 T. T1w, T2w	MC any: 1.9%	OCU-test (>1 point considered LBP): 49%
Mok 2016 [64]	Volunteers from a population-based cohort	2449 (?)	40.4 (SD 10.9)	Several. T2w	MC any: 5.8%	Historical LBP: 80%
Määttä 2015 [65]	Volunteers from the TwinsUK register	823 (4%)	54.0 (32–70, SD 8)	1.0 T. T2w	MC any: 32.2%	Disabling LBP >1 month during lifetime: 22.4%
Teraguchi 2015 [66]	Persons from The Wakayama Spine Study (a population-based study on spinal degenerative disease)	975 (33%)	66.4 (21–97, SD 13.5)	1.5 T. T2w	MC any: 47.1%	LBP most days last month + now: 40.3%
Määttä 2016 [67]	Volunteers from a population-based cohort	1142 (37%)	52.9 (SD 6.5)	3.0 T. T1w, T2w	MC1: 7%, MC2: 17.6%	Prolonged severe LBP: 23.6%, ODI (0–100)
**TOTAL**		**7274**				
**Case-control studies**						
Rannou 2007 [68]	Highly selected patients in three groups (MC1: 12, MC2: 12, no MC: 12)	36 (66%)	52 (SD 14)	Not reported	MC1: 33%, MC2: 33% (case-control)	LBP VAS (0–100), Quebeck Disability Score
Acar Sivas 2009 [69]	Cases: Chronic LBP patients with or without sciatica. Controls: Asymptomatic healthy individuals.	75 (20%)	Cases: 26 (22–30, SD 2.9) Controls: 25.5 (25–30, SD 3.5)	1.5 T. T1w, T2w	MC any: 3.17% (cases), 4.16% (controls)	LBP >3 months and/or sciatalgia
Hancock 2012 [70]	Cases: Acute or subacute LBP patients (with or without leg pain) Controls: Persons matched for age, sex and previous LBP history	60 (53%)	Cases: 36.8 (SD 7.4) Controls: 36.6 (SD 7.4)	1.5 T. T1w, T2w	MC any: 22%	Moderate pain (SF-36 q 7) < 6 weeks duration
Kovacs 2012 [71]	Subjects (240 cases and 64 controls) recruited from six hospitals	304 (36%)	Cases: 43 (38–47) Controls: 45 (41–47)	1.5 T. T1w, T2w	MC any: 80.4% (cases), 87.5% (controls), 81.9% (total)	LBP >90 days, RMQ
Sheng-Yun 2014 [72]	Cases: LBP patients presenting to a hospital. Controls: Asymptomatic patients	2024 (56%)	45 (SD 13)	1.5 T. T1w, T2w	MC any: 19.6% (cases) MC any: 10.5% (controls)	LBP (not further specified)
**TOTAL**		**2499**				

*: Additional data from author.

£: MCs prevalence only from discs where discography was performed.

**Table 2:** Risk of bias assessment.

	Study sample					Index test							Reference standard								Timing and data analysis			
													LBP				Activity limitation							
	Q1	Q2	Q3	Q4	Q5	Q6	Q7	Q8	Q9	Q10	Q11	Q12	Q13	Q14	Q15	Q16	Q17	Q18	Q19	Q20	Q21	Q22	Q23	Q24
**Clinical non-discography studies**																								
Kleinstuck 2006 [42]	U	Y	U	Y	**U**	Y	N	Y		Y	Y	**U**	Y	Y	Y	**Y**	Y	Y	Y	**Y**	N	U	Y	**U**
Peterson 2014 [48]	Y	Y	Y	Y	**Y**	N	Y	Y		N	Y	**Y**	Y	Y	U	**Y**					Y	N	Y	**N**
Schistad 2014 [49]	U	Y	Y	Y	**Y**	N	Y	Y	Y	Y	Y	**Y**	Y	Y	U	**Y**	Y	Y	U	**Y**	U	U	N	**U**
Bianchi 2015 [50]	Y	Y	Y	U	**Y**	N	U	Y			Y	**U**	Y	Y	U	**U**					Y	N	Y	**Y**
Jensen 2015 [38]	U	Y	U	Y	**U**	N	Y	Y			N	**N**	Y	Y	Y	**Y**					U	N	U	**U**
Annen 2016 [51]	Y	Y	U	Y	**Y**	U	Y	Y	Y		U	**U**	Y	Y	U	**Y**	Y	Y	U	**Y**	U	U	Y	**U**
Nakamae 2016 [47]	U	Y	N	U	**N**	N	N	U	U		Y	**U**	Y	Y	U	**Y**					U	U	U	**Y**
**Discography studies**																								
Braithwaite 1998 [52]	Y	Y	U	Y	**U**	N	Y	Y	U		N	**U**	Y	U	N	**N**					U	U	Y	**U**
Ito 1998 [53]	U	Y	U	Y	**U**	Y	Y	Y	Y		N	**N**	Y	Y	U	**U**					U	U	Y	**U**
Weishaupt 2001 [54]	Y	Y	Y	N	**Y**	Y	Y	Y	Y	Y	N	**U**	Y	U	U	**U**					Y	N	Y	**Y**
Kokkonen 2002 [55]	N	Y	U	U	**U**	U	Y	U	Y	U	Y	**U**	Y	U	U	**U**					U	U	U	**Y**
Lim 2005 [56]	N	Y	U	N	**N**	N	Y	Y		N	N	**U**	Y	U	N	**U**					U	N	Y	**Y**
O'Neill 2008 [57]	Y	Y	U	N	**U**	N	Y	Y			N	**N**	Y	Y	N	**N**					U	U	N	**U**
Kang 2009 [58]	Y	Y	Y	N	**U**	Y	Y	Y	Y		N	**U**	Y	Y	Y	**Y**					U	U	Y	**Y**
Thompson 2009 [59]	U	Y	U	Y	**U**	U	U	Y			N	**N**	Y	Y	U	**Y**					U	N	U	**Y**
Chen 2011 [60]	Y	Y	U	N	**U**	Y	Y	Y	U		N	**U**	Y	U	Y	**U**					U	U	U	**Y**
**Non-clinical studies**																								
Jarvik 2001 [36]	N	Y	U	U	**N**	Y	Y	N	U		U	**N**	Y	Y	Y	**Y**					U	U	Y	**U**
Kjaer 2005a (adult) [61]	Y	Y	Y	Y	**Y**	Y	Y	Y			Y	**Y**	Y	Y	U	**U**					N	U	Y	**N**
Kjaer 2005b (child) [41]	N	Y	Y	Y	**Y**	Y	Y	Y			N	**U**	Y	Y	Y	**Y**					N	Y	Y	**N**
Kuisma 2007 [30]	U	Y	U	U	**U**	Y	Y	Y	Y	Y	Y	**U**	Y	U	Y	**Y**					U	U	U	**U**
Takatalo 2012 [62]	N	Y	Y	Y	**Y**	Y	Y	Y	Y		N	**N**	Y	Y	U	**U**					U	U	Y	**U**
Koyama 2013 [63]	N	Y	U	Y	**Y**	Y	N	Y	Y	U	N	**U**					Y	Y	U	**Y**	U	U	Y	**U**
Määttä 2015 [65]	N	Y	U	Y	**U**	Y	N	Y			Y	**Y**	Y	Y	Y	**Y**					U	U	Y	**U**
Teraguchi 2015 [66]	N	Y	Y	Y	**Y**	Y	N	U			Y	**U**	Y	Y	U	**U**					N	Y	Y	**N**
Mok 2016 [64]	N	Y	U	U	**U**	N	U	Y	Y	Y	N	**U**	Y	Y	U	**U**	Y	Y	U	**U**	U	U	U	**Y**
Määttä 2016 [67]	N	Y	Y	Y	**U**	N	U	Y		Y	N	**N**	N	Y	U	**Y**	Y	Y	U	**Y**	N	Y	N	**Y**
**Case-control studies**																								
Rannou 2007 [68]	N	N	N	N	**N**	U	Y	Y	Y		N	**U**	Y	Y	Y	**Y**	Y	Y	Y	**Y**	U	U	N	**Y**
Acar Sivas 2009 [69]	U	N	U	U	**U**	Y	Y	Y			N	**N**	Y	U	Y	**U**					U	U	U	**U**
Hancock 2012 [70]	Y	N	Y	Y	**U**	Y	Y	Y	Y		Y	**Y**	Y	Y	Y	**Y**					U	Y	Y	**N**
Kovacs 2012 [71]	N	N	Y	Y	**Y**	N	Y	Y	Y	U	U	**U**	Y	Y	Y	**Y**					N	Y	Y	**N**
Sheng-Yun 2014 [72]	U	N	U	Y	**Y**	Y	Y	U	U	U	Y	**N**	U	U	U	**U**					U	U	U	**U**

Y: Yes;N: No; U: Unclear; Grey field: Not applicable; Green field: Low risk of bias;Yellow field: Unclear;Red field: High risk of bias; Q1-24: Risk of bias questions (S2 Appendix).

**Table 3:** The association between Modic changes and LBP.

MCs type(s)	Study	Outcome measure	Unadjusted estimates	
			Dichotomous outcomes	Continous outcomes •
			Odds ratios (95% CI)	Mean diff. (95% CI)
**Clinical non-discography studies**				
MCs, any type	Kleinstuck 2006* [42]	2 weeks average LBP intensity (0–10)		-0.1 (-1.58–1.38)
	Kleinstuck 2006* [42]	2 weeks worst LBP intensity (0–10)		-0.3 (-1.56–0.96)
	Peterson 2014 [48]	Baseline mean NRS (0–10)		0.01 (-0.47–0.49)
	Jensen 2015* [38]	Back pain score (0–30)		-1.8 (-4.04–0.44)
	Annen 2016 [51]	Baseline mean Backpain NRS (0–10)		-0.7 (-1.91–0.51)
MCs1	Schistad 2014 [49]	Back pain VAS (0–10)		-0.8 (-2.3–0.43)
	Bianchi 2015 [50]	Baseline mean NRS (0–10)		NS
	Jensen 2015* [38]	Back pain score (0–30) MC1 vs no-MC1		2.4 (-.53–5.33)
	Nakamae 2016 [47]	LBP (>6 mnths, >50/100 VAS) vs. leg pain alone	**51.67 (11.43–233.51)**	
MCs2	Bianchi 2015 [50]	Baseline mean NRS (0–10)		0.3 (-0.40–1.01)
	Jensen 2015* [38]	Back pain score (0–30) MC2 vs no-MC2		**-3.2 (-5.39–-1.01)**
MCs2 or 3	Schistad 2014 [49]	Back pain VAS (0–10)		-0.5 (-1.33–0.33)
**Discography studies**				
MCs, any type	Braithwaite 1998 [52]	Concordant pain on provocative discography	**9.13 (2.06–40.56)**	
	Ito 1998 [53]	Concordant pain on provocative discography	**5.14 (1.25–21.09)**	
	Weishaupt 2001 [54]	Concordant pain on provocative discography	**19.93 (5.50–72.31)**	
	Kokkonen 2002 [55]	Concordant pain on provocative discography	1.19 (0.52–2.73)	
	Lim 2005 [56]	Concordant pain on provocative discography	0.46 (0.12–1.77)	
	O'Neill 2008 [57]	Concordant pain on provocative discography	**8.69 (3.03–24.96)**	
	Kang 2009 [58]	Concordant pain on provocative discography	1.09 (0.4–2.95)	
	Chen 2011 [60]	Concordant pain on provocative discography	**14.91 (6.41–34.67)**	
MCs1	Braithwaite 1998 [52]	Concordant pain on provocative discography	9.58 (0.52–176.75)^£^	
	Weishaupt 2001 [54]	Concordant pain on provocative discography	**13.59 (2.92–63.28)**	
	Kokkonen 2002 [55]	Concordant pain on provocative discography	1.34 (0.48–4.00)	
	O'Neill 2008 [57]	Concordant pain on provocative discography	**7.90 (1.79–34.97)**	
	Thompson 2009 [59]	Concordant pain on provocative discography	**9.32 (6.17–14.09)**	
MCs2	Braithwaite 1998 [52]	Concordant pain on provocative discography	**6.96 (1.54–31.50)**	
	Weishaupt 2001 [54]	Concordant pain on provocative discography	**15.46 (1.89–126.67)**	
	Kokkonen 2002 [55]	Concordant pain on provocative discography	1.03 (0.36–2.95)	
	O'Neill 2008 [57]	Concordant pain on provocative discography	**9.48 (2.17–41.37)**	
	Thompson 2009 [59]	Concordant pain on provocative discography	0.90 (0.62–1.31)	
MCs3	Braithwaite 1998 [52]	Concordant pain on provocative discography	6.09 (0.31–120.35)^£^^$^	
	Thompson 2009 [59]	Concordant pain on provocative discography	**2.51 (1.05–5.97)**	
MCs, any type (moderate/severe)	Weishaupt 2001 [54]	Concordant pain on provocative discography	**83.10 (4.85–1424.05)**^£^	
MCs1 (moderate/severe)	Weishaupt 2001 [54]	Concordant pain on provocative discography	**42.01 (2.41–733.09)**^£^	
MCs2 (moderate/severe)	Weishaupt 2001 [54]	Concordant pain on provocative discography	**24.76 (1.38–444.85)**^£^	
**Non-clinical studies**	** **		** **	** **
MCs, any type	Jarvik 2001 [36]	Previous history of LBP	1.06 (0.31–3.15)	
	Kjaer 2005a (adult) [61]	LBP during last month	**1.86 (1.16–2.97)**	
	Kjaer 2005a (adult) [61]	LBP during last year	**4.24 (2.17–8.29)**	
	Kjaer 2005a (adult) [61]	LBP seeking care	**1.87 (1.15–3.06)**	
	Kuisma 2007 [30]	Pain episodes		**5.08 (1.11–9.05)**
	Kuisma 2007 [30]	VAS (1 week)		**.95 (.38–1.52)**
	Kuisma 2007 [30]	VAS (3 months)		**.92 (.37–1.47)**
	Takatalo 2012 [62]	Always/recent vs. Minor/no pain (Latent Cluster Analysis)	9.13 (0.94–88.59)	
	Mok 2016 [64]	Historical LBP (continuous localized pain for 2 weeks or more)	**2.17 (1.26–3.74)**	
	Määttä 2015 [65]	Disabling LBP >1 month	**2.71 (1.90–3.86)**	
	Teraguchi 2015 [66]	LBP most days last month + now (Endplate Signal Change (ESC) & Degenerative Disc)	1.06 (.78–1.43)	
	Teraguchi 2015 [66]	LBP most days last month + now (ESC and Schmorls Node (SN))	.87 (.58–1.32)	
	Teraguchi 2015 [66]	LBP most days last month + now (ESC, SN and Degenerative Disc (DD))	**1.77 (1.26–2.47)**	
	Määttä 2016 [67]	Prolonged severe LBP	**1.65 (1.16–2.37)**	
MCs1	Kjaer 2005b (children)* [41]	LBP during last month	.69 (0.03–14.48)^£^	
	Kjaer 2005b (children)* [41]	LBP seeking care	12.24 (0.75–200.20)	
	Kuisma 2007 [30]	Pain episodes		**4.82 (.26–9.38)**
	Kuisma 2007 [30]	VAS (1 week)		**.80 (.14–1.46)**
	Kuisma 2007 [30]	VAS (3 months)		**.78 (.14–1.42)**
	Määttä 2016 [67]	Prolonged severe LBP	**2.06 (1.12–3.79)**	
MCs2	Kuisma 2007 [30]	Pain episodes		1.63 (-2.65–5.90)
	Kuisma 2007 [30]	VAS (1 week)		.402 (-.22–1.02)
	Kuisma 2007 [30]	VAS (3 months)		.378 (-.22 - .98)
	Määttä 2016 [67]	Prolonged severe LBP	**1.53 (1.02–2.29)**	
MCs, any type, extensive	Kuisma 2007 [30]	Pain episodes		1.43 (-2.71–5.57)
	Kuisma 2007 [30]	VAS (1 week)		**.87 (.28–1.45)**
	Kuisma 2007 [30]	VAS (3 months)		**.78 (.21–1.35)**
	Määttä 2016 [67]	Prolonged severe LBP	**1.83 (1.14–2.94)**	** **
MCs, any type, minimal	Kuisma 2007 [30]	Pain episodes		**7.75 (1.37–14.13)**
	Kuisma 2007 [30]	VAS (1 week)		**.71 (.01–1.40)**
	Kuisma 2007 [30]	VAS (3 months)		**.75 (.06–1.43)**
**Case-control studies**	** **	** **	** **	** **
MCs, any type	Acar Sivas 2009 [69]	LBP >3 months and/or sciatalgia	0.88 (0.08–10.23)	** **
	Hancock 2012 (Assessor A) [70]	LBP < 6 weeks	**6.00 (1.17–30.73)**	
	Hancock 2012 (Assessor B) [70]	LBP < 6 weeks	**10.71 (2.15–53.35)**	
	Kovacs 2012 [71]	VAS (0–10)	0.43 (0.14–1.29)	
	Sheng-Yun 2014 [72]	LBP (not further specified)	**2.07 (1.41–3.04)**	
MCs1	Rannou 2007 [68]	Pain VAS (100 mm)		13 (-4.25–30.25)
MCs2	Rannou 2007 [68]	Pain VAS (100 mm)		12 (-2.94–26.95)

NS: non-significant estimate

•: (MC+)-(MC-) calculated from raw data where available, using t-test.

*: Additional data from author.

£: Added 0.5 to all cells in 2x2 table.

$: All Type 3 MCs were combined with either Type 1 or 2.

Data regarding sample source, number of subjects, age, MRI parameters, observers, MCs (including types and size), clinical outcomes, and strength of associations between LBP and/or activity limitation and MCs were extracted from the papers (S2 Appendix).

Data regarding possible modifiers or confounders of the associations between MCs and clinical outcomes were extracted and classified according to how the covariates were analysed: a) by matching on the covariate(s), b) by restricting participant selection so that all groups had the same covariate value, or c) by adjustment for covariates in the statistical analysis. For the purpose of this review, only analyses investigating single covariates were included. The reason for this was that adjustment by groups of covariates (e.g. age, sex, etc.) might change the estimate of the associations, but would not provide information as to which of the group covariates or combinations of covariates were modifying the associations. Because of the exploratory nature of this part of the review, we chose not to make a list of pre-defined candidate variables [32].

#### 2.4.3 Risk of bias assessment

We based our risk of bias assessment on the QUADAS 2 tool [33]. This tool is used to evaluate the following four key domains: *study sample*, *index test*, *reference standard(s)*, *timing and data analysis* based on signaling questions and questions regarding applicability. For each domain, studies were classified as having ‘low risk of bias’, ‘high risk of bias’ or ‘unclear’ based on a number of signaling questions. Studies were classified as having an ‘overall low risk of bias’ if all four domains were scored as ‘low risk of bias’ [33]. We added additional signaling questions pertaining to items we found particularly important for the subject of this review. After pilot-testing, we modified some of the risk of bias questions and response options, making them more intuitive to answer (S2 Appendix). The questions regarding applicability were not used in this study. The result of our risk of bias assessment was not used as an inclusion criterion.

#### 2.4.4 Statistical analysis and data synthesis

Raw data for 2x2 tables or group differences were extracted where possible to calculate odds ratios (OR) with 95% confidence intervals (CI) for dichotomous outcomes or to perform t-tests for continuous outcomes. In cases where results were presented in the form of ORs or mean differences, without raw data, we present them as stated in the article, using data from the crude analysis, i.e. unadjusted. Data supplied by authors on request were treated in the same manner. ORs and 95% CIs were calculated for 2x2 tables. For tables containing 0 in one of the cells, we added 0.5 to all cells [34]. Differences in means between groups were analysed using a t-test. Statistical analyses were performed using STATA (version 12.1, StataCorp, College Station, Texas, USA).

Associations between subtypes and sizes of MCs and outcomes were determined with reference to participants with no MCs.

A statistically significant association was defined as CIs not including 1.0 for dichotomous outcomes and a p-value below 0.05 for continuous outcomes. Studies were classified as having an association (‘positive study’ or ‘negative study’) if the association reported for one or more outcomes was statistically significant. If a single study reported both a statistically significant positive association and a statistically significant negative association, this would be classified as a contradictory association (‘contradictory study’).

Results were pooled where it was deemed possible and appropriate (e.g. homogeneous in terms of study sample or outcome), and associations reported as ORs and 95% CIs. Due to the heterogeneity in terms of the prevalence estimates of MCs and study sampling, a random effect model was used. I^2^ statistics were used to quantify inconsistency across studies. *Comprehensive Meta-Analysis* (version 3, Biostat, Englewood, USA) was used for meta-analysis.

A single covariate was recorded to modify the associations between MCs and LBP/activity limitation if a) the estimates for unadjusted and adjusted/stratified analyses differed or b) an interaction term with MCs and the possible modifier was statistically significant, p<0.05.

Pre-determined sensitivity analyses were performed for publication bias and overall risk of bias. The classification of associations (positive, negative or contradictory) was then tested against mean age, year of publication, number of participants, and overall risk of bias using Fischer’s exact test.

## 3. Results

### 3.1 Selection of studies

In total, 5210 citations were identified, yielding 3834 records after removal of duplicates. After reviewing titles and abstracts, 3377 records were excluded, resulting in a total of 457 papers eligible for full text assessment. Another 420 studies were excluded in the full text assessment (S3 Appendix), resulting in 37 potential candidates. Two additional studies were found through manual search [35] and by a person from the research team [36] respectively, resulting in 39 potentially acceptable studies.

**Fig 2:**
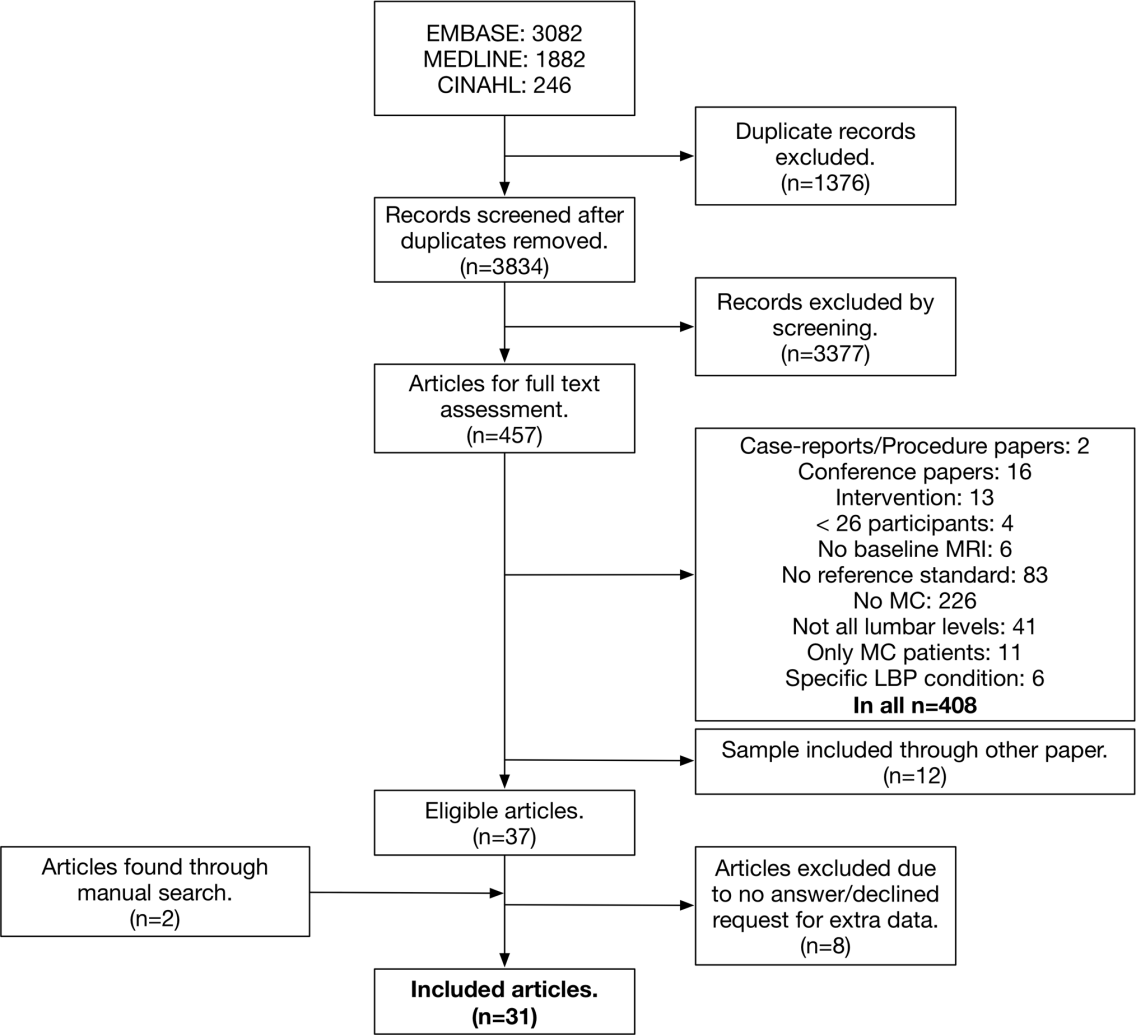
Flow diagram of study selection.

We requested additional data needed for analysis from the authors [35, 37–46] for 11 of the 39 studies, but received these data from only three [38, 41, 42]. We thus ended up including 31 studies (Fig 2).

Almost all inconsistencies of data extraction and Risk of Bias assessment were solved by consensus among the pairs of assessors. A third reviewer was involved in reaching consensus for five data points.

### 3.2 Study characteristics

Sixteen studies reported on clinical populations (Table 1). These consisted of patients with or without leg pain, most of them being classified as having chronic LBP or referred for back surgery of some kind. A notable exception was the study by Nakamae et al. [47], where the patients had lumbar degenerative scoliosis, and mixed LBP and leg pain were used as an exclusion criterion. Ten studies reported data from non-clinical populations, i.e. population-based cohorts, volunteers or working populations and five studies reported data from case-control studies. In relation to outcomes, 21 studies reported on self-reported LBP (13 studies on the presence of LBP and eight studies on the intensity of LBP). Nine studies reported on pain at provocative discography (no study reported on other experimental tests, e.g. algometry). Seven studies reported on activity limitation.

The number of participants ranged from 36 to 2449, with a median of 200, with the proportion of women ranging from 0% to 96%. Mean age of the study samples ranged from 13 to 76 (Table 1).

The majority of studies used MRI scanners with field strengths of 1.0–1.5 T. Four studies used a field strength below 1.0 T. Six studies used several scanners for their assessments. Five studies used only T2-weighted MRI sequences, making differentiation between different types of MCs impossible, whereas the remaining studies used both T1-weighted and T2-weighted sequences. One study used T2-weighted fat-suppression or post-contrast T1-weighted sequences. Three studies did not report on any MRI parameters at all (Table 1).

#### 3.2.1 Prevalence of MCs

The prevalence of MCs, with all types taken into account, ranged from 3% to 80% in clinical samples (including cases from case-control studies), on a per individual basis, not including studies using provocative discography (Table 1). For clinical studies using provocative discography, the range was 1% to 38%, reported per level assessed by discography. In non-clinical samples (including controls from case-control studies) the prevalence ranged from 0.5% to 88% on a per individual basis. Kovacs et al. reported the highest prevalence of MCs among non-LBP participants (88%), a number higher than even the highest prevalence (62%) found in the clinical studies (Table 1).

#### 3.2.2 Prevalence of LBP

The prevalence estimates of LBP in non-clinical and case-control studies were measured with a variety of criteria, eg. ‘LPB last month’ and ‘lifetime LBP’, and thus varied considerably (Table 1).

### 3.3 Risk of bias assessment

The results from the risk of bias assessment can be seen in Table 2. Only one study [48] was classified as having overall low risk of bias, i.e. with low risk of bias in all four key domains. Five studies had three domains with low risk of bias, three studies had two domains with low risk of bias, fourteen studies had one domain with low risk of bias and eight studies were classified as having no domains with low risk of bias.

### 3.4 Association between MCs and LBP

#### 3.4.1 Association with LBP

Across all included papers, 30 of 31 studies reported on the association between MCs (regardless of type) and LBP. Of these 30, 15 found statistically significant positive associations with ORs ranging from 1.53 (95% CI 1.02–2.29) to 83.10 (95% CI 4.85–1424.05), while only one found a statistically significant negative association with a mean difference between patients with and without MCs2 of -3.2 (-5.39 –-1.01) on a ‘back pain score’ ranging from 0 to 30 [38]. The remaining 14 studies reported statistically non-significant findings, of which eight [38, 41, 42, 49, 51, 56, 69, 71] reported negative (but non-significant) estimates on at least one of their outcome measures. No studies reported contradictory statistically significant associations (Table 3).

Across all included articles, 13 studies reported on the association between MCs1 and LBP. Six of these found statistically significant positive associations. Five reported ORs ranging from 2.06 (95% CI 1.12–3.79) to 51.67 (95% CI 11.43–233.51) for dichotomous outcomes [47, 54, 57, 59, 67] whereas one study reported statistically significant positive associations using continuous outcomes [30] (Table 3). The remaining seven studies reported statistically non-significant findings for associations regarding MC1 [38, 41, 49, 50, 52, 55, 68].

Ten studies reported on the association between MCs2 and LBP. Four reported statistically significant positive associations with ORs ranging from 1.53 (95% CI 1.02–2.29) to 15.46 (95% CI 1.89–126.67) [52, 54, 57, 67]. One study reported a statistically significant negative association with a mean difference between patients with and without MCs2 of -3.2 (-5.39 –-1.01) [38]. The remaining five studies reported statistically non-significant findings for associations regarding MCs2 [30, 50, 55, 59, 68].

The wide range of ORs and the broad and overlapping 95% CIs indicate that there is no significant difference between MCs1 and MCs2 in regard to their associations with LBP (Table 3).

Two studies reported on the association between MCs3 and LBP and one of these found a positive association with OR 2.51 (95% CI 1.05–5.97) [59] (Table 3). The remaining study reported a statistically non-significant finding for association regarding MCs3 [52].

#### 3.4.2 Associations between different sizes of MCs and LBP

Three studies [30, 54, 67] reported statistically significant positive associations between extensive MCs and LBP; two reported ORs of 1.83 (95% CI 1.14–2.94) and 83.10 (95% CI 4.85–1424.05) and one reported on continuous outcomes, see Table 3.

However, the estimates for extensive MCs were not different from those for MCs of any type, regardless of size.

#### 3.4.3 Pooled results

Due to the heterogeneity of the observational (non-discography) study samples (differences in outcome measures and in study sampling, see Table 1) no meta-analysis was performed for these studies. However, meta-analysis was performed for the nine studies using concordant pain with provocative discography as the outcome measure. Separate analyses were made for *MCs any*, *MCs1* and *MCs2* resulting in ORs (95% CI) of 4.01 (1.52–10.61), 6.14 (2.47–15.27), and 3.15 (1.00–9.93), respectively, indicating that there was no significant difference in the associations with LBP between the two types of MCs. Substantial heterogeneity was identified for all three analyses with I^2^-values of 84, 64 and 81, respectively (Table 4).

**Table 4:** Discography studies–pooled results.

MCs type(s)	Study	OR (95% CI)
MCs, any type	Braithwaite 1998 [52]	**9.13 (2.06–40.56)**
	Ito 1998 [53]	**5.14 (1.25–21.09)**
	Weishaupt 2001 [54]	**19.93 (5.50–72.31)**
	Kokkonen 2002 [55]	1.19 (0.52–2.73)
	Lim 2005 [56]	0.46 (0.12–1.77)
	O'Neill 2008 [57]	**8.69 (3.03–24.96)**
	Kang 2009 [58]	1.09 (0.4–2.95)
	Chen 2011 [60]	**14.91 (6.41–34.67)**
	**TOTAL (Random)**	**4.01 (1.52–10.61)**
MCs1	Braithwaite 1998 [52]	9.58 (0.52–176.75)^£^
	Weishaupt 2001 [54]	13.59 (2.92–63.28)*
	Kokkonen 2002 [55]	1.34 (0.48–4.00)
	O'Neill 2008 [57]	**7.90 (1.79–34.97)**
	Thompson 2009 [59]	9.32 (6.17–14.09)*
	**TOTAL (Random)**	**6.14 (2.47–15.27)**
MCs2	Braithwaite 1998 [52]	**6.96 (1.54–31.50)**
	Weishaupt 2001 [54]	15.46 (1.89–126.67)**
	Kokkonen 2002 [55]	1.03 (0.36–2.95)
	O'Neill 2008 [57]	**9.48 (2.17–41.37)**
	Thompson 2009 [59]	0.90 (0.62–1.31)**
	**TOTAL (Random)**	**3.15 (1.00–9.93)**

The association between Modic changes and LBP for studies using concordant pain on provocative discography as outcome.¨

*: MCs1 vs. non-MCs1

**: MCs2 vs. non-MCs2

£: Added 0.5 to all cells in 2x2 table.

#### 3.4.4 LBP intensity in patients with and without MCs

None of the six clinical studies investigating the difference in LBP intensity between patients with and without MCs found a significant difference between the two groups [38, 42, 48–51].

### 3.5 Association between MCs and activity limitation

#### 3.5.1 Association with activity limitation

One of seven studies (three clinical, three non-clinical and one case-control) reporting on activity limitation outcomes found a statistically significant association between MCs and activity limitation. Määttä et al. reported an association between activity limitation (ODI>15%) and both any MCs and MCs2, OR 1.47 (95% CI 1.04–2.10) and 1.56 (95% CI 1.06–2.31), respectively, but not for MCs1 [67]. (Table 5)

**Table 5:** The association between Modic changes and activity limitation.

MCs type(s)	Study	Outcome measure	OR (95% CI)	Mean difference •
**Clinical studies**				
MCs, any type	Kleinstuck 2006 [42]	RMQ (0–24)		1.1 (-1.75–3.95) p = 0.45
	Annen 2016 [51]	ODI (0–100)		3.32 (-0.73–7.37) p = 0.11
MCs1	Schistad 2014 [49]	ODI (0–100)		2.8 (-4.65–10.25) p = 0.46
MCs2 or 3	Schistad 2014 [49]	ODI (0–100)		5.4 (-0.33–11.13) p = 0.07
**Non-clinical studies**				
MCs, any type	Koyama 2013 [63]	Osaka City University Questionnaire (OCU-test)	0.20 (0.01–4.27)^£^	
	Mok 2016 [64]	ODI (0–100)	NS*	
	Mok 2016 [64]	RMQ (0–24)	NS*	
	Määttä 2016 [67]	ODI (0–100)	**1.47 (1.04–2.10)**	
MCs1	Määttä 2016 [67]	ODI (0–100)	1.23 (0.67–2.24)	
MCs2	Määttä 2016 [67]	ODI (0–100)	**1.56 (1.06–2.31)**	
**Case-control studies**				
MCs1	Rannou 2007 [68]	Quebeck Disability Score (0–100)		1 (-10.65–12.65) p = 0.87
MCs2	Rannou 2007 [68]	Quebeck Disability Score (0–100)		4 (-6.68–14.68) p = 0.48

•: (MCs+)-(MCs-) calculated from raw data where available, using t-test.

*: No raw data supplied.

£: Added 0.5 to all cells in 2x2 table.

#### 3.5.2 Level of activity limitation in patients with and without MCs

None of the four clinical studies investigating the difference in activity limitation levels between patients with and without MCs found a significant difference between the two groups [42, 49, 51, 68].

### 3.6 Is the association between MCs and outcomes modified by other factors?

In relation to identifying single modifiers of the association between MCs and clinical outcomes, five studies reported stratified analyses, four with stratification on disc levels [30, 64–66] and one on sex [61]. Of the four studies that stratified by disc level, three identified statistically significant associations only for some levels. Two studies found the associations to be stronger at the two lower levels [30, 64], while one found statistically significant positive associations at L1-L2, L4-L5 and L5-S1 [66]. In the one study reporting stratified analyses on sex, MCs were associated with LBP only for men when using the outcome measure ‘LBP month’, only for women when using the outcome measure ‘Seeking care’ and for both sexes when using the outcome measure ‘LBP year’ [61].

In two studies, the authors reported analyses where disc degeneration was included as a modifying factor. In one study, disc degeneration was included as an interaction term [71] and in one study as a single covariate in two separate multivariable analyses [67]. In both studies, disc degeneration was reported to reduce the estimates of association between MCs and LBP by 10–28%.

With regard to possible modifiers of the association between MCs and activity limitation, two studies investigated this [64, 67]. In the study by Mok et al., the authors reported that disc level did not affect the association between MCs and activity limitation (as measured by the Oswestry Disability Index and Roland Morris Disability Questionnaire). In the study by Määttä et al., disc degeneration reduced the association between MCs and the Oswestry Disability Index.

### 3.7 Sensitivity analysis

Of the 30 studies investigating LBP (one study did not), 15 studies reported statistically significant positive associations with MCs. The results of the sensitivity analysis are reported in Table 6.

**Table 6:** Sensitivity analysis.

	Significant positive association		
	Yes (n = 15)	No (n = 15)	P-value
**Mean age of study population, median (IQR)**	43 (40–53)	43 (41–52)	p<0.90
**Number of participants, n (%)**			
<100	5 (33%)	7 (47%)	
101–500	4 (27%)	7 (47%)	p<0.14
>501	6 (40%)	1 (6%)	
**Year of publication, median (IQR)**			
<2005	3 (20%)	2 (13%)	
2006–2010	4 (27%)	6 (40%)	p<0.79
>2010	8 (53%)	7 (47%)	
Overall risk of bias	15 (100%)	14 (93%)	p<1.00
***Post Hoc analysis***			
Positive *insignificant* estimate of association, n (%)*	4 (25%)^$^	9 (60%)^£^	
Negative *insignificant* estimate of association, n (%)*	2 (12%)^$^	8 (53%)^£^	
**Risk of bias (RoB) by domains, n (%)**			
RoB in relation to study population sampling	9 (60%)	9 (60%)	p<1.00
RoB in relation to index test	10 (67%)	12 (80%)	p<0.68
RoB in relation to reference standard	12 (73%)	6 (40%)	p<0.14
RoB in relation to timing and analysis	10 (67%)	11 (73%)	p<1.00
RoB for selected signaling questions, n (%)^§^			
MRI results interpreted w/o knowledge of LBP	4 (27%)	2 (20%)	p<0.65
LBP assessment interpreted w/o knowledge of MRI	9 (60%)	6 (40%)	p<0.47
Reliability study	8 (53%)	9 (60%)	p<1.00
<1 month between MRI and LBP	12 (80%)	13 (87%)	p<1.00
**LBP outcomes, n (%)**			
Provocative discography	6 (40%)	3 (20%)	p<0.43
Continuous outcome measure	0 (0%)	8 (53%)	p<0.01
Dichotomous outcome measure	15 (100%)	7 (47%)	
Diagnostic test study, i.e. discography	6 (40%)	3 (20%)	p<0.61
Cohort study	7 (47%)	9 (60%)	
Case-control study	2 (13%)	3 (20%)	
**MRI field strength, n (%)**			
Not reported	1 (7%)	2 (13%)	
<1.5 Tesla	3 (20%)	2 (13%)	p<1.00
1.5+ Tesla	7 (47%)	8 (53%)	
Several field strengths	4 (23%)	3 (20%)	

Characteristics and risk of bias in studies *with* a statistically significant positive association between Modic changes and LBP (n = 15) versus *without* a statistically significant positive association between Modic changes and LBP (n = 15). No studies reported contradictory statistically significant associations.

* Studies reporting positive or negative insignificant estimates, for at least one outcome.

^**§**^ Selection based on what were deemed most important for diagnostic test study.

^**$**^ Test performed for within-group differences (p<0.36)

^£^ Test performed for within-group differences (p<0.71)

The publication of statistically significant positive associations between MCs and LBP were not related to year of publication (p<0.79), classified as 1998–2004 (n = 5), 2005–2010 (n = 10) and 2011–2016 (n = 15), nor to the total number of participants (p<0.14), divided into <100 participants (n = 12), 100–500 participants (n = 11) and more than 500 participants (n = 7).

As only one of seven studies evaluating activity limitation was classified as having a statistically significant positive association and the remaining studies showed non-significant associations, sensitivity analysis for this outcome was not meaningful.

Only one study was classified as having ‘no overall risk of bias’ and performing a sensitivity analysis on the overall risk of bias assessment was therefore not meaningful.

#### 3.7.1 Post hoc sensitivity analysis

To further investigate the possible influence of bias and other factors in the reporting of a statistically significant association between MCs and LBP, we performed post hoc analyses of the classification of associations (statistically positive association, yes/no) for individual risk of bias domains and signaling questions, LBP outcomes, study design, and MRI field strength.

There was a statistically significant difference (p<0.01) in the distribution of studies using continuous or dichotomous outcomes (Table 6). All 15 studies that reported significant positive associations used dichotomous outcomes, e.g. ‘LBP < 6 weeks’, as compared to only half (53%) of the 15 studies that did not report significant positive association. No other statistically significant differences were identified between the two groups of studies.

## 4. Discussion

### 4.1 Main findings

In summary, the results show inconsistent associations between MCs and both LBP and activity limitation. Only half of the studies reported statistically significant positive associations between MCs and LBP. Both pooled and individual study data indicate that there is no difference in the strength of associations of MCs1 and MCs2 with LBP. Among patients with LBP, the *intensity* of LBP does not seem to differ between those with MCs and those without. Only one of seven studies found an association between MCs and activity limitation. Finally, our results indicate that disc level and disc degeneration modify the association between MCs and LBP. With respect to previous systematic reviews on this subject [7, 16, 17], these are new results and will be discussed in more detail below.

### 4.2 Discussion of findings

#### 4.2.1 Inconsistent positive association between MCs and LBP

The proportion of studies that showed a statistically significant positive association between MCs and LBP was lower in this review (50%) compared to the previous reviews, 88% [17] and 70% [16]. Amongst the studies classified as having a statistically significant positive association, almost a third also had estimates that were non-significant (with some of these being negative) [30, 52, 59, 66]. This finding, along with eight of the 15 studies reporting non-significant associations (also including negative estimates on at least one outcome measure [38, 41, 42, 49, 51, 56, 69, 71]) and one study that reported a statistically significant negative association [38], indicate that the association between MCs and LBP is more inconsistent than previously reported.

It would be reasonable to assume that the heterogeneity of study quality, samples, sex, clinical outcomes, and the prevalence estimates of both MCs and LBP could explain the conflicting results. However, in an attempt to explain the differences between studies that did and studies that did not report significant positive associations between MCs and LBP, sensitivity analyses were performed for sample size, publication year, study design, type of LBP outcome, and MRI field strength. None of these explained the differences in the directions and strengths of associations. The prevalence of MCs is, of course, dependent on the definition of MCs used by the different authors, which could help explain the large variation seen in reported prevalence of MCs. There is large variation in the interpretation of when MCs are present in the included studies, e.g. “all signal changes in the vertebral bone, extending from the endplate, regardless of size” [73, 74] vs. “tiny spots of signal intensity change in the bone marrow adjacent to the vertebral corners, were not recorded.” [30]. However, the lack of detailed reporting of definitions of MCs in the majority of studies made it impossible to analyse the impact of different phenotypes of MCs on the association with outcomes.

Although the pooled results from the discography studies revealed statistically significant positive associations for all types of MCs with estimates ranging from OR 3.2 to OR 6.1, their 95% CIs are wide, and range from 1.00–15.27. Provocative discography carries inherent risks of bias when used as a diagnostic test in the presence of MRI findings [75]. Patients subjected to discography are selected on the basis of clinical findings, including MRI. When the reference standard (LBP by provocative discography) is not blinded from the index test (MRI), there is a risk of circular reasoning that could confound the association. Furthermore, there are laboratory data showing increased intradiscal pressure at discs adjacent to the injected level in animal models, calling into question the validity of provocative discography [76, 77].

Because of the shortcomings mentioned, care must be taken when interpreting the results from the analyses of associations in this review. It is possible that future large scale high quality studies will affect the direction of the associations presented above.

#### 4.2.2 Type and size of MCs do not seem to matter

There was no significant difference in the strength of associations between MCs1 and LBP and MCs2 and LBP, either in the individual studies or according to the pooled results. Intuitively, one would believe that MCs1 would have a stronger association with pain than MCs2, due to the fact that MCs1 are supposed to occur in response to an inflammatory reaction [13], whereas MCs2 are considered a more biologically inactive entity. However, the lack of difference in strength of associations with pain could be attributed to the fact that (1) MCs1 and MCs2 can co-exist at the same disc level and/or within the same individual [78–80], and that (2) MCs2 often follow MCs1, making MCs2 a possible proxy for further degenerative changes (e.g. disc degeneration, protrusions/herniations) that are potentially painful [7].

With regard to the size of MCs, we found that the estimates for the associations between ‘extensive’ MCs and LBP are not different from those between MCs of any size and LBP. One possible explanation for this is that considering a normal stimulus response curve for pain, the plateau for pain may be reached even for small discovertebral lesions.

#### 4.2.3 No difference in LBP intensity between patients with and without MCs

The results of the six clinical studies that investigated the LBP intensity in patients with and without MCs, indicate that patients with MCs may not experience more intense pain than those without MCs and thus, they may be difficult to identify solely based on pain intensity. The lack of difference in pain intensity between patients with and without MCs may be explained by the fact that all patients with LBP are in pain and that the pain experience is influenced by a multitude of factors other than nociception [81]. Another explanation could be that MCs are only one finding among others in the degenerative chain of events, where disc degeneration, herniations and osteophyte formation each play their part [7, 82] and as such, MCs do not always stand out as the main contributor to LBP.

#### 4.2.4 No support for association between MCs and activity limitation

To the knowledge of the authors, this is the first systematic review to investigate the cross-sectional association between an MRI finding and pain-related activity limitation. Only one of the seven studies that reported on this association found a statistically significant positive association between activity limitation and MCs. In that study, by Määttä et al. [67] the crude estimates for associations between MCs and activity limitation were mainly positive. Based on the results from the current review, there is no evidence to support that MCs are cross-sectionally associated with activity limitation. However, in support of a positive association, a recent longitudinal study by Järvinen et al. investigating patients with MCs and LBP, found that change in the extent of MCs1 was positively associated with 2-year changes in the Oswestry Disability Index, both unadjusted and adjusted for age, sex and size of MCs at baseline [83].

#### 4.2.5 The association with LBP is likely modified by disc level and disc degeneration

Although only based on three [30, 64, 66] and two studies [67, 71], respectively, disc level and disc degeneration were identified as potential modifiers of the association between MCs and LBP. A possible reason for MCs at the lower disc levels being more strongly associated with LBP is that this part of the lumbar spine is subjected to increased discovertebral load [84]. Therefore, lower disc level is likely to be a proxy for other factors, e.g. increased physical load or injury to the discovertebral complex, that could lead to LBP [85–87]. However, due to the low prevalence of MCs in the upper lumbar spine, the estimates for these levels are uncertain, and therefore more research would be needed to make it possible to more closely evaluate disc level as a possible modifying factor.

The confounding of the association between MCs and LBP by disc degeneration may be explained by studies reporting that disc degeneration is an independent risk factor of LBP [7, 65].

#### 4.2.6. Overall risk of bias of included studies

There was an overall risk of bias in all included studies but one [48]. This risk was partly due to insufficient reporting and may not necessarily imply actual bias. Still, risk of bias needs to be taken into account when interpreting the current results and when performing new primary studies.

The most common problems within each of the four bias domains were: 1) Lack of randomly or consecutively selected study participants, which could introduce a risk of selection bias, 2) Lack of reliability testing, raising concerns about misclassification which would influence prevalence rates of MCs, and thus also the strengths, directions and validity of associations as these are dependent on the prevalence, 3) Lack of blinding between assessment of outcome measure and MRI results, which was mainly an issue for discography studies where patients were referred for the procedure on the basis of the results of their MRI scan, which might have introduced beliefs that could affect their reporting of pain, and 4) Failure to report on the timing of the MRI and clinical outcome assessments, with longer periods increasing the risk of change in either MRI appearance or LBP/activity limitation status.

### 4.3 Limitations and strengths

#### 4.3.1 Limitations

While our sensitivity analysis did not show that study design influenced the result, we did include case-control studies, although they are less suited for our purpose due to the fact that the groups are from different samples, thus introducing a potential bias, as described in the Cochrane Handbook [88].

By only including studies that had evaluated MCs at all lumbar levels, it is possible that we excluded high quality studies that could have informed on the association between MCs and LBP. However, that decision was made to allow us to investigate the modifying effect of disc level on the association between MCs and LBP, which was novel analysis.

#### 4.3.2 Strengths

We did a broad search without language restriction in three major databases, supplemented with a hand search and query for additional studies from experts. The search was not restricted to terms for “low back pain” and “activity limitation”/“back-related disability”, since we originally wanted to investigate the prevalence of MCs as well. This strategy reduced the risk of missing important studies. That the relatively large group of reviewers, all with a special interest in MCs, only found one additional article, likely indicates that our search strategy was comprehensive.

The results from this review are based on three times as many studies than previous reviews. The increased number of included studies helps estimate the direction and strength of the associations even though the heterogeneity of the studies made it inappropriate to perform meta-analysis on the relationships between MCs and self-reported LBP and pain-related activity limitation respectively.

We based our risk of bias on the QUADAS 2 [33], a recommended and validated tool for the task, but modified it after pilot-testing without further validation. A pilot study was performed on both the data extraction and risk of bias assessment, which familiarized the assessors with the process, and highlighted problematic areas, which were changed before initiating the study. For the extraction of data and risk of bias assessment, assessors were blinded to the assessments of their fellow co-assessors up until consensus.

Our review was performed by a large number of reviewers from different research groups, all of whom were familiar with the subject of MCs.

### 4.4 Recommendations for further research

The widely different prevalence rates reported for MCs in similar populations may indicate inconsistent phenotyping of MCs. Agreement on the characterization of MCs across studies is needed, including criteria for size and for differentiation from other signal changes (e.g. fat or edema in osteophytes, inhomogeneous bone marrow, hemangiomas abutting the endplate), or at least a concise reporting of the methods used to evaluate these findings (including all relevant MRI parameters), in order to be able to compare results between studies.

In light of the results of our risk of bias assessment, we urge researchers to improve their reporting of the methods used. In particular, we found weaknesses related to the selection of study samples, reliability testing on MRI assessments, blinding and study logistics (timing of assessments). Researchers might also assess whether other characteristics of MCs (e.g. location [67], extent [67], their signal after fat suppression [89]) may be more relevant to pain than are the type of MCs based on conventional T1- and T2-weighted MRI. To be able to further our understanding of the details of the association between MCs and LBP, we need large population-based cohort studies with low risk of bias that allow for stratified or multivariable analyses including known and suspected modifiers.

### 4.5 Clinical implications of our findings

The lack of difference in pain intensity between patients with MCs and patients without MCs, along with the sparse knowledge around other distinguishing clinical characteristics, makes identification of patients with MCs difficult, without the use of MRI.

However, this may be without clinical relevance, as our finding of a more inconsistent association between LBP and MCs than previously shown should call for caution when using ‘Modic changes’ as a diagnosis, explanation for LBP, and indication for specific treatment in patients with non-specific LBP.

### 4.6 Conclusion

The results from this systematic review show that the associations between Modic changes and both outcomes of low back pain and activity limitation are inconsistent. Heterogeneity in terms of study samples, classification of Modic changes, clinical outcomes and prevalence of Modic changes and low back pain may explain the inconsistent associations. Also, no difference in low back pain intensity or level of activity limitation was found between patients with and without Modic changes. These results question the conclusions from previously published reviews that Modic changes may constitute a specific clinically relevant subgroup among people with low back pain. Disc level and disc degeneration were identified as factors potential modifying the association between Modic changes and low back pain. New studies with low risk of bias are likely to affect the direction and strength of these associations.

## Supporting information

S1 Appendix Full search strategy. (DOCX)

S2 Appendix Data extraction and risk of bias assessment. (XLSX)

S3 Appendix Articles excluded in full text assessment. (DOCX)

S4 Appendix Protocol registered at PROSPERO. (PDF)

S5 Appendix PRISMA checklist. (DOCX)
