# Supplementary material for: Modic changes—Their associations with low back pain and activity limitation: A systematic literature review and meta-analysis
Source: PLoS One. 2018 Aug 1;13(8):e0200677. doi: 10.1371/journal.pone.0200677 (PMC6070210; doi:10.1371/journal.pone.0200677)
Supplement: S1 Appendix — (DOCX) [file pone.0200677.s001.docx]

# Search strategy for MEDLINE, EMBASE and CINAHL respectively

Search terms within groups were combined with the boolean operator OR.

## MEDLINE

| **Group** | **Search term** |
| --- | --- |
| lumbar spine | Lumbar Vertebra/ |
|  | Lumbosacral Region/ |
|  | lumb*.tw,kw. |
|  | low back.tw,kw. |
| **AND** | |
| MRI | Magnetic Resonance Imaging/ |
|  | magnetic resonance.tw,kw. |
|  | mri.tw,kw. |
|  | nmr.tw,kw. |
|  | diagnostic imaging.tw,kw. |
| **AND** | |
| Modic | Intervertebral Disc Degeneration/ |
|  | Spondylosis/ |
|  | Bone Marrow/ |
|  | spondylosis.tw,kw. |
|  | bone marrow.tw,kw. |
|  | osteochondrosis.tw,kw. |
|  | osteochondritis.tw,kw. |
|  | modic.tw,kw. |
|  | apophysis.tw,kw. |
|  | scheuermann.tw,kw. |
|  | schmorl*.tw,kw. |
|  | discovertebral.tw,kw. |
|  | (edema OR oedema).tw,kw. |
|  | ((endplate? or end plate? or end-plate?) not "motor endplate?").tw,kw. |

Database: Ovid ”MEDLINE(R) In-Process & Other Non-Indexed Citations and Ovid MEDLINE(R) 1946 to Present”

## EMBASE

| **Group** | **Search term** |
| --- | --- |
| lumbar spine | lumbar vertebra/ |
|  | lumbar spine/ |
|  | lumbosacral spine/ |
|  | lumb*.tw,kw. |
| **AND** | |
| MRI | nuclear magnetic resonance imaging/ |
|  | magnetic resonance.tw,kw. |
|  | mri.tw,kw. |
|  | nmr.tw,kw. |
|  | diagnostic imaging.tw,kw. |
| **AND** | |
| Modic | intervertebral disc degeneration/ |
|  | spondylosis/ |
|  | bone marrow/ |
|  | spondylosis.tw,kw. |
|  | bone marrow.tw,kw. |
|  | osteochondrosis.tw,kw. |
|  | osteochondritis.tw,kw. |
|  | modic.tw,kw. |
|  | apophysis.tw,kw. |
|  | scheuermann.tw,kw. |
|  | schmorl*.tw,kw. |
|  | discovertebral.tw,kw. |
|  | (edema OR oedema).tw,kw. |
|  | ((endplate? or end plate? or end-plate?) not "motor endplate?").tw,kw. |

Database: Ovid – ”**Embase**1974 to 2015 March 02”

## CINAHL

| **Group** | **Search term** |
| --- | --- |
| lumbar spine | (MH "Lumbar Vertebrae") |
|  | TX lumb* |
|  | TX low back |
| **AND** | |
| MRI | (MH "Magnetic Resonance Imaging+") |
|  | TX magnetic resonance |
|  | TX mri |
|  | TX nmr |
|  | TX diagnostic imaging |
| **AND** | |
| Modic | (MH "Spondylosis") |
|  | (MH "Bone Marrow") |
|  | TX spondylosis |
|  | TX bone marrow |
|  | TX osteochondrosis |
|  | TX osteochondritis |
|  | TX modic |
|  | TX apophysis |
|  | TX scheuermann |
|  | TX schmorl* |
|  | TX discovertebral |
|  | TX (edema OR oedema) |
|  | TX (endplate# OR end plate# OR end-plate# NOT motor end#plate#) |

Database: EBSCOhost - CINAHL
