# Supplementary material for: Modic changes—Their associations with low back pain and activity limitation: A systematic literature review and meta-analysis
Source: PLoS One. 2018 Aug 1;13(8):e0200677. doi: 10.1371/journal.pone.0200677 (PMC6070210; doi:10.1371/journal.pone.0200677)
Supplement: S3 Appendix — (DOCX) [file pone.0200677.s003.docx]

# Appendix S3 – Articles excluded in full text assessment

## Case reports

1. McAfee PC. Total disc replacement. Operative Techniques in Orthopaedics. 2003;13(3):214-21.

2. Sorensen JS, Albert HB, Manniche C. Unexpected MRI findings in patients with sciatica. [Danish]. Ugeskrift for Laeger. 2004;166(37):3207-8.

## Conference papers

1. Briot K, Paternotte S, Miceli-Richard C, Dougados M, Roux C. Use of non steroidal anti-inflammatory drugs and high body mass index prevent bone loss in patients with early inflammatory back pain: Results from the DESIR cohort. Annals of the Rheumatic Diseases. 2013;72.

2. Chiba K, Matsuyama Y. Efficacy and safety of condoliase in patients with lumbar disc herniation: A prospective randomized controlled clinical trial. European Spine Journal. 2015;1):S687-S8.

3. De Bruin F, Treyvaud M, Feydy A, Bloem J, Dougados M, Gossec L, et al. Degenerative changes of the spine on MRI in patients with inflammatory back pain from the DESIR cohort. Annals of the Rheumatic Diseases. 2014;73.

4. Djurasovic M, Crawford CH, Zook JD, Kelly RB, Glassman SD, Carreon LY. The impact of MRI findings on clinical outcomes in lumbar fusion. Spine Journal. 2010;1):73S.

5. Joe E, Lee JW, Lee GY, Cho SE, Kang HS. Prediction of cartilage endplate herniation in MRI. Neuroradiology. 2013;55:S97.

6. Kang K, Shen M, Zhao W, Lurie JD, Razi A. Retrolisthesis and lumbar disc herniation: A postoperative assessment of patient function. Spine Journal. 2011;1):104S.

7. Kobayashi S, Yonezawa T. Aseptic bone necrosis induced by percutaneous laser disc decompression (PLDD) mr imaging and clinical correlation. European Spine Journal. 2014;23:S550.

8. Samartzis D, Cheung KM. Prediction of future first-time low back pain based on baseline MRI findings. Spine Journal. 2013;1):16S-7S.

9. Senol S, Kilic G, Baspinar S, Kilic E, Ozgocmen S. Degenerative changes at the lumbar spine in patients with axial spondyloarthritis and non-specific mechanical low back pain: A magnetic resonance imaging study. Arthritis and Rheumatology Conference: American College of Rheumatology/Association of Rheumatology Health Professionals Annual Scientific Meeting, ACR/ARHP. 2015;67(no pagination).

10. Shvyreva N, Shostak N, Pravdyuk N, Radenska-Lopovok S, Peyker A. Spine osteoarthritis-clinical and morphological comparisons. Annals of the Rheumatic Diseases. 2013;72.

11. Takenaka S, Hosono N, Mukai Y, Tateishi K, Fuji T. Preoperative retrolisthesis is a risk factor of lumbar disc herniation after fenestration without discectomy. European Spine Journal. 2014;23:S486-S7.

12. Teraguchi M. Prevalence and distribution of intervertebral disc degeneration over the entire spine in a population-based cohort: The wakayama spine study. Spine Journal. 2013;1):83S.

13. Yamada K, Fujimoto Y, Nakamae T, Matsuura M. Targeted therapy of low back pain associated with de novo degenerative lumbar scoliosis in the elderly: Observation cohort study. Spine Journal. 2014;1):S127.

14. Yamada K, Fujimoto Y, Nakamae T, Matsuura M. Association of vertebral bone marrow edema with low back pain in degenerative lumbar scoliosis in the elderly: A cross-sectional observational study. Spine Journal. 2014;1):S9.

15. Yamada K, Fujimoto Y, Ujigo S, Takazawa A. Novel and extreme minimally invasive intervention for low back pain associated with degenerative lumbar scoliosis in the elderly. Spine Journal. 2011;1):127S.

16. Yin R, Wang JC, Lord EL, Cohen JR, Takahashi S. Distribution of schmorl's nodes in the lumbar spine and their relationship with lumbar disc degeneration and range of motion. Spine Journal. 2014;1):S108.

## Interventions

1. Barth M, Diepers M, Weiss C, Thome C. Two-year outcome after lumbar microdiscectomy versus microscopic sequestrectomy - Part 2: Radiographic evaluation and correlation with clinical outcome. Spine. 2008;33(3):273-9.

2. Beaudreuil J, Dieude P, Poiraudeau S, Revel M. Disabling chronic low back pain with Modic type 1 MRI signal: acute reduction in pain with intradiscal corticotherapy. Annals of Physical & Rehabilitation Medicine. 2012;55(3):139-47.

3. Cvitanic OA, Schimandle J, Casper GD, Tirman PF. Subchondral marrow changes after laser diskectomy in the lumbar spine: MR imaging findings and clinical correlation. AJR American Journal of Roentgenology. 2000;174(5):1363-9.

4. Esposito P, Pinheiro-Franco JL, Froelich S, Maitrot D. Predictive value of MRI vertebral end-plate signal changes (Modic) on outcome of surgically treated degenerative disc disease. Results of a cohort study including 60 patients. Neuro-Chirurgie. 2006;52(4):315-22.

5. Grand CM, Bank WO, Baleriaux D, Matos C, Levivier M, Brotchi J. Gadolinium enhancement of vertebral endplates following lumbar disc surgery. Neuroradiology. 1993;35(7):503-5.

6. Han C, Ma X, Ma J, Wang T, Wang P. Distribution characteristics of Modic changes of lumbar endplate and its relationship with low back pain. [Chinese]. Zhongguo xiu fu chong jian wai ke za zhi = Zhongguo xiufu chongjian waike zazhi = Chinese journal of reparative and reconstructive surgery. 2009;23(12):1409-12.

7. Kang KK, Shen MS, Zhao W, Lurie JD, Razi AE. Retrolisthesis and lumbar disc herniation: a postoperative assessment of patient function. Spine Journal: Official Journal of the North American Spine Society. 2013;13(4):367-72.

8. Kato F, Ando T, Kawakami N, Mimatsu K, Iwata H. The increased signal intensity at the vertebral body endplates after chemonucleolysis demonstrated by magnetic resonance imaging. Spine. 1993;18(15):2276-81.

9. Lei D, Rege A, Koti M, Smith FW, Wardlaw D. Painful disc lesion: Can modern biplanar magnetic resonance imaging replace discography? Journal of Spinal Disorders and Techniques. 2008;21(6):430-5.

10. Mariconda M, Galasso O, Attingenti P, Federico G, Milano C. Frequency and clinical meaning of long-term degenerative changes after lumbar discectomy visualized on imaging tests. European Spine Journal. 2010;19(1):136-43.

11. Rahme R, Moussa R, Bou-Nassif R, Maarrawi J, Rizk T, Nohra G, et al. What happens to modic changes following lumbar discectomy? Analysis of a cohort of 41 patients with a 3- to 5-year follow-up period: Clinical article. Journal of Neurosurgery: Spine. 2010;13(5):562-7.

12. Rahme R, Moussa R, Bou-Nassif R, Maarrawi J, Rizk T, Nohra G, et al. Lumbar microdiscectomy: a clinicoradiological analysis of outcome. Canadian Journal of Neurological Sciences. 2011;38(3):439-45.

13. Weiner BK, Vilendecic M, Ledic D, Eustacchio S, Varga P, Gorensek M, et al. Endplate changes following discectomy: natural history and associations between imaging and clinical data. European Spine Journal. 2015;24(11):2449-57.

## Less than 26 participants

1. Buttermann GR, Heithoff KB, Ogilvie JW, Transfeldt EE, Cohen M. Vertebral body MRI related to lumbar fusion results. European Spine Journal. 1997;6(2):115-20.

2. Kim SY, Lee IS, Kim BR, Lim JH, Lee J, Koh SE, et al. Magnetic resonance findings of acute severe lower back pain. Annals of Rehabilitation Medicine. 2012;36(1):47-54.

3. Korhonen T, Karppinen J, Paimela L, Malmivaara A, Lindgren KA, Bowman C, et al. The treatment of disc-herniation-induced sciatica with infliximab: one-year follow-up results of FIRST II, a randomized controlled trial. Spine. 2006;31(24):2759-66.

4. Ohtori S, Yamashita M, Yamauchi K, Inoue G, Koshi T, Suzuki M, et al. Low back pain after lumbar discectomy in patients showing endplate modic type 1 change. Spine. 2010;35(13):E596-600.

## No baseline MRI

1. Buttermann GR. The effect of spinal steroid injections for degenerative disc disease. Spine Journal: Official Journal of the North American Spine Society. 2004;4(5):495-505.

2. Elfering A, Semmer N, Birkhofer D, Zanetti M, Hodler J, Boos N. Young investigator award 2001 winner: Risk factors for lumbar disc degeneration: A 5-year prospective MRI study in asymptomatic individuals. Spine. 2002;27(2):125-34.

3. Farshad-Amacker NA, Hughes AP, Aichmair A, Herzog RJ, Farshad M. Determinants of evolution of endplate and disc degeneration in the lumbar spine: a multifactorial perspective. European Spine Journal. 2014;23(9):1863-8.

4. Schistad EI, Espeland A, Pedersen LM, Sandvik L, Gjerstad J, Roe C. Association between baseline IL-6 and 1-year recovery in lumbar radicular pain. European Journal of Pain (United Kingdom). 2014;18(10):1394-401.

5. Suri P, Boyko EJ, Goldberg J, Forsberg CW, Jarvik JG. Longitudinal associations between incident lumbar spine MRI findings and chronic low back pain or radicular symptoms: Retrospective analysis of data from the longitudinal assessment of imaging and disability of the back (LAIDBACK). BMC Musculoskeletal Disorders. 2014;15(1).

6. Vanek P, Bradac O, Saur K, Riha M. Factors influencing the outcome of surgical treatment of lumbar disc herniation. [Czech]. Ceska a Slovenska Neurologie a Neurochirurgie. 2010;73(2):157-63.

## No reference standard (LBP or activity limitation measure)

1. Abrishamkar S, Mahmoudkhani M, Aminmansour B, Mahabadi A, Jafari S. Does disk space degeneration according to Los Angeles and Modic scales have relation with recurrent disk herniation? Advanced Biomedical Research. 2014;3:220.

2. Albert HB, Briggs AM, Kent P, Byrhagen A, Hansen C, Kjaergaard K. The prevalence of MRI-defined spinal pathoanatomies and their association with modic changes in individuals seeking care for low back pain. European Spine Journal. 2011;20(8):1355-62.

3. Albert HB, Lambert P, Rollason J, Sorensen JS, Worthington T, Pedersen MB, et al. Does nuclear tissue infected with bacteria following disc herniations lead to Modic changes in the adjacent vertebrae? European Spine Journal. 2013;22(4):690-6.

4. Albert HB, Manniche C. Modic changes following lumbar disc herniation. European Spine Journal. 2007;16(7):977-82.

5. Arana E, Kovacs FM, Royuela A, Estremera A, Asenjo B, Sarasibar H, et al. Modic changes and associated features in Southern European chronic low back pain patients. Spine Journal: Official Journal of the North American Spine Society. 2011;11(5):402-11.

6. Arana E, Royuela A, Kovacs FM, Estremera A, Sarasibar H, Amengual G, et al. Lumbar spine: agreement in the interpretation of 1.5-T MR images by using the Nordic Modic Consensus Group classification form. Radiology. 2010;254(3):809-17.

7. Arndt J, Charles YP, Koebel C, Bogorin I, Steib JP. Bacteriology of degenerated lumbar intervertebral disks. Journal of Spinal Disorders & Techniques. 2012;25(7):E211-6.

8. Assheuer J, Lenz G, Lenz W, Gottschlich KW, Schulitz KP. Fat/water separation in the NMR tomogram. The imaging of bone marrow reactions in degenerative intervertebral disk changes. [German]. RoFo : Fortschritte auf dem Gebiete der Rontgenstrahlen und der Nuklearmedizin. 1987;147(1):58-63.

9. Battie MC, Haynor DR, Fisher LD, Gill K, Gibbons LE, Videman T. Similarities in degenerative findings on magnetic resonance images of the lumbar spines of identical twins. Journal of Bone & Joint Surgery - American Volume. 1995;77(11):1662-70.

10. Bram J, Zanetti M, Min K, Hodler J. MR abnormalities of the intervertebral disks and adjacent bone marrow as predictors of segmental instability of the lumbar spine. Acta Radiologica. 1998;39(1):18-23.

11. Carragee E, Alamin T, Cheng I, Franklin T, van den Haak E, Hurwitz E. Are first-time episodes of serious LBP associated with new MRI findings? Spine Journal: Official Journal of the North American Spine Society. 2006;6(6):624-35.

12. Carragee EJ, Don AS, Hurwitz EL, Cuellar JM, Carrino JA, Herzog R. 2009 ISSLS Prize Winner: Does discography cause accelerated progression of degeneration changes in the lumbar disc: a ten-year matched cohort study.[Erratum appears in Spine (Phila Pa 1976). 2010 Jun 15;35(14):1414 Note: Carrino, John [corrected to Carrino, John A]]. Spine. 2009;34(21):2338-45.

13. Cheung KMC, Samartzis D, Karppinen J, Mok FPS, Ho DWH, Fong DYT, et al. Intervertebral disc degeneration: New insights based on "skipped" level disc pathology. Arthritis and Rheumatism. 2010;62(8):2392-400.

14. Chung CB, Vande Berg BC, Tavernier T, Cotten A, Laredo JD, Vallee C, et al. End plate marrow changes in the asymptomatic lumbosacral spine: frequency, distribution and correlation with age and degenerative changes. Skeletal Radiology. 2004;33(7):399-404.

15. D'Aprile P, Tarantino A, Lorusso V, Brindicci D. Fat saturation technique and gadolinium in MRI of lumbar spinal degenerative disease. Neuroradiology Journal. 2006;19(5):654-71.

16. Dallaudiere B, Dautry R, Preux PM, Perozziello A, Lincot J, Schouman-Claeys E, et al. Comparison of apparent diffusion coefficient in spondylarthritis axial active inflammatory lesions and type 1 Modic changes. European Journal of Radiology. 2014;83(2):366-70.

17. De Roos A, Kressel H, Spritzer C, Dalinka M. MR imaging of marrow changes adjacent to end plates in degenerative lumbar disk disease. American Journal of Roentgenology. 1987;149(3):531-4.

18. Frobin W, Brinckmann P, Kramer M, Hartwig E. Height of lumbar discs measured from radiographs compared with degeneration and height classified from MR images. European Radiology. 2001;11(2):263-9.

19. Fu MC, Buerba RA, Long WD, 3rd, Blizzard DJ, Lischuk AW, Haims AH, et al. Interrater and intrarater agreements of magnetic resonance imaging findings in the lumbar spine: significant variability across degenerative conditions. Spine Journal: Official Journal of the North American Spine Society. 2014;14(10):2442-8.

20. Hayashi T, Daubs MD, Suzuki A, Scott TP, Phan KH, Ruangchainikom M, et al. Motion characteristics and related factors of Modic changes in the lumbar spine. Journal of Neurosurgery Spine. 2015;22(5):511-7.

21. Jarvik JG, Hollingworth W, Heagerty PJ, Haynor DR, Boyko EJ, Deyo RA. Three-year incidence of low back pain in an initially asymptomatic cohort: clinical and imaging risk factors. Spine. 2005;30(13):1541-8; discussion 9.

22. Jensen OK, Nielsen CV, Sorensen JS, Stengaard-Pedersen K. Type 1 Modic changes was a significant risk factor for 1-year outcome in sick-listed low back pain patients: a nested cohort study using magnetic resonance imaging of the lumbar spine. Spine Journal: Official Journal of the North American Spine Society. 2014;14(11):2568-81.

23. Jensen RK, Jensen TS, Kjaer P, Kent P. Can pathoanatomical pathways of degeneration in lumbar motion segments be identified by clustering MRI findings. BMC Musculoskeletal Disorders. 2013;14(198).

24. Johansen JV, Manniche C, Kjaer P. Vitamin D levels appear to be normal in Danish patients attending secondary care for low back pain and a weak positive correlation between serum level Vitamin D and Modic changes was demonstrated: a cross-sectional cohort study of consecutive patients with non-specific low back pain. BMC Musculoskeletal Disorders. 2013;14:78.

25. Kakitsubata Y, Nabeshima K, Kakitsubata S, Watanabe K. Evaluation of lumbar vertebral bone marrow changes with MR imaging. [Japanese]. Nihon Igaku Hoshasen Gakkai zasshi. 1993;Nippon acta radiologica. 53(11):1267-82.

26. Kanna RM, Shetty AP, Rajasekaran S. Patterns of lumbar disc degeneration are different in degenerative disc disease and disc prolapse magnetic resonance imaging analysis of 224 patients. Spine Journal: Official Journal of the North American Spine Society. 2014;14(2):300-7.

27. Karchevsky M, Schweitzer ME, Carrino JA, Zoga A, Montgomery D, Parker L. Reactive endplate marrow changes: a systematic morphologic and epidemiologic evaluation. Skeletal Radiology. 2005;34(3):125-9.

28. Karppinen J, Daavittila I, Solovieva S, Kuisma M, Taimela S, Natri A, et al. Genetic factors are associated with modic changes in endplates of lumbar vertebral bodies. Spine. 2008;33(11):1236-41.

29. Karppinen J, Mikkonen P, Kurunlahti M, Tervonen O, Paldanius M, Vasari P, et al. Chronic Chlamydia pneumoniae infection increases the risk of occlusion of lumbar segmental arteries of patients with sciatica: a 3-year follow-up study. Spine. 2003;28(15):E284-9.

30. Karppinen J, Paakko E, Paassilta P, Lohiniva J, Kurunlahti M, Tervonen O, et al. Radiologic phenotypes in lumbar MR imaging for a gene defect in the COL9A3 gene of type IX collagen. Radiology. 2003;227(1):143-8.

31. Kholin AB, Makarov AI, Leikin IB, Amelina OA, Zablotskii NU, Loban TG. Magnetic resonance tomography in the diagnosis of the neurological complications of lumbar osteochondrosis. [Russian]. Zhurnal nevropatologii i psikhiatrii imeni SS. 1996;Korsakova(Moscow, Russia : 1952 . 96 6):44-8.

32. Kholin AV. Ultra-low magnetic resonance tomography of thoracic and lumbar spine osteochondrosis. [Russian]. Klinicheskaia meditsina. 1994;72(3):40-4.

33. Kuisma M, Karppinen J, Niinimaki J, Kurunlahti M, Haapea M, Vanharanta H, et al. A three-year follow-up of lumbar spine endplate (Modic) changes. Spine. 2006;31(15):1714-8.

34. Kwon YM, Chin DK, Jin BH, Kim KS, Cho YE, Kuh SU. Long term efficacy of posterior lumbar interbody fusion with standard cages alone in lumbar disc diseases combined with modic changes. Journal of Korean Neurosurgical Society. 2009;46(4):322-7.

35. Lee JW, Choi SW, Park SH, Lee GY, Kang HS. MR-based outcome predictors of lumbar transforaminal epidural steroid injection for lumbar radiculopathy caused by herniated intervertebral disc. European Radiology. 2013;23(1):205-11.

36. Lee S-H, Bae JS. Comparison of clinical and radiological outcomes after automated open lumbar discectomy and conventional microdiscectomy: a prospective randomized trial. International journal of clinical and experimental medicine. 2015;8(8):12135-48.

37. Li Y, Lord E, Cohen Y, Ruangchainikom M, Wang B, Lv G, et al. Effects of sagittal endplate shape on lumbar segmental mobility as evaluated by kinetic magnetic resonance imaging. Spine. 2014;39(17):E1035-41.

38. Li Y, Samartzis D, Campbell DD, Cherny SS, Cheung KMC, Luk KDK, et al. Two subtypes of intervertebral disc degeneration distinguished by large-scale population-based study. Spine Journal: Official Journal of the North American Spine Society. 2016:5.

39. Liu ZZ, Chen JY, Zhong JL, Jiang XH, Cai ZX, Zhang Y, et al. Magnetic resonance imaging of lumbar intervertebral discs degeneration: Analysis of the imaging manifestations related to Modic changes. [Chinese]. Chinese Journal of Tissue Engineering Research. 2012;16(52):9737-43.

40. Ma Z, Ding WY, Shen Y, Sun YP, Yang DL, Xu JX. [The study on the relationship between modic change and disc height together with lumbar hyperosteogeny]. Chung-Hua Wai Ko Tsa Chih [Chinese Journal of Surgery]. 2013;51(7):610-4.

41. Maatta J, Kautiainen H, Leinonen V, Niinimaki J, Jarvenpaa S, Koskelainen T, et al. Association of Modic changes with health-related quality of life among patients referred to spine surgery. Scandinavian Journal of Pain. 2014;5(1):36-40.

42. Maatta JH, Karppinen JI, Luk KDK, Cheung KMC, Samartzis D. Phenotype profiling of Modic changes of the lumbar spine and its association with other MRI phenotypes: a large-scale population-based study. Spine Journal: Official Journal of the North American Spine Society. 2015;15(9):1933-42.

43. Maatta JH, Kraatari M, Wolber L, Niinimaki J, Wadge S, Karppinen J, et al. Vertebral endplate change as a feature of intervertebral disc degeneration: A heritability study. European Spine Journal. 2014;23(9):1856-62.

44. Maes R, Morrison WB, Parker L, Schweitzer ME, Carrino JA. Lumbar interspinous bursitis (Baastrup disease) in a symptomatic population: prevalence on magnetic resonance imaging. Spine. 2008;33(7):E211-5.

45. Maksymowicz H, Sasiadek M, Dusza B, Filarski J. Evaluation of CBASS sequence in degenerative disease of the lumbar spine based on analysis of consecutive 78 cases. Medical Science Monitor. 2004;10 Suppl 3:107-11.

46. Marshman LA, Metcalfe AV, Krishna M, Friesem T. Are high-intensity zones and Modic changes mutually exclusive in symptomatic lumbar degenerative discs? Journal of Neurosurgery Spine. 2010;12(4):351-6.

47. Mattam A, Sunny G. Correlation of Vitamin D and Body Mass Index with Modic Changes in Patients with Non-Specific Low Back Pain in a Sub-Tropical Asian Population. Asian Spine Journal. 2016;10(1):14-9.

48. Modic MT, Steinberg PM, Ross JS, Masaryk TJ, Carter JR. Degenerative disk disease: assessment of changes in vertebral body marrow with MR imaging. Radiology. 1988;166(1 Pt 1):193-9.

49. Mok FPS, Samartzis D, Karppinen J, Luk KDK, Fong DYT, Cheung KMC. ISSLS prize winner: Prevalence, determinants, and association of schmorl nodes of the lumbar spine with disc degeneration: A population-based study of 2449 individuals. Spine. 2010;35(21):1944-52.

50. Molla E, Marti-Bonmati L, Arana E, Martinez-Bisbal MC, Costa S. Magnetic resonance myelography evaluation of the lumbar spine end plates and intervertebral disks. Acta Radiologica. 2005;46(1):83-8.

51. Mostafavi SRS, Samimi K, Ashtiani FP, Fateh S. Association between lumbar modic changes and MRI finding of degenerative disc in patients with low back pain. [Persian]. Tehran University Medical Journal. 2016;73(10):732-8.

52. Motiei-Langroudi R, Sadeghian H, Seddighi AS. Clinical and magnetic resonance imaging factors which may predict the need for surgery in lumbar disc herniation. Asian Spine Journal. 2014;8(4):446-52.

53. Novosel'tsev SV, Malinovskii EL, Smirnov VV, Savvova M, Lebedeva VV. [Pathobiomechanical impairments of the vertebral column in intervertebral disk protrusion and herniation]. Vestnik Rentgenologii i Radiologii. 2011(5):34-9.

54. Peterson CK, Gatterman B, Carter JC, Humphreys BK, Weibel A. Inter- and intraexaminer reliability in identifying and classifying degenerative marrow (Modic) changes on lumbar spine magnetic resonance scans. Journal of Manipulative & Physiological Therapeutics. 2007;30(2):85-90.

55. Quack C, Schenk P, Laeubli T, Spillmann S, Hodler J, Michel BA, et al. Do MRI findings correlate with mobility tests? An explorative analysis of the test validity with regard to structure. European Spine Journal. 2007;16(6):803-12.

56. Rajasekaran S, Venkatadass K, Naresh Babu J, Ganesh K, Shetty AP. Pharmacological enhancement of disc diffusion and differentiation of healthy, ageing and degenerated discs : Results from in-vivo serial post-contrast MRI studies in 365 human lumbar discs. European Spine Journal. 2008;17(5):626-43.

57. Rankine JJ, Hutchinson CE, Hughes DG. MRI of lumbar spondylosis: a comparison of sagittal T2 weighted and three sequence examinations. British Journal of Radiology. 1997;70(839):1112-21.

58. Saberi H, Rahimi L, Jahani L. A comparative MRI study of upper and lower lumbar motion segments in patients with low back pain. Journal of Spinal Disorders & Techniques. 2009;22(7):507-10.

59. Saborido MC, Marti-Bonmati L, Nogues P, Casillas C. MR findings in lumbar spine of patients with failed chemonucleolysis therapy. [Spanish]. Radiologia. 1998;40(3):181-6.

60. Saleem H, Raza S, Slehria AUR. The frequency of modic changes in lumbosacral spine in patients with low back pain. Pakistan Journal of Medical and Health Sciences. 2013;7(2).

61. Samartzis D, Karppinen J, Chan D, Luk KD, Cheung KM. The association of lumbar intervertebral disc degeneration on magnetic resonance imaging with body mass index in overweight and obese adults: a population-based study. Arthritis & Rheumatism. 2012;64(5):1488-96.

62. Savvopoulou V, Maris TG, Koureas A, Gouliamos A, Moulopoulos LA. Degenerative endplate changes of the lumbosacral spine: dynamic contrast-enhanced MRI profiles related to age, sex, and spinal level. Journal of Magnetic Resonance Imaging. 2011;33(2):382-9.

63. Schenk P, Laubli T, Hodler J, Klipstein A. Magnetic resonance imaging of the lumbar spine: findings in female subjects from administrative and nursing professions. Spine. 2006;31(23):2701-6.

64. Schmid G, Witteler A, Willburger R, Kuhnen C, Jergas M, Koester O. Lumbar Disk Herniation: Correlatlon of Histologic Findings with Marrow Signal Intensity Changes in Vertebral Endplates at MR Imaging. Radiology. 2004;231(2):352-8.

65. Seidl Z, Obenberger J, Danes J, Vitak T, Peterova V, Vaneckova M. MRI differential diagnostics of bone marrow signal changes. Ceska Radiologie. 2001;55(2):75-9.

66. Sharma A, Parsons M, Pilgram T. Temporal interactions of degenerative changes in individual components of the lumbar intervertebral discs: a sequential magnetic resonance imaging study in patients less than 40 years of age. Spine. 2011;36(21):1794-800.

67. Tosun O, Fidan F, Erdil F, Tosun A, Karaoglanoglu M, Ardicoglu O. Assessment of lumbar vertebrae morphology by magnetic resonance imaging in osteoporosis. Skeletal Radiology. 2012;41(12):1583-90.

68. Toyoda K, Ida M, Murakami Y, Harada J, Tada S. MR imaging of degenerative lumbar disc disease emphasizing on signal intensity changes in vertebral body. [Japanese]. Nihon Igaku Hoshasen Gakkai zasshi. 1992;Nippon acta radiologica. 52(12):1611-9.

69. Villarreal-Arroyo M, Mejia-Herrera JC, Larios-Forte MC. [Incidence of Modic degenerative changes in patients with chronic lumbar pain at Monterrey Regional ISSSTE Hospital]. [Spanish]. Acta ortopedica mexicana. 2012;26(3):180-4.

70. Vredeveld T, Teitsma XM, Mert A, Van der Wurff P. Prevalence of modic changes in active duty military men with lumbar disk herniation who were scheduled for surgery. Journal of Manipulative & Physiological Therapeutics. 2012;35(8):622-8.

71. Wang Y, Videman T, Battie MC. Modic changes: prevalence, distribution patterns, and association with age in white men. Spine Journal: Official Journal of the North American Spine Society. 2012;12(5):411-6.

72. Wang Y, Videman T, Niemelainen R, Battie MC. Quantitative measures of modic changes in lumbar spine magnetic resonance imaging: intra- and inter-rater reliability. Spine. 2011;36(15):1236-43.

73. Wang Y, Yin R. Distribution of Schmorl's nodes in lumbar spine and their relationship with lumbar disc degeneration. [Chinese]. National Medical Journal of China. 2014;94(35):2736-9.

74. Wang ZX, An P, Li Y, Kim SH. Analysis of lumbar spine MRI in asymptomatic Chinese adults. [Chinese]. Chinese Journal of Interventional Imaging and Therapy. 2012;9(5):371-5.

75. Weishaupt D, Zanetti M, Hodler J, Boos N. MR imaging of the lumbar spine: prevalence of intervertebral disk extrusion and sequestration, nerve root compression, end plate abnormalities, and osteoarthritis of the facet joints in asymptomatic volunteers. Radiology. 1998;209(3):661-6.

76. Wilkens P, Storheim K, Scheel I, Berg L, Espeland A. No effect of 6-month intake of glucosamine sulfate on Modic changes or high intensity zones in the lumbar spine: sub-group analysis of a randomized controlled trial. Journal of Negative Results in Biomedicine. 2012;11:13.

77. Wu HL, Ding WY, Shen Y, Zhang YZ, Guo JK, Sun YP, et al. Prevalence of vertebral endplate modic changes in degenerative lumbar scoliosis and its associated factors analysis. Spine. 2012;37(23):1958-64.

78. Xiao B, Tian W, Zhao DH, Wu CA, Wang N, Zhang YZ. Relationship between 666C>T polymorphism of TIMP-1 and lumbar intervertebral disc degeneration. [Chinese]. National Medical Journal of China. 2010;90(41):2939-42.

79. Xu L, Chu B, Feng Y, Xu F, Zou Y-F. Modic changes in lumbar spine: prevalence and distribution patterns of end plate oedema and end plate sclerosis. British Journal of Radiology. 2016;89(1060):20150650.

80. Yong PY, Alias NAA, Shuaib IL. Correlation of clinical presentation, radiography, and magnetic resonance imaging for low back pain - A preliminary survey. Journal of the Hong Kong College of Radiologists. 2003;6(3):144-51.

81. Zhang K, Li M, Pei X, Yuan H. Regression between MR findings of lumbar elements and chronic low back pain. [Chinese]. Chinese Journal of Radiology (China). 2014;48(12):1019-23.

82. Zhang R, Yin BS, Yang Q, Yang LY, Wang SY. Distribution of Modic changes of lumbar endplate in patients suffering from low back pain or radiculopathy and its related factors. [Chinese]. Journal of Dalian Medical University. 2012;34(2):161-5.

83. Zhao J, Qu DB, Chen JT, Jiang JM, Jiang H, Jin DD. Prosthetic disc nucleus replacement for lumbar intervertebral disc herniation. [Chinese]. Journal of Clinical Rehabilitative Tissue Engineering Research. 2007;11(16):3127-30.

## No Modic changes

1. Akgol G, Kamanli A, Ozgocmen S. Evidence for inflammation-induced bone loss in non-radiographic axial spondyloarthritis. Rheumatology. 2014;53(3):497-501.

2. Ali A, Khan SA, Aurangzeb A, Ahmed E, Ali G, Muhammad G, et al. Lumbar disc herniation in patients with chronic backache. Journal of Ayub Medical College, Abbottabad: JAMC. 2013;25(3-4):68-70.

3. Altinkaya N, Yildirim T, Demir S, Alkan O, Sarica FB. Factors associated with the thickness of the ligamentum flavum: is ligamentum flavum thickening due to hypertrophy or buckling? Spine. 2011;36(16):E1093-7.

4. Arana E, Kovacs FM, Royuela A, Estremera A, Sarasibar H, Amengual G, et al. Influence of nomenclature in the interpretation of lumbar disk contour on MR imaging: a comparison of the agreement using the combined task force and the nordic nomenclatures. Ajnr: American Journal of Neuroradiology. 2011;32(6):1143-8.

5. Assietti R, Morosi M, Block JE. Intradiscal electrothermal therapy for symptomatic internal disc disruption: 24-month results and predictors of clinical success. Journal of Neurosurgery Spine. 2010;12(3):320-6.

6. Battie MC, Levalahti E, Videman T, Burton K, Kaprio J. Heritability of lumbar flexibility and the role of disc degeneration and body weight. Journal of Applied Physiology. 2008;104(2):379-85.

7. Battie MC, Videman T, Gill K, Moneta GB, Nyman R, Kaprio J, et al. 1991 Volvo Award in Clinical Sciences: Smoking and lumbar intervertebral disc degeneration: An MRI study of identical twins. Spine. 1991;16(9):1015-21.

8. Battie MC, Videman T, Levalahti E, Gill K, Kaprio J. Heritability of low back pain and the role of disc degeneration. Pain. 2007;131(3):272-80.

9. Bechara BP, Agarwal V, Boardman J, Perera S, Weiner DK, Vo N, et al. Correlation of pain with objective quantification of magnetic resonance images in older adults with chronic low back pain. Spine. 2014;39(6):469-75.

10. Bennett AN, McGonagle D, O'Connor P, Hensor EM, Sivera F, Coates LC, et al. Severity of baseline magnetic resonance imaging-evident sacroiliitis and HLA-B27 status in early inflammatory back pain predict radiographically evident ankylosing spondylitis at eight years. Arthritis & Rheumatism. 2008;58(11):3413-8.

11. Biluts H, Munie T, Abebe M. Review of lumbar disc diseases at Tikur Anbessa Hospital. Ethiopian Medical Journal. 2012;50(1):57-65.

12. Birney TJ, White Jr JJ, Berens D, Kuhn G. Comparison of MRI and discography in the diagnosis of lumbar degenerative disc disease. Journal of Spinal Disorders. 1992;5(4):417-23.

13. Boleaga-Duran B, Fiesco-Gomez LE. Degenerative disease of the lumbar spine. Clinical and magnetic resonance imaging correlation. [Spanish]. Cirugia y cirujanos. 2006;74(2):101-5.

14. Buirski G. Magnetic resonance signal patterns of lumbar discs in patients with low back pain: A prospective study with discographic correlation. Spine. 1992;17(10):1199-204.

15. Burton AK, Battie MC, Gibbons L, Videman T, Tillotson KM. Lumbar disc degeneration and sagittal flexibility. Journal of Spinal Disorders. 1996;9(5):418-24.

16. Capel A, Medina FS, Medina D, Gomez S. Magnetic resonance study of lumbar disks in female dancers. American Journal of Sports Medicine. 2009;37(6):1208-13.

17. Carragee EJ, Paragioudakis SJ, Khurana S. Lumbar high-intensity zone and discography in subjects without low back problems. Spine. 2000;25(23):2987-92.

18. Carrino JA, Lurie JD, Tosteson ANA, Tosteson TD, Carragee EJ, Kaiser J, et al. Lumbar spine: Reliability of MR imaging findings. Radiology. 2009;250(1):161-70.

19. Castro WHM, Assheuer J, Schulitz KP. Haemodynamic changes in lumbar nerve root entrapment due to stenosis and/or herniated disc of the lumbar spinal canal a magnetic resonance imaging study. European Spine Journal. 1995;4(4):220-5.

20. Caterini R, Mancini F, Bisicchia S, Maglione P, Farsetti P. The correlation between exaggerated fluid in lumbar facet joints and degenerative spondylolisthesis: prospective study of 52 patients. Journal of Orthopaedics & Traumatology. 2011;12(2):87-91.

21. Chen B, Lavender S, Andersson GBJ. The magnetic resonance imaging of the lumbar spine in out-patients with low back pain. Journal of Musculoskeletal Research. 2001;5(4):261-7.

22. Cheng F, You J, Rampersaud YR. Relationship between spinal magnetic resonance imaging findings and candidacy for spinal surgery. Canadian Family Physician. 2010;56(9):e323-e30.

23. Cheung KM, Chan D, Karppinen J, Chen Y, Jim JJ, Yip SP, et al. Association of the Taq I allele in vitamin D receptor with degenerative disc disease and disc bulge in a Chinese population. Spine. 2006;31(10):1143-8.

24. Cheung KM, Karppinen J, Chan D, Ho DW, Song YQ, Sham P, et al. Prevalence and pattern of lumbar magnetic resonance imaging changes in a population study of one thousand forty-three individuals. Spine. 2009;34(9):934-40.

25. Cienciala J, Chaloupka R, Repko M, Krbec M. Dynamic neutralization using the dynesys system for treatment of degenerative disc disease of the lumbar spine. [Czech]. Acta Chirurgiae Orthopaedicae et Traumatologiae Cechoslovaca. 2010;77(3):203-8.

26. Cihangiroglu M, Yildirim H, Bozgeyik Z, Senol U, Ozdemir H, Topsakal C, et al. Observer variability based on the strength of MR scanners in the assessment of lumbar degenerative disc disease. European Journal of Radiology. 2004;51(3):202-8.

27. Cohn EL, Maurer EJ, Keats TE, Dussault RG, Kaplan PA. Plain film evaluation of degenerative disk disease at the lumbosacral junction. Skeletal Radiology. 1997;26(3):161-6.

28. Colombini A, Brayda-Bruno M, Ferino L, Lombardi G, Maione V, Banfi G, et al. Gender Differences in the VDR-FokI Polymorphism and Conventional Non-Genetic Risk Factors in Association with Lumbar Spine Pathologies in an Italian Case-Control Study. International Journal of Molecular Sciences. 2015;16(2):3722-39.

29. Colombini A, Brayda-Bruno M, Lombardi G, Croiset SJ, Vrech V, Maione V, et al. Fokl polymorphism in the vitamin D receptor gene (VDR) and its association with lumbar spine pathologies in the Italian population: A case-control study. PLoS ONE. 2014;9(5).

30. Cui FZ, Cui JL, Wang SL, Du CG, Liu JC, Sun YC. Whole body diffusion weighted imaging pattern of normal bone marrow. [Chinese]. Chinese Journal of Radiology (China). 2012;46(4):340-4.

31. Donescu OS, Battie MC, Videman T. The influence of magnetic resonance imaging findings of degenerative disease on dual-energy X-ray absorptiometry measurements in middle-aged men. Acta Radiologica. 2007;48(2):193-9.

32. Dong DM, Yao M, Liu B, Sun CY, Jiang YQ, Wang YS. Association between the -1306C/T polymorphism of matrix metalloproteinase-2 gene and lumbar disc disease in Chinese young adults. European Spine Journal. 2007;16(11):1958-61.

33. Dora C, Schmid MR, Elfering A, Zanetti M, Hodler J, Boos N. Lumbar disk herniation: Do MR imaging findings predict recurrence after surgical diskectomy? Radiology. 2005;235(2):562-7.

34. el Barzouhi A, Vleggeert-Lankamp CLAM, Lycklama a Nijeholt GJ, Van der Kallen BF, van den Hout WB, Verwoerd AJH, et al. Magnetic Resonance Imaging Interpretation in Patients with Sciatica Who Are Potential Candidates for Lumbar Disc Surgery. PLoS ONE. 2013;8(7).

35. El-Badry A, Belal T. Results of lumbar discectomy in patients with recurrent disc herniation compared to patients with primary disc herniation. Egyptian Journal of Neurology, Psychiatry and Neurosurgery. 2012;49(4):335-40.

36. Erdem LO, Erdem CZ, Acikgoz B, Gundogdu S. Degenerative disc disease of the lumbar spine: a prospective comparison of fast T1-weighted fluid-attenuated inversion recovery and T1-weighted turbo spin echo MR imaging. European Journal of Radiology. 2005;55(2):277-82.

37. Erkintalo MO, Salminen JJ, Alanen AM, Paajanen HEK, Kormano MJ. Development of degenerative changes in the lumbar intervertebral disk: Results of a prospective MR imaging study in adolescents with and without low-back pain. Radiology. 1995;196(2):529-33.

38. Eser B, Cora T, Eser O, Kalkan E, Haktanir A, Erdogan MO, et al. Association of the polymorphisms of vitamin D receptor and aggrecan genes with degenerative disc disease. Genetic Testing & Molecular Biomarkers. 2010;14(3):313-7.

39. Eser O, Eser B, Cosar M, Erdogan MO, Aslan A, Yildiz H, et al. Short aggrecan gene repetitive alleles associated with lumbar degenerative disc disease in Turkish patients. Genetics & Molecular Research. 2011;10(3):1923-30.

40. Eskola PJ, Kjaer P, Daavittila IM, Solovieva S, Okuloff A, Sorensen JS, et al. Genetic risk factors of disc degeneration among 12-14-year-old Danish children: A population study. International Journal of Molecular Epidemiology and Genetics. 2010;1(2):158-65.

41. Eskola PJ, Kjaer P, Sorensen JS, Okuloff A, Wedderkopp N, Daavittila I, et al. Gender difference in genetic association between IL1A variant and early lumbar disc degeneration: a three-year follow-up. International Journal of Molecular Epidemiology and Genetics. 2012;3(3):195-204.

42. Fu L, France A, Xie Y, Fang K, Gan Y, Zhang P. Functional and radiological outcomes of semi-rigid dynamic lumbar stabilization adjacent to single-level fusion after 2 years. Archives of Orthopaedic & Trauma Surgery. 2014;134(5):605-10.

43. Fujiwara A, Tamai K, An HS, Kurihashi A, Lim TH, Yoshida H, et al. The relationship between disc degeneration, facet joint osteoarthritis, and stability of the degenerative lumbar spine. Journal of Spinal Disorders. 2000;13(5):444-50.

44. Fujiwara A, Tamai K, Yamato M, An HS, Yoshida H, Saotome K, et al. The relationship between facet joint osteoarthritis and disc degeneration of the lumbar spine: An MRI study. European Spine Journal. 1999;8(5):396-401.

45. Greenspan A, Amparo EG, Gorczyca DP, Montesano PX. Is there a role for diskography in the era of magnetic resonance imaging? Prospective correlation and quantitative analysis of computed tomography-diskography, magnetic resonance imaging, and surgical findings. Journal of Spinal Disorders. 1992;5(1):26-31.

46. Grenier N, Kressel HY, Schiebler ML, Grossman RI, Dalinka MK. Normal and degenerative posterior spinal structures: MR imaging. Radiology. 1987;165(2):517-25.

47. Griffith JF, Wang YXJ, Antonio GE, Choi KC, Yu A, Ahuja AT, et al. Modified Pfirrmann grading system for lumbar intervertebral disc degeneration. Spine. 2007;32(24):E708-E12.

48. Guo XH, Chen ZQ, Liu N, Guo ZQ, Qi Q, Li WS, et al. Comparison between two types of "scheuermann disease-like people": Thoracolumbar disc herniation patients and healthy volunteers with radiological signs of scheuermann's disease. Chinese Medical Journal. 2014;127(22):3862-6.

49. Hamanishi C, Kawabata T, Yosii T, Tanaka S. Schmorl's nodes on magnetic resonance imaging. Their incidence and clinical relevance. Spine. 1994;19(4):450-3.

50. Han IB, Ropper AE, Teng YD, Shin DA, Jeon YJ, Park HM, et al. Association between VEGF and eNOS gene polymorphisms and lumbar disc degeneration in a young Korean population. Genetics & Molecular Research. 2013;12(3):2294-305.

51. Hancock MJ, Battie MC, Videman T, Gibbons L. The role of back injury or trauma in lumbar disc degeneration: an exposure-discordant twin study. Spine. 2010;35(21):1925-9.

52. Hangai M, Kaneoka K, Hinotsu S, Shimizu K, Okubo Y, Miyakawa S, et al. Lumbar intervertebral disk degeneration in athletes. American Journal of Sports Medicine. 2009;37(1):149-55.

53. Hangai M, Kaneoka K, Kuno S, Hinotsu S, Sakane M, Mamizuka N, et al. Factors associated with lumbar intervertebral disc degeneration in the elderly. Spine Journal. 2008;8(5):732-40.

54. Harada A, Okuizumi H, Miyagi N, Genda E. Correlation between bone mineral density and intervertebral disc degeneration. Spine. 1998;23(8):857-61; discussion 62.

55. Harisankar CNB, Mittal BR, Bhattacharya A, Singh P, Sen R. Utility of single photon emission computed tomography/computed tomography imaging in evaluation of chronic low back pain. Indian Journal of Nuclear Medicine. 2012;27(3):156-63.

56. Hebelka H, Brisby H, Hansson T. Comparison between pain at discography and morphological disc changes at axial loaded MRI in patients with low back pain. European Spine Journal. 2014;23(10):2075-82.

57. Heithoff KB, Gundry CR, Burton CV, Winter RB. Juvenile discogenic disease. Spine. 1994;19(3):335-40.

58. Higashino K, Matsui Y, Yagi S, Takata Y, Goto T, Sakai T, et al. The alpha2 type IX collagen tryptophan polymorphism is associated with the severity of disc degeneration in younger patients with herniated nucleus pulposus of the lumbar spine. International Orthopaedics. 2007;31(1):107-11.

59. Hoff E, Strube P, Gross C, Putzier M. Sequestrectomy with additional transpedicular dynamic stabilization for the treatment of lumbar disc herniation: no clinical benefit after 10 years follow-up. Spine. 2013;38(11):887-95.

60. Hong CH, Park JS, Jung KJ, Kim WJ. Measurement of the normal lumbar intervertebral disc space using magnetic resonance imaging. Asian Spine Journal. 2010;4(1):1-6.

61. Hoppe S, Quirbach S, Mamisch TC, Krause FG, Werlen S, Benneker LM. Axial T2 mapping in intervertebral discs: a new technique for assessment of intervertebral disc degeneration. European Radiology. 2012;22(9):2013-9.

62. Hsu K, Zucherman J, Shea W, Kaiser J, White A, Schofferman J, et al. High lumbar disc degeneration. Incidence and etiology. Spine. 1990;15(7):679-82.

63. Hung YJ, Shih TTF, Chen BB, Hwang YH, Ma LP, Huang WC, et al. The dose-response relationship between cumulative lifting load and lumbar disk degeneration based on magnetic resonance imaging findings. Physical therapy. 2014;94(11):1582-93.

64. Ikata T, Morita T, Katoh S, Tachibana K, Maoka H. Lesions of the lumbar posterior end plate in children and adolescents. An MRI study. Journal of Bone & Joint Surgery - British Volume. 1995;77(6):951-5.

65. Jang SY, Kong MH, Hymanson HJ, Jin TK, Song KY, Wang JC. Radiographic parameters of segmental instability in lumbar spine using kinetic MRI. Journal of Korean Neurosurgical Society. 2009;45(1):24-31.

66. Jensen MC, Brant-Zawadzki MN, Obuchowski N, Modic MT, Malkasian D, Ross JS. Magnetic resonance imaging of the lumbar spine in people without back pain. New England Journal of Medicine. 1994;331(2):69-73.

67. Jim JJ, Noponen-Hietala N, Cheung KM, Ott J, Karppinen J, Sahraravand A, et al. The TRP2 allele of COL9A2 is an age-dependent risk factor for the development and severity of intervertebral disc degeneration. Spine. 2005;30(24):2735-42.

68. Kader DF, Wardlaw D, Smith FW. Correlation between the MRI changes in the lumbar multifidus muscles and leg pain. Clinical Radiology. 2000;55(2):145-9.

69. Kanamori M, Nobukiyo M, Suzuki K, Yasuda T, Hori T. Clinical validity of a new T2-weighted MRI-based grading system for lumbar disc degeneration. International Medical Journal. 2013;20(4):466-9.

70. Kaneoka K, Shimizu K, Hangai M, Okuwaki T, Mamizuka N, Sakane M, et al. Lumbar intervertebral disk degeneration in elite competitive swimmers: A case control study. American Journal of Sports Medicine. 2007;35(8):1341-5.

71. Karadimas EJ, Siddiqui M, Smith FW, Wardlaw D. Positional MRI changes in supine versus sitting postures in patients with degenerative lumbar spine. Journal of Spinal Disorders & Techniques. 2006;19(7):495-500.

72. Kawaguchi Y, Kanamori M, Ishihara H, Ohmori K, Matsui H, Kimura T. The association of lumbar disc disease with vitamin-D receptor gene polymorphism. Journal of Bone and Joint Surgery - Series A. 2002;84(11):2022-8.

73. Kawaguchi Y, Osada R, Kanamori M, Ishihara H, Ohmori K, Matsui H, et al. Association between an aggrecan gene polymorphism and lumbar disc degeneration. Spine. 1999;24(23):2456-60.

74. Kelempisioti A, Eskola PJ, Okuloff A, Karjalainen U, Takatalo J, Daavittila I, et al. Genetic susceptibility of intervertebral disc degeneration among young Finnish adults. BMC Medical Genetics. 2011;12(153).

75. Keorochana G, Taghavi CE, Lee KB, Yoo JH, Liao JC, Fei Z, et al. Effect of sagittal alignment on kinematic changes and degree of disc degeneration in the lumbar spine: an analysis using positional MRI. Spine. 2011;36(11):893-8.

76. Keorochana G, Taghavi CE, Tzeng ST, Morishita Y, Yoo JH, Lee KB, et al. Magnetic resonance imaging grading of interspinous ligament degeneration of the lumbar spine and its relation to aging, spinal degeneration, and segmental motion: Clinical article. Journal of Neurosurgery: Spine. 2010;13(4):494-9.

77. Kim HJ, Suh BG, Lee DB, Lee GW, Kim DW, Kang KT, et al. The influence of pain sensitivity on the symptom severity in patients with lumbar spinal stenosis. Pain Physician. 2013;16(2):135-44.

78. Kim SJ, Lee TH, Lim SM. Prevalence of disc degeneration in asymptomatic Korean subjects. Part 1: Lumbar spine. Journal of Korean Neurosurgical Society. 2013;53(1):31-8.

79. Kitab SA, Alsulaiman AM, Benzel EC. Anatomic radiological variations in developmental lumbar spinal stenosis: a prospective, control-matched comparative analysis. Spine Journal: Official Journal of the North American Spine Society. 2014;14(5):808-15.

80. Kong MH, Morishita Y, He W, Miyazaki M, Zhang H, Wu G, et al. Lumbar segmental mobility according to the grade of the disc, the facet joint, the muscle, and the ligament pathology by using kinetic magnetic resonance imaging. Spine. 2009;34(23):2537-44.

81. Kumar A, Varghese M, Mohan D, Mahajan P, Gulati P, Kale S. Effect of whole-body vibration on the low back: A study of tractor- driving farmers in North India. Spine. 1999;24(23):2506-15.

82. Kumar R, Kumar V, Das NK, Behari S, Mahapatra AK. Adolescent lumbar disc disease: findings and outcome. Childs Nervous System. 2007;23(11):1295-9.

83. Lakadamyali H, Tarhan NC, Ergun T, Cakir B, Agildere AM. STIR sequence for depiction of degenerative changes in posterior stabilizing elements in patients with lower back pain. AJR American Journal of Roentgenology. 2008;191(4):973-9.

84. Lam KS, Carlin D, Mulholland RC. Lumbar disc high-intensity zone: The value and significance of provocative discography in the determination of the discogenic pain source. European Spine Journal. 2000;9(1):36-41.

85. Lee CS, Hwang CJ, Lee SW, Ahn YJ, Kim YT, Lee DH, et al. Risk factors for adjacent segment disease after lumbar fusion. European Spine Journal. 2009;18(11):1637-43.

86. Lee SH, Daffner SD, Wang JC. Does lumbar disk degeneration increase segmental mobility in vivo? Segmental motion analysis of the whole lumbar spine using kinetic MRI. Journal of Spinal Disorders & Techniques. 2014;27(2):111-6.

87. Lenz GP, Assheuer J, Lenz W, Gottschlich KW. New aspects of lumbar disc disease. MR imaging and histological findings. Archives of Orthopaedic & Trauma Surgery. 1990;109(2):75-82.

88. Li YH, Sun WJ, Zhao ZG, Sui HJ, Yang Y. Correlation of ADC values in lumbar intervertebral discs (1-4) and lumbar artery status with intervertebral disc degeneration. [Chinese]. Academic Journal of Second Military Medical University. 2010;31(2):169-72.

89. Lin WP, Lin JH, Chen XW, Wu CY, Zhang LQ, Huang ZD, et al. Interleukin-10 promoter polymorphisms associated with susceptibility to lumbar disc degeneration in a Chinese cohort. Genetics & Molecular Research. 2011;10(3):1719-27.

90. Linson MA, Crowe CH. Comparison of magnetic resonance imaging and lumbar discography in the diagnosis of disc degeneration. Clinical Orthopaedics and Related Research. 1990(250):160-3.

91. Liuke M, Solovieva S, Lamminen A, Luoma K, Leino-Arjas P, Luukkonen R, et al. Disc degeneration of the lumbar spine in relation to overweight. International Journal of Obesity. 2005;29(8):903-8.

92. Livshits G, Ermakov S, Popham M, MacGregor AJ, Sambrook PN, Spector TD, et al. Evidence that bone mineral density plays a role in degenerative disc disease: The UK twin spine study. Annals of the Rheumatic Diseases. 2010;69(12):2102-6.

93. Livshits G, Popham M, Malkin I, Sambrook PN, Macgregor AJ, Spector T, et al. Lumbar disc degeneration and genetic factors are the main risk factors for low back pain in women: the UK Twin Spine Study. Annals of the Rheumatic Diseases. 2011;70(10):1740-5.

94. Luoma K, Riihimaki H, Luukkonen R, Raininko R, Viikari-Juntura E, Lamminen A. Low back pain in relation to lumbar disc degeneration. Spine. 2000;25(4):487-92.

95. Luoma K, Riihimaki H, Raininko R, Luukkonen R, Lamminen A, Viikari-Juntura E. Lumbar disc degeneration in relation to occupation. Scandinavian Journal of Work, Environment and Health. 1998;24(5):358-66.

96. Luoma K, Vehmas T, Raininko R, Luukkonen R, Riihimaki H. Lumbosacral Transitional Vertebra: Relation to Disc Degeneration and Low Back Pain. Spine. 2004;29(2):200-5.

97. Luoma K, Vehmas T, Riihimaki H, Raininko R. Disc height and signal intensity of the nucleus pulposus on magnetic resonance imaging as indicators of lumbar disc degeneration. Spine. 2001;26(6):680-6.

98. MacGregor AJ, Andrew T, Sambrook PN, Spector TD. Structural, Psychological, and Genetic Influences on Low Back and Neck Pain: A Study of Adult Female Twins. Arthritis Care and Research. 2004;51(2):160-7.

99. Malberg MI. A new system of classification for spinal injuries. Spine Journal: Official Journal of the North American Spine Society. 2001;1(1):18-25; discussion 125.

100. Maldjian C, Adam RJ, Akhtar N, Maldjian JA, Bonakdarpour A, Boyko O. Volume (three-dimensional) fast spin-echo imaging of the lumbar spine. Academic Radiology. 1999;6(6):339-42.

101. Marzo-Ortega H, McGonagle D, O'Connor P, Hensor EMA, Bennett AN, Green MJ, et al. Baseline and 1-year magnetic resonance imaging of the sacroiliac joint and lumbar spine in very early inflammatory back pain. Relationship between symptoms, HLA-B27 and disease extent and persistence. Annals of the Rheumatic Diseases. 2009;68(11):1721-7.

102. Masharawi Y, Nadaf N. The effect of non-weight bearing group-exercising on females with non-specific chronic low back pain: a randomized single blind controlled pilot study. Journal of Back & Musculoskeletal Rehabilitation. 2013;26(4):353-9.

103. Matsui H, Kanamori M, Ishihara H, Yudoh K, Naruse Y, Tsuji H. Familial predisposition for lumbar degenerative disc disease. Spine. 1998;23(9):1029-34.

104. Matsumoto M, Okada E, Toyama Y, Fujiwara H, Momoshima S, Takahata T. Tandem age-related lumbar and cervical intervertebral disc changes in asymptomatic subjects. European Spine Journal. 2013;22(4):708-13.

105. McAfee PC, Fedder IL, Saiedy S, Shucosky EM, Cunningham BW. Experimental design of total disk replacement - Experience with a prospective randomized study of the SB Charite. Spine. 2003;28(20 SUPPL.):S153-S62.

106. Michopoulou S, Costaridou L, Vlychou M, Speller R, Todd-Pokropek A. Texture-based quantification of lumbar intervertebral disc degeneration from conventional T2-weighted MRI. Acta Radiologica. 2011;52(1):91-8.

107. Milette PC, Fontaine S, Lepanto L, Cardinal E, Breton G. Differentiating lumbar disc protrusions, disc bulges, and discs with normal contour but abnormal signal intensity: Magnetic resonance imaging with discographic correlations. Spine. 1999;24(1):44-53.

108. Milicic G, Krolo I, Anticevic D, Roic G, Zadravec D, Bojic D, et al. Causal connection of non-specific low back pain and disc degeneration in children with transitional vertebra and/or Spina bifida occulta: role of magnetic resonance--prospective study. Collegium Antropologicum. 2012;36(2):627-33.

109. Min HK, Hymanson HJ, Kwan YS, Dong KC, Yong EC, Do HY, et al. Kinetic magnetic resonance imaging analysis of abnormal segmental motion of the functional spine unit: Clinical article. Journal of Neurosurgery: Spine. 2009;10(4):357-65.

110. Min S, Nakazato K, Okada T, Ochi E, Hiranuma K. The cartilage intermediate layer protein gene is associated with lumbar disc degeneration in collegiate judokas. International Journal of Sports Medicine. 2009;30(9):691-4.

111. Min SK, Nakazato K, Yamamoto Y, Gushiken K, Fujimoto H, Fujishiro H, et al. Cartilage intermediate layer protein gene is associated with lumbar disc degeneration in male, but not female, collegiate athletes. American Journal of Sports Medicine. 2010;38(12):2552-7.

112. Monti C, Busacca M, Bettini N, Moio A, Bianco T. Modern diagnostic imaging of lumbar spondylosis. Chirurgia Degli Organi di Movimento. 1994;79(1):19-28.

113. Moreno P, Boulot J. Comparative study of short-term results between total artificial disc prosthesis and anterior lumbar interbody fusion. [French]. Revue de chirurgie orthopedique et reparatrice de l'appareil moteur. 2008;94(3):282-8.

114. Moustarhfir M, Bresson B, Koch P, Perozziello A, Barreau G, Schouman-Claeys E, et al. MR imaging of Schmorl's nodes: Imaging characteristics and epidemio-clinical relationships. Diagnostic and Interventional Imaging. 2016;97(4):411-7.

115. Nagashima M, Abe H, Amaya K, Matsumoto H, Yanaihara H, Nishiwaki Y, et al. A method for quantifying intervertebral disc signal intensity on T2-weighted imaging. Acta Radiologica. 2012;53(9):1059-65.

116. Nagashima M, Abe H, Amaya K, Matsumoto H, Yanaihara H, Nishiwaki Y, et al. Risk factors for lumbar disc degeneration in high school American football players: a prospective 2-year follow-up study. American Journal of Sports Medicine. 2013;41(9):2059-64.

117. Nagashima M, Abe H, Amaya K, Matsumoto H, Yanaihara H, Nishiwaki Y, et al. Risk factors for lumbar disc degeneration in high school American football players: a prospective 2-year follow-up study. The American journal of sports medicine. 2013;41(9):2059-64.

118. Nam WD, Chang BS, Lee CK, Cho JH. Clinical and radiological predictive factors to be related with the degree of lumbar back muscle degeneration: difference by gender. Clinics in Orthopedic Surgery. 2014;6(3):318-23.

119. Nanjo Y, Morio Y, Nagashima H, Hagino H, Teshima R. Correlation between bone mineral density and intervertebral disk degeneration in pre- and postmenopausal women. Journal of Bone and Mineral Metabolism. 2003;21(1):22-7.

120. Neubert A, Fripp J, Engstrom C, Walker D, Weber MA, Schwarz R, et al. Three-dimensional morphological and signal intensity features for detection of intervertebral disc degeneration from magnetic resonance images. Journal of the American Medical Informatics Association. 2013;20(6):1082-90.

121. Niinimaki J, Korkiakoski A, Ojala O, Karppinen J, Ruohonen J, Haapea M, et al. Association between visual degeneration of intervertebral discs and the apparent diffusion coefficient. Magnetic Resonance Imaging. 2009;27(5):641-7.

122. Ohtori S, Ito T, Yamashita M, Murata Y, Morinaga T, Hirayama J, et al. Evaluation of low back pain using the Japanese Orthopaedic Association Back Pain Evaluation Questionnaire for lumbar spinal disease in a multicenter study: Differences in scores based on age, sex, and type of disease. Journal of Orthopaedic Science. 2010;15(1):86-91.

123. Olah M, Molnar L, Dobai J, Olah C, Feher J, Bender T. The effects of weightbath traction hydrotherapy as a component of complex physical therapy in disorders of the cervical and lumbar spine: A controlled pilot study with follow-up. Rheumatology International. 2008;28(8):749-56.

124. Omair A, Holden M, Lie BA, Reikeras O, Brox JI. Treatment outcome of chronic low back pain and radiographic lumbar disc degeneration are associated with inflammatory and matrix degrading gene variants: a prospective genetic association study. BMC Musculoskeletal Disorders. 2013;14:105.

125. Ong A, Anderson J, Roche J. A pilot study of the prevalence of lumbar disc degeneration in elite athletes with lower back pain at the Sydney 2000 Olympic Games. British Journal of Sports Medicine. 2003;37(3):263-6.

126. Oprea M, Popa I, Cimpean AM, Raica M, Poenaru DV. Microscopic assessment of degenerated intervertebral disc: Clinical implications and possible therapeutic challenge. In Vivo. 2015;29(1):95-102.

127. Oprea M, Popa I, Cimpean AM, Raica M, Poenaru DV. Microscopic assessment of degenerated intervertebral disc: clinical implications and possible therapeutic challenge. In Vivo. 2015;29(1):95-102.

128. Osti OL, Fraser RD. MRI and discography of annular tears and intervertebral disc degeneration: A prospective clinical comparison. Journal of Bone and Joint Surgery - Series B. 1992;74(3):431-5.

129. Ouyang L, Jia QX, Xiao YH, Ke LS, He P. Magnetic resonance imaging: a valuable method for diagnosing chronic lumbago caused by lumbar muscle strain and monitoring healing process. Chinese Medical Journal. 2013;126(13):2465-71.

130. Paajanen H, Erkintalo M, Parkkola R, Salminen J, Kormano M. Age-dependent correlation of low-back pain and lumbar disc degeneration. Archives of Orthopaedic and Trauma Surgery. 1997;116(1-2):106-7.

131. Pappou IP, Cammisa FP, Jr., Girardi FP. Correlation of end plate shape on MRI and disc degeneration in surgically treated patients with degenerative disc disease and herniated nucleus pulposus. Spine Journal: Official Journal of the North American Spine Society. 2007;7(1):32-8.

132. Park CK, Ryu KS, Jee WH. Degenerative changes of discs and facet joints in lumbar total disc replacement using ProDisc II: Minimum two-year follow-up. Spine. 2008;33(16):1755-61.

133. Park JB, Chang H, Kim KW, Park SJ. Facet tropism: A comparison between far lateral and posterolateral lumbar disc herniations. Spine. 2001;26(6):677-9.

134. Parkkola R, Rytokoski U, Kormano M. Magnetic resonance imaging of the discs and trunk muscles in patients with chronic low back pain and healthy control subjects. Spine. 1993;18(7):830-6.

135. Perez-Cruet MJ, Hussain NS, White GZ, Begun EM, Collins RA, Fahim DK, et al. Quality-of-life outcomes with minimally invasive transforaminal lumbar interbody fusion based on long-term analysis of 304 consecutive patients. Spine. 2014;39(3):E191-8.

136. Pfirrmann CWA, Metzdorf A, Elfering A, Hodler J, Boos N. Effect of aging and degeneraton on disc volume and shape: A quantitative study in asymptomatic volunteers. Journal of Orthopaedic Research. 2006;24(5):1086-94.

137. Pfirrmann CWA, Metzdorf A, Zanetti M, Hodler J, Boos N. Magnetic resonance classification of lumbar intervertebral disc degeneration. Spine. 2001;26(17):1873-8.

138. Pirotte B, Gabrovsky N, Massager N, Levivier M, David P, Brotchi J. Synovial cysts of the lumbar spine: surgery-related results and outcome. Journal of Neurosurgery. 2003;99(1 Suppl):14-9.

139. Poureisa M, Daghighi MH, Mesbahi S, Hagigi A, Fouladi DF. End plate disproportion and degenerative disc disease: a case-control study. Asian Spine Journal. 2014;8(4):405-11.

140. Powell MC, Wilson M, Szypryt P. Prevalence of lumbar disc degeneration observed by magnetic resonance in symptomless women. Lancet. 1986;2(8520):1366-7.

141. Putzier M, Hoff E, Tohtz S, Gross C, Perka C, Strube P. Dynamic stabilization adjacent to single-level fusion: part II. No clinical benefit for asymptomatic, initially degenerated adjacent segments after 6 years follow-up. European Spine Journal. 2010;19(12):2181-9.

142. Quinlan JF, Duke D, Eustace S. Bertolotti's syndrome. Journal of Bone and Joint Surgery - Series B. 2006;88(9):1183-6.

143. Quiroz-Moreno R, Lezama-Suarez G, Gomez-Jimenez C. Disc alterations of lumbar spine on magnetic resonance images in asymptomatic workers. [Spanish]. Revista medica del Instituto Mexicano del Seguro Social. 2008;46(2):185-90.

144. Radziszewski KR. Analysis of CT and MRI investigations of patients with lumbar discopathy treated consentatively or surgically. Polish Journal of Radiology. 2007;72(4):50-6.

145. Rajasekaran S, Babu JN, Arun R, Armstrong BR, Shetty AP, Murugan S. ISSLS prize winner: A study of diffusion in human lumbar discs: a serial magnetic resonance imaging study documenting the influence of the endplate on diffusion in normal and degenerate discs. Spine. 2004;29(23):2654-67.

146. Rajasekaran S, Bajaj N, Tubaki V, Kanna RM, Shetty AP. ISSLS Prize winner: The anatomy of failure in lumbar disc herniation: an in vivo, multimodal, prospective study of 181 subjects. Spine. 2013;38(17):1491-500.

147. Ramadorai UE, Hire JM, DeVine JG. Magnetic resonance imaging of the cervical, thoracic, and lumbar spine in children: spinal incidental findings in pediatric patients. Global Spine Journal. 2014;4(4):223-8.

148. Ranson CA, Burnett AF, Kerslake RW. Injuries to the lower back in elite fast bowlers: acute stress changes on MRI predict stress fracture. Journal of Bone & Joint Surgery - British Volume. 2010;92(12):1664-8.

149. Rapala A, Rapala K, Stanislaw L. Nonsymptomatic changes in magnetic resonansce imaging of spine among airforce candidates. [Polish]. Chirurgia narzadow ruchu i ortopedia polska. 2005;70(6):411-4.

150. Raty HP, Battie MC, Videman T, Sarna S. Lumbar mobility in former elite male weight-lifters, soccer players, long-distance runners and shooters. Clinical Biomechanics. 1997;12(5):325-30.

151. Regev GJ, Chen L, Dhawan M, Lee YP, Garfin SR, Kim CW. Morphometric analysis of the ventral nerve roots and retroperitoneal vessels with respect to the minimally invasive lateral approach in normal and deformed spines. Spine. 2009;34(12):1330-5.

152. Ricci C, Cova M, Kang YS, Yang A, Rahmouni A, Scott Jr WW, et al. Normal age-related patterns of cellular and fatty bone marrow distribution in the axial skeleton: MR imaging study. Radiology. 1990;177(1):83-8.

153. Richards BS, Sucato DJ, Johnston CE, Diab M, Sarwark JF, Lenke LG, et al. Right thoracic curves in presumed adolescent idiopathic scoliosis: which clinical and radiographic findings correlate with a preoperative abnormal magnetic resonance image? Spine. 2010;35(20):1855-60.

154. Riesenburger RI, Safain MG, Ogbuji R, Hayes J, Hwang SW. A novel classification system of lumbar disc degeneration. Journal of Clinical Neuroscience. 2015;22(2):346-51.

155. Salk I, Sabanciogullari V, Cetin A, Balaban H, Kelkit S. Value of findings of diffusion-weighted magnetic resonance imaging in patients with lumbar disc degeneration. Pakistan Journal of Medical Sciences. 2011;27(2):261-4.

156. Salminen JJ, Erkintalo M, Laine M, Pentti J, Battie MC. Low back pain in the young: A prospective three-year follow-up study of subjects with and without low back pain. Spine. 1995;20(19):2101-8.

157. Salminen JJ, Erkintalo MO, Pentti J, Oksanen A, Kormano MJ. Recurrent low back pain and early disc degeneration in the young. Spine. 1999;24(13):1316-21.

158. Salminen JJ, Erkintalo-Tertti MO, Paajanen HE. Magnetic resonance imaging findings of lumbar spine in the young: correlation with leisure time physical activity, spinal mobility, and trunk muscle strength in 15-year-old pupils with or without low-back pain. Journal of Spinal Disorders. 1993;6(5):386-91.

159. Salo S, Paajanen H, Alanen A. Disc degeneration of pediatric patients in lumbar MRI. Pediatric Radiology. 1995;25(3):186-9.

160. Sambrook PN, MacGregor AJ, Spector TD. Genetic influences on cervical and lumbar disc degeneration: A magnetic resonance imaging study in twins. Arthritis and Rheumatism. 1999;42(2):366-72.

161. Savage RA, Whitehouse GH, Roberts N. The relationship between the magnetic resonance imaging appearance of the lumbar spine and low back pain, age and occupation in males. European Spine Journal. 1997;6(2):106-14.

162. Saywell WR, Crock HV, England JP, Steiner RE. Demonstration of vertebral body end plate veins by magnetic resonance imaging. British Journal of Radiology. 1989;62(735):290-2.

163. Schneiderman G, Flannigan B, Kingston S. Magnetic resonance imaging in the diagnosis of disc degeneration: Correlation with discography. Spine. 1987;12(3):276-81.

164. Scuderi GJ, Brusovanik GV, Golish SR, DeMeo R, Hyde J, Hallab N, et al. A critical evaluation of discography in patients with lumbar intervertebral disc disease. Spine Journal. 2008;8(4):624-9.

165. Shambrook J, McNee P, Harris EC, Kim M, Sampson M, Palmer KT, et al. Clinical presentation of low back pain and association with risk factors according to findings on magnetic resonance imaging. Pain. 2011;152(7):1659-65.

166. Shang XP, Sun XC, Wang YX, Ju BB. Association of BCL-2 polymorphism with the presence and severity of lumbar disc degeneration in the Chinese Han population. Clinical Laboratory. 2012;58(3-4):261-6.

167. Sharma A, Pilgram T, Wippold IFJ. Association between annular tears and disk degeneration: A longitudinal study. American Journal of Neuroradiology. 2009;30(3):500-6.

168. Siepe CJ, Heider F, Haas E, Hitzl W, Szeimies U, Stabler A, et al. Influence of lumbar intervertebral disc degeneration on the outcome of total lumbar disc replacement: a prospective clinical, histological, X-ray and MRI investigation. European Spine Journal. 2012;21(11):2287-99.

169. Siepe CJ, Zelenkov P, Sauri-Barraza JC, Szeimies U, Grubinger T, Tepass A, et al. The fate of facet joint and adjacent level disc degeneration following total lumbar disc replacement: a prospective clinical, X-ray, and magnetic resonance imaging investigation. Spine. 2010;35(22):1991-2003.

170. Smith FW. Upright MRI in the seated position increases insight into degenerative disc disease. Clinical MRI. 2006;15(3):8-12.

171. Smith JS, Sidhu G, Bode K, Gendelberg D, Maltenfort M, Ibrahimi D, et al. Operative and nonoperative treatment approaches for lumbar degenerative disc disease have similar long-term clinical outcomes among patients with positive discography. World Neurosurgery. 2014;82(5):872-8.

172. Solovieva S, Kouhia S, Leino-Arjas P, Ala-Kokko L, Luoma K, Raininko R, et al. Interleukin 1 polymorphisms and intervertebral disc degeneration. Epidemiology. 2004;15(5):626-33.

173. Solovieva S, Lohiniva J, Leino-Arjas P, Raininko R, Luoma K, Ala-Kokko L, et al. COL9A3 gene polymorphism and obesity in intervertebral disc degeneration of the lumbar spine: Evidence of gene-environment interaction. Spine. 2002;27(23):2691-6.

174. Solovieva S, Lohiniva J, Leino-Arjas P, Raininko R, Luoma K, Ala-Kokko L, et al. Intervertebral disc degeneration in relation to the COL9A3 and the IL-1beta gene polymorphisms. European Spine Journal. 2006;15(5):613-9.

175. Solovieva S, Noponen N, Mannikko M, Leino-Arjas P, Luoma K, Raininko R, et al. Association between the aggrecan gene variable number of tandem repeats polymorphism and intervertebral disc degeneration. Spine. 2007;32(16):1700-5.

176. Song YQ, Ho DWH, Karppinen J, Kao PYP, Fan BJ, Luk KDK, et al. Association between promoter - 1607 polymorphism of MMP1 and lumbar disc disease in Southern Chinese. BMC Medical Genetics. 2008;9(38).

177. Stelzeneder D, Welsch GH, Kovacs BK, Goed S, Paternostro-Sluga T, Vlychou M, et al. Quantitative T2 evaluation at 3.0T compared to morphological grading of the lumbar intervertebral disc: a standardized evaluation approach in patients with low back pain. European Journal of Radiology. 2012;81(2):324-30.

178. Styczynski T, Pysklo B, Gasik R. The effect of the grade of degenerative changes in the spine on the outcomes of surgery for lumbar discopathy with a radicular syndrome. Ortopedia Traumatologia Rehabilitacja. 2007;9(6):591-8.

179. Sun ZM, Miao L, Zhang YG, Ming L. Association between the -1562 C/T polymorphism of matrix metalloproteinase-9 gene and lumbar disc disease in the young adult population in North China. Connective Tissue Research. 2009;50(3):181-5.

180. Taira G, Endo K, Ito K, Ichimaru K, Imakiire A, Miura Y. Diagnosis of lumbar disc herniation by three-dimensional MRI. Journal of Orthopaedic Science. 1998;3(1):18-26.

181. Takahashi K, Miyazaki T, Ohnari H, Takino T, Tomita K. Schmorl's nodes and low-back pain. Analysis of magnetic resonance imaging findings in symptomatic and asymptomatic individuals. European Spine Journal. 1995;4(1):56-9.

182. Takahashi M, Haro H, Wakabayashi Y, Kawa-uchi T, Komori H, Shinomiya K. The association of degeneration of the intervertebral disc with 5a/6a polymorphism in the promoter of the human matrix metalloproteinase-3 gene. Journal of Bone and Joint Surgery - Series B. 2001;83(4):491-5.

183. Takashima H, Takebayashi T, Yoshimoto M, Terashima Y, Tsuda H, Ida K, et al. Correlation between T2 relaxation time and intervertebral disk degeneration. Skeletal Radiology. 2012;41(2):163-7.

184. Takatalo J, Karppinen J, Niinimaki J, Taimela S, Nayha S, Jarvelin MR, et al. Prevalence of degenerative imaging findings in lumbar magnetic resonance imaging among young adults. Spine. 2009;34(16):1716-21.

185. Takatalo J, Karppinen J, Niinimaki J, Taimela S, Nayha S, Mutanen P, et al. Does lumbar disc degeneration on magnetic resonance imaging associate with low back symptom severity in young Finnish adults? Spine. 2011;36(25):2180-9.

186. Takatalo J, Karppinen J, Taimela S, Niinimaki J, Laitinen J, Blanco Sequeiros R, et al. Body mass index is associated with lumbar disc degeneration in young Finnish males: subsample of Northern Finland birth cohort study 1986. BMC Musculoskeletal Disorders. 2013;14:87.

187. Takatalo J, Karppinen J, Taimela S, Niinimaki J, Laitinen J, Sequeiros RB, et al. Association of Abdominal Obesity with Lumbar Disc Degeneration - A Magnetic Resonance Imaging Study. PLoS ONE. 2013;8(2).

188. Tan H, Zhao J, Jiang J, Ren Y. Association of the polymorphism of DR4 with the risk and severity of lumbar disc degeneration in the Chinese Han population. Scandinavian Journal of Clinical & Laboratory Investigation. 2012;72(7):576-9.

189. Teraguchi M, Yoshimura N, Hashizume H, Muraki S, Yamada H, Minamide A, et al. Prevalence and distribution of intervertebral disc degeneration over the entire spine in a population-based cohort: the Wakayama Spine Study. Osteoarthritis & Cartilage. 2014;22(1):104-10.

190. Tertti MO, Salminen JJ, Paajanen HE, Terho PH, Kormano MJ. Low-back pain and disk degeneration in children: a case-control MR imaging study. Radiology. 1991;180(2):503-7.

191. Tibiletti M, Galbusera F, Ciavarro C, Brayda-Bruno M. Is the transport of a gadolinium-based contrast agent decreased in a degenerated or aged disc? A post contrast MRI study. PLoS ONE [Electronic Resource]. 2013;8(10):e76697.

192. Truszczynska A, Brychcy A, Rapala K, Walczak P, Truszczynski O, Tarnowski A. Interrater and intrarater reliability of lumbar spine magnetic resonance assessment of military aircraft pilot candidates. Ortopedia Traumatologia Rehabilitacja. 2011;13(4):343-51.

193. Videman T, Battie MC, Gibbons LE, Gill K. Aging changes in lumbar discs and vertebrae and their interaction: a 15-year follow-up study. Spine Journal: Official Journal of the North American Spine Society. 2014;14(3):469-78.

194. Videman T, Battie MC, Gibbons LE, Manninen H, Gill K, Fisher LD, et al. Lifetime exercise and disk degeneration: An MRI study of monozygotic twins. Medicine and Science in Sports and Exercise. 1997;29(10):1350-6.

195. Videman T, Battié MC, Gibbons LE, Maravilla K, Manninen H, Kaprio J. Associations between back pain history and lumbar MRI findings. Spine. 2003;28(6):582-8.

196. Videman T, Battie MC, Gill K, Manninen H, Gibbons LE, Fisher LD. Magnetic resonance imaging findings and their relationships in the thoracic and lumbar spine. Insights into the etiopathogenesis of spinal degeneration. Spine. 1995;20(8):928-35.

197. Videman T, Battie MC, Parent E, Gibbons LE, Vainio P, Kaprio J. Progression and determinants of quantitative magnetic resonance imaging measures of lumbar disc degeneration: A five-year follow-up of adult male monozygotic twins. Spine. 2008;33(13):1484-90.

198. Videman T, Battie MC, Ripatti S, Gill K, Manninen H, Kaprio J. Determinants of the progression in lumbar degeneration: a 5-year follow-up study of adult male monozygotic twins. Spine. 2006;31(6):671-8.

199. Videman T, Gibbons LE, Battie MC, Maravilla K, Vanninen E, Leppavuori J, et al. The relative roles of intragenic polymorphisms of the vitamin d receptor gene in lumbar spine degeneration and bone density. Spine. 2001;26(3):E7-E12.

200. Videman T, Gibbons LE, Kaprio J, Battie MC. Challenging the cumulative injury model: positive effects of greater body mass on disc degeneration. Spine Journal: Official Journal of the North American Spine Society. 2010;10(1):26-31.

201. Videman T, Leppavuori J, Kaprio J, Battie MC, Gibbons LE, Peltonen L, et al. Intragenic polymorphisms of the vitamin D receptor gene associated with intervertebral disc degeneration. Spine. 1998;23(23):2477-85.

202. Videman T, Levalahti E, Battie MC. The effects of anthropometrics, lifting strength, and physical activities in disc degeneration. Spine. 2007;32(13):1406-13.

203. Videman T, Saarela J, Kaprio J, Nakki A, Levalahti E, Gill K, et al. Associations of 25 structural, degradative, and inflammatory candidate genes with lumbar disc desiccation, bulging, and height narrowing. Arthritis and Rheumatism. 2009;60(2):470-81.

204. Visuri T, Ulaska J, Eskelin M, Pulkkinen P. Narrowing of lumbar spinal canal predicts chronic low back pain more accurately than intervertebral disc degeneration: A magnetic resonance imaging study in young Finnish male conscripts. Military Medicine. 2005;170(11):926-30.

205. Wang YX, Griffith JF, Ma HT, Kwok AW, Leung JC, Yeung DK, et al. Relationship between gender, bone mineral density, and disc degeneration in the lumbar spine: a study in elderly subjects using an eight-level MRI-based disc degeneration grading system. Osteoporosis International. 2011;22(1):91-6.

206. Wang YX, Kwok AW, Griffith JF, Leung JC, Ma HT, Ahuja AT, et al. Relationship between hip bone mineral density and lumbar disc degeneration: a study in elderly subjects using an eight-level MRI-based disc degeneration grading system. Journal of Magnetic Resonance Imaging. 2011;33(4):916-20.

207. Wang YX, Zhao F, Griffith JF, Mok GS, Leung JC, Ahuja AT, et al. T1rho and T2 relaxation times for lumbar disc degeneration: an in vivo comparative study at 3.0-Tesla MRI. European Radiology. 2013;23(1):228-34.

208. Waris E, Eskelin M, Hermunen H, Kiviluoto O, Paajanen H. Disc degeneration in low back pain: A 17-year follow-up study using magnetic resonance imaging. Spine. 2007;32(6):681-4.

209. West W, West KP, Younger EN, Cornwall D. Degenerative disc disease of the lumbar spine on MRI. West Indian Medical Journal. 2010;59(2):192-5.

210. Willems PC, Elmans L, Anderson PG, Van Der Schaaf DB, De Kleuver M. Provocative discography and lumbar fusion: Is preoperative assessment of adjacent discs useful? Spine. 2007;32(10):1094-9.

211. Williams FM, Manek NJ, Sambrook PN, Spector TD, Macgregor AJ. Schmorl's nodes: common, highly heritable, and related to lumbar disc disease. Arthritis & Rheumatism. 2007;57(5):855-60.

212. Williams FM, Popham M, Sambrook PN, Jones AF, Spector TD, MacGregor AJ. Progression of lumbar disc degeneration over a decade: a heritability study. Annals of the Rheumatic Diseases. 2011;70(7):1203-7.

213. Wu Z, Yang B, Pan S, Chen Z. MRI evaluation of bone marrow of normal lumbar vertebra in the Chinese: normal patterns and preliminary quantitative study. Chinese Medical Journal. 1999;112(7):646-8.

214. Yang X, Kong Q, Song Y, Liu L, Zeng J, Xing R. The characteristics of spinopelvic sagittal alignment in patients with lumbar disc degenerative diseases. European Spine Journal. 2014;23(3):569-75.

215. Ye W, Huang DS, Chen WJ, Li CH, Peng Y, Liang AJ, et al. Association of 86 bp variable number tandem repeat polymorphism of interleukin-1 receptor antagonist gene with lumbar disc disease. [Chinese]. Nan fang yi ke da xue xue bao = Journal of Southern Medical University. 2007;27(10):1485-8.

216. Yin R, Lord EL, Cohen JR, Buser Z, Lao L, Zhong G, et al. Distribution of Schmorl nodes in the lumbar spine and their relationship with lumbar disk degeneration and range of motion. Spine. 2014;40(1):E49-E53.

217. Zagra A, Minoia L, Archetti M, Corriero AS, Ricci K, Teli M, et al. Prospective study of a new dynamic stabilisation system in the treatment of degenerative discopathy and instability of the lumbar spine. European Spine Journal. 2012;21 Suppl 1:S83-9.

218. Zawilla NH, Darweesh H, Mansour N, Helal S, Taha FM, Awadallah M, et al. Matrix metalloproteinase-3, vitamin D receptor gene polymorphisms, and occupational risk factors in lumbar disc degeneration. Journal of Occupational Rehabilitation. 2014;24(2):370-81.

219. Zbigniew S, Kamila KK. An analysis of pain symptoms in patients with chronic spinal syndrome under treatment in health resorts. [Polish]. Fizjoterapia Polska. 2003;3(1):38-47.

220. Zhang W, Ma X, Wang Y, Zhao J, Zhang X, Gao Y, et al. Assessment of apparent diffusion coefficient in lumbar intervertebral disc degeneration. European Spine Journal. 2014;23(9):1830-6.

221. Zhang Y, Gu Z, Qiu G. Association of the polymorphism of MMP2 with the risk and severity of lumbar disc degeneration in the Chinese Han population. European Review for Medical & Pharmacological Sciences. 2013;17(13):1830-4.

222. Zhang ZM, Zhao L, Qu DB, Jin DD. Artificial nucleus replacement: surgical and clinical experience. Orthopaedic Audio-Synopsis Continuing Medical Education [Sound Recording]. 2009;1(1):52-7.

223. Zhao B, Wang K, Zhao J, Luo Y. Serum calcium concentration as an indicator of intervertebral disk degeneration prognosis. Biological Trace Element Research. 2013;154(3):333-7.

224. Zobel BB, Vadala G, Del Vescovo R, Battisti S, Martina FM, Stellato L, et al. T1p magnetic resonance imaging quantification of early lumbar intervertebral disc degeneration in healthy young adults. Spine. 2012;37(14):1224-30.

225. Zook J, Djurasovic M, Crawford C, 3rd, Bratcher K, Glassman S, Carreon L. Inter- and intraobserver reliability in radiographic assessment of degenerative disk disease. Orthopedics. 2011;34(4).

226. Zou J, Yang H, Miyazaki M, Morishita Y, Wei F, McGovern S, et al. Dynamic bulging of intervertebral discs in the degenerative lumbar spine. Spine. 2009;34(23):2545-50.

## Not all lumbar levels

1. Arbanas J, Pavlovic I, Marijancic V, Vlahovic H, Starcevic-Klasan G, Peharec S, et al. MRI features of the psoas major muscle in patients with low back pain. European Spine Journal. 2013;22(9):1965-71.

2. Becker GT, Willburger RE, Liphofer J, Koester O, Schmid C. Distribution of MRI signal alterations of the cartilage endplate in pre-operated patients with special focus on recurrent lumbar disc herniation. [German]. RoFo Fortschritte auf dem Gebiet der Rontgenstrahlen und der Bildgebenden Verfahren. 2006;178(1):46-54.

3. Berg L, Gjertsen O, Hellum C, Neckelmann G, Johnsen LG, Eide GE, et al. Reliability of change in lumbar MRI findings over time in patients with and without disc prosthesis--comparing two different image evaluation methods. Skeletal Radiology. 2012;41(12):1547-57.

4. Berg L, Hellum C, Gjertsen O, Neckelmann G, Johnsen LG, Storheim K, et al. Do more MRI findings imply worse disability or more intense low back pain? A cross-sectional study of candidates for lumbar disc prosthesis. Skeletal Radiology. 2013;42(11):1593-602.

5. Berg L, Neckelmann G, Gjertsen O, Hellum C, Johnsen LG, Eide GE, et al. Reliability of MRI findings in candidates for lumbar disc prosthesis. Neuroradiology. 2012;54(7):699-707.

6. Blondel B, Tropiano P, Gaudart J, Huang RC, Marnay T. Clinical results of lumbar total disc arthroplasty in accordance with Modic signs, with a 2-year-minimum follow-up. Spine. 2011;36(26):2309-15.

7. Cevei M, Rosca E, Liviu L, Mutiu G, Stoicanescu D, Vasile L. Imagistic and histopathologic concordances in degenerative lesions of intervertebral disks. Romanian Journal of Morphology & Embryology. 2011;52(1 Suppl):327-32.

8. Chen Z, Peng B, Li D, Pang X, Yang H. Minimum 5-year follow-up study on the effects of the Wallis dynamic stabilization system in the treatment of lumbar degenerative disease. Chinese Medical Journal. 2014;127(20):3587-91.

9. Chin KR, Tomlinson DT, Auerbach JD, Shatsky JB, Deirmengian CA. Success of lumbar microdiscectomy in patients with modic changes and low-back pain: a prospective pilot study. Journal of Spinal Disorders & Techniques. 2008;21(2):139-44.

10. Corniola MV, Stienen MN, Joswig H, Smoll NR, Schaller K, Hildebrandt G, et al. Correlation of pain, functional impairment, and health-related quality of life with radiological grading scales of lumbar degenerative disc disease. Acta Neurochirurgica. 2016;158(3):499-505.

11. Djurasovic M, Carreon LY, Crawford CH, 3rd, Zook JD, Bratcher KR, Glassman SD. The influence of preoperative MRI findings on lumbar fusion clinical outcomes. European Spine Journal. 2012;21(8):1616-23.

12. el Barzouhi A, Vleggeert-Lankamp CL, van der Kallen BF, Lycklama a Nijeholt GJ, van den Hout WB, Koes BW, et al. Back pain's association with vertebral end-plate signal changes in sciatica. Spine Journal: Official Journal of the North American Spine Society. 2014;14(2):225-33.

13. El Barzouhi A, Vleggeert-Lankamp CLAM, Van Der Kallen BF, Lycklama ANGJ, Van Den Hout WB, Koes BW, et al. Back pain's association with vertebral end-plate signal changes in sciatica. Spine Journal. 2014;14(2):225-33.

14. Gornet MF, Schranck F, Wharton ND, Beall DP, Jones E, Myers ME, et al. Optimizing success with lumbar disc arthroplasty. European Spine Journal. 2014;23(10):2127-35.

15. Hasegawa K, Shimoda H, Kitahara K, Sasaki K, Homma T. What are the reliable radiological indicators of lumbar segmental instability? Journal of Bone & Joint Surgery - British Volume. 2011;93(5):650-7.

16. He X, Liang A, Gao W, Peng Y, Zhang L, Liang G, et al. The relationship between concave angle of vertebral endplate and lumbar intervertebral disc degeneration. Spine. 2012;37(17):E1068-73.

17. Hellum C, Berg L, Gjertsen O, Johnsen LG, Neckelmann G, Storheim K, et al. Adjacent level degeneration and facet arthropathy after disc prosthesis surgery or rehabilitation in patients with chronic low back pain and degenerative disc: Second report of a randomized study. Spine. 2012;37(25):2063-73.

18. Hernandez Martinez A, Pellise Urquiza F, Becerra Fontal JA, Rovira A, Bago Granell J, Villanueva Leal C. Signs predictive of lumbar disk pain: Correlation between MRI and discography. [Spanish]. Revista de Ortopedia y Traumatologia. 2002;46(6):528-33.

19. Hrabalek L, Reskova I, Buil J, Vaverka M, Houdek M. The use of Titan and PEEK implants in stand-alone ALIF surgery for degenerative disease of the Lumbosacral spine - A prospective study. [Czech]. Ceska a Slovenska Neurologie a Neurochirurgie. 2009;72(1):38-44.

20. Jerosch J, Castro WHM, Halm H, Meyer M, Assheuer J. Long term changes after chemonucleolysis in the MRI. [German]. Zeitschrift fur Orthopadie und Ihre Grenzgebiete. 1994;132(1):2-8.

21. Jones A, Clarke A, Freeman BJ, Lam KS, Grevitt MP. The Modic classification: inter- and intraobserver error in clinical practice. Spine. 2005;30(16):1867-9.

22. Keller A, Boyle E, Skog TA, Cassidy JD, Bautz-Holter E. Are Modic changes prognostic for recovery in a cohort of patients with non-specific low back pain? European Spine Journal. 2012;21(3):418-24.

23. Keller A, Boyle E, Skog TA, David Cassidy J, Bautz-Holter E. Are Modic changes prognostic for recovery in a cohort of patients with non-specific low back pain? European Spine Journal. 2012;21(3):418-24.

24. Kuisma M, Karppinen J, Haapea M, Lammentausta E, Niinimaki J, Tervonen O. Modic changes in vertebral endplates: a comparison of MR imaging and multislice CT. Skeletal Radiology. 2009;38(2):141-7.

25. Liphofer JP, Theodoridis T, Becker GT, Koester O, Schmid G. (Modic) signal alterations of vertebral endplates and their correlation to a minimally invasive treatment of lumbar disc herniation using epidural injections. [German]. RoFo Fortschritte auf dem Gebiet der Rontgenstrahlen und der Bildgebenden Verfahren. 2006;178(11):1105-14.

26. Liquois F, Tournier C, Xu BS, Le Huec JC. Anterior retroperitoneal L5-S1 arthrodesis and fixation with cage and plate. Prospective study in 40 cases with a follow-up of one to 2.2 year (mean 1.5 year). [French]. Revue de Chirurgie Orthopedique et Reparatrice de l'Appareil Moteur. 2008;94(3):273-81.

27. Liu HY, Zhou J, Wang B, Wang HM, Jin ZH, Zhu ZG, et al. Comparison of topping-off and posterior lumbar interbody fusion surgery in lumbar degenerative disease: A retrospective study. Chinese Medical Journal. 2012;125(22):3942-6.

28. Lurie JD, Moses RA, Tosteson AN, Tosteson TD, Carragee EJ, Carrino JA, et al. Magnetic resonance imaging predictors of surgical outcome in patients with lumbar intervertebral disc herniation. Spine. 2013;38(14):1216-25.

29. Masaryk TJ, Boumphrey F, Modic MT, Tamborrello C, Ross JS, Brown MD. Effects of chemonucleolysis demonstrated by MR imaging. Journal of Computer Assisted Tomography. 1986;10(6):917-23.

30. Mattei TA, Rehman AA, Teles AR, Aldag JC, Dinh DH, McCall TD. The 'Lumbar Fusion Outcome Score' (LUFOS): a new practical and surgically oriented grading system for preoperative prediction of surgical outcomes after lumbar spinal fusion in patients with degenerative disc disease and refractory chronic axial low back pain. Neurosurgical Review. 2016:11.

31. Putzier M, Schneider SV, Funk JF, Tohtz SW, Perka C. The surgical treatment of the lumbar disc prolapse: nucleotomy with additional transpedicular dynamic stabilization versus nucleotomy alone. Spine. 2005;30(5):E109-14.

32. Raininko R, Manninen H, Battie MC, Gibbons LE, Gill K, Fisher LD. Observer variability in the assessment of disc degeneration on magnetic resonance images of the lumbar and thoracic spine. Spine. 1995;20(9):1029-35.

33. Robertson WD, Jarvik JG, Tsuruda JS, Koepsell TD, Maravilla KR. The comparison of a rapid screening MR protocol with a conventional MR protocol for lumbar spondylosis. AJR American Journal of Roentgenology. 1996;166(4):909-16.

34. Ross JS, Zepp R, Modic MT. The postoperative lumbar spine: Enhanced MR evaluation of the intervertebral disk. American Journal of Neuroradiology. 1996;17(2):323-31.

35. Shan Z, Fan S, Xie Q, Suyou L, Liu J, Wang C, et al. Spontaneous resorption of lumbar disc herniation is less likely when modic changes are present. Spine. 2014;39(9):736-44.

36. Shen M, Razi A, Lurie JD, Hanscom B, Weinstein J. Retrolisthesis and lumbar disc herniation: a preoperative assessment of patient function. Spine Journal: Official Journal of the North American Spine Society. 2007;7(4):406-13.

37. Sorile A, Moholdt V, Kvistad KA, Nygaard OP, Ingebrigtsen T, Iversen T, et al. Modic type i changes and recovery of back pain after lumbar microdiscectomy. European Spine Journal. 2012;21(11):2252-8.

38. Stabler A, Bellan M, Weiss M, Gartner C, Brossmann J, Reiser MF. MR imaging of enhancing intraosseous disk herniation (Schmorl's nodes). AJR American Journal of Roentgenology. 1997;168(4):933-8.

39. Stabler A, Weiss M, Scheidler J, Krodel A, Seiderer M, Reiser M. Degenerative disk vascularization on MRI: correlation with clinical and histopathologic findings. Skeletal Radiology. 1996;25(2):119-26.

40. Toyone T, Takahashi K, Kitahara H, Yamagata M, Murakami M, Moriya H. Vertebral bone-marrow changes in degenerative lumbar disc disease. An MRI study of 74 patients with low back pain. Journal of Bone & Joint Surgery - British Volume. 1994;76(5):757-64.

41. Yu LP, Qian WW, Yin GY, Ren YX, Hu ZY. MRI assessment of lumbar intervertebral disc degeneration with lumbar degenerative disease using the Pfirrmann grading systems. PLoS ONE [Electronic Resource]. 2012;7(12):e48074.

## Only MC patients

1. Cao P, Jiang L, Zhuang C, Yang Y, Zhang Z, Chen W, et al. Intradiscal injection therapy for degenerative chronic discogenic low back pain with end plate Modic changes. Spine Journal: Official Journal of the North American Spine Society. 2011;11(2):100-6.

2. Chataigner H, Onimus M, Polette A. Criteria for surgery in degenerative lumbar disc disease. [French]. Revue de Chirurgie Orthopedique et Reparatrice de l'Appareil Moteur. 1998;84(7):583-9.

3. Fayad F, Lefevre-Colau MM, Drape JL, Feydy A, Chemla N, Quintero N, et al. Reliability of a modified Modic classification of bone marrow changes in lumbar spine MRI. Joint, Bone, Spine: Revue du Rhumatisme. 2009;76(3):286-9.

4. Fayad F, Lefevre-Colau MM, Rannou F, Quintero N, Nys A, Mace Y, et al. Relation of inflammatory modic changes to intradiscal steroid injection outcome in chronic low back pain. European Spine Journal. 2007;16(7):925-31.

5. Hutton MJ, Bayer JH, Powell JM. Modic vertebral body changes: the natural history as assessed by consecutive magnetic resonance imaging. Spine. 2011;36(26):2304-7.

6. Jensen RK, Leboeuf-Yde C, Wedderkopp N, Sorensen JS, Jensen TS, Manniche C. Is the development of Modic changes associated with clinical symptoms? A 14-month cohort study with MRI. European Spine Journal. 2012;21(11):2271-9.

7. Kiyotaka Y, Toshio N, Takuro S, Toshikatsu K, Teruaki O, Haruhiko T, et al. Targeted Therapy for Low Back Pain in Elderly Degenerative Lumbar Scoliosis: A Cohort Study. Spine (03622436). 2016;41(10):872-9 8p.

8. Lusins JO, Cicoria AD, Goldsmith SJ. SPECT and lumbar MRI in back pain with emphasis on changes in end plates in association with disc degeneration. Journal of Neuroimaging. 1998;8(2):78-82.

9. Mitra D, Cassar-Pullicino VN, McCall IW. Longitudinal study of vertebral type-1 end-plate changes on MR of the lumbar spine. European Radiology. 2004;14(9):1574-81.

10. Stumpe KD, Zanetti M, Weishaupt D, Hodler J, Boos N, Von Schulthess GK. FDG positron emission tomography for differentiation of degenerative and infectious endplate abnormalities in the lumbar spine detected on MR imaging. AJR American Journal of Roentgenology. 2002;179(5):1151-7.

11. Wu HT, Morrison WB, Schweitzer ME. Edematous Schmorl's nodes on thoracolumbar MR imaging: characteristic patterns and changes over time. Skeletal Radiology. 2006;35(4):212-9.

## Specific LBP condition

1. Baykara RA, Bozgeyik Z, Akgul O, Ozgocmen S. Low back pain in patients with rheumatoid arthritis: clinical characteristics and impact of low back pain on functional ability and health related quality of life. Journal of Back & Musculoskeletal Rehabilitation. 2013;26(4):367-74.

2. Briot K, Durnez A, Paternotte S, Miceli-Richard C, Dougados M, Roux C. Bone oedema on MRI is highly associated with low bone mineral density in patients with early inflammatory back pain: results from the DESIR cohort. Annals of the Rheumatic Diseases. 2013;72(12):1914-9.

3. Buttermann GR, Mullin WJ. Pain and disability correlated with disc degeneration via magnetic resonance imaging in scoliosis patients. European Spine Journal. 2008;17(2):240-9.

4. Jeong HY, You JW, Sohn HM, Park SH. Radiologic evaluation of degeneration in isthmic and degenerative spondylolisthesis. Asian Spine Journal. 2013;7(1):25-33.

5. Nguyen C, Bendeddouche I, Sanchez K, Jousse M, Papelard A, Feydy A, et al. Assessment of ankylosing spondylitis criteria in patients with chronic low back pain and vertebral endplate Modic I signal changes. Journal of Rheumatology. 2010;37(11):2334-9.

6. Sharma A, Lancaster S, Bagade S, Hildebolt C. Early pattern of degenerative changes in individual components of intervertebral discs in stressed and nonstressed segments of lumbar spine: an in vivo magnetic resonance imaging study. Spine. 2014;39(13):1084-90.
