# Supplementary material for: Modic changes—Their associations with low back pain and activity limitation: A systematic literature review and meta-analysis
Source: PLoS One. 2018 Aug 1;13(8):e0200677. doi: 10.1371/journal.pone.0200677 (PMC6070210; doi:10.1371/journal.pone.0200677)
Supplement: S4 Appendix — (PDF) [file pone.0200677.s004.pdf]

PROSPERO International prospective register of systematic reviews

Modic changes: prevalence and association with low back pain - a systematic literature review and meta-analysis

Christofer Herlin, Tue Secher Jensen, Per Kjær, Ansgar Espeland, Jan Sture Skouen, Charlotte Leboeuf-Yde, Jaro Karppinen, Jaakko Niinimäki, Joan Solgaard Sørensen, Kjersti Storheim

Citation

Christofer Herlin, Tue Secher Jensen, Per Kjær, Ansgar Espeland, Jan Sture Skouen, Charlotte Leboeuf-Yde, Jaro Karppinen, Jaakko Niinimäki, Joan Solgaard Sørensen, Kjersti Storheim. Modic changes: prevalence and association with low back pain - a systematic literature review and meta-analysis. PROSPERO 2015:CRD42015017350
Available from http://www.crd.york.ac.uk/PROSPERO_REBRANDING/display_record.asp?ID=CRD42015017350

Review question(s)

What is the prevalence of modic changes in different age groups and in different study populations?

Is the presence and characteristics of modic changes associated with non-specific LBP and disability in the adolescent and adult populations?

Is any such association modified by other factors, e.g. age, sex, and other degenerative MRI findings?

Searches

MEDLINE, EMBASE and CINAHL electronic databases will be searched.

No restrictions will be applied.

Types of study to be included

Consecutive or random samples in population based or clinical studies, prospective, retrospective or cross-sectional. Excluding case reports, conferance papers, letters to the editor, in-vitro studies, ex-vivo studies, animal studies.

Condition or domain being studied

Vertebral Endplate Signal (Modic) Changes, defined by MRI of the lumbar spine. Prevalence and association with LBP.

Participants/ population

All populations, both general population and clinical. All ages. Participants with specific LBP conditions are excluded: Spondylitis, discitis or spondylodiscitis, spondylarthropathy (e.g. ankylosing spondylitis), fracture (including isthmic spondylolisthesis), spinal cord infarction, malignancy, hematological conditions, juvenile/idiopathic scoliosis. Also, participants treated with radiotherapy, or who have had spine surgery are excluded. Pre-intervention data will be included if available.

Intervention(s), exposure(s)

Vertebral Endplate Signal (Modic) Changes

Comparator(s)/ control

Not applicable

Outcome(s)

Primary outcomes

LBP, self-reported or by experimental measure (algometry, discography)

Secondary outcomes
LBP-related disability as measured by Oswestry Disability Index, Roland Morris Disability Questionnaire or similar.

Data extraction, (selection and coding)
Two investigators will independently inspect all retrieved titles, and exclude those articles that clearly do not fulfill the inclusion criteria and/or meet the exclusion criteria.

In cases of disagreement between the two investigators participating in the selection process, a third investigator will be consulted and the disagreement will be solved by discussion and further insights.

Two groups of investigators will handle each their objective in terms of data extraction. One group will handle the association studies (a-group), while the other handles the prevalence studies (p-group). The following process will be the same for both groups unless otherwise stated.

Two investigators will extract relevant data from each article, independently, using a form developed for this purpose. Disagreements will be resolved by discussion with a third participant of the group. If more input and/or insight is needed, the whole group will be consulted.

Data to be extracted from included articles:

* Year

* Country of data collection

* Study population

* Race of study population

* Sampling method

* Number of individuals

* Age

* Source of funding

* VESC/Modic definitions

* LBP definitions

* Disability definitions

* LBP data

* Disability data

Risk of bias (quality) assessment
Two independent investigators will assess the quality of the included articles, independently.

Quality/bias assessment for both prevalence and association studies will be based on the Quality Assessment of Diagnostic Accuracy Studies list (QUADAS 2) as recommended by the Cochrane Diagnostic Test Accuracy Working Group

Results from the quality assessment will be used for sensitivity analysis.

Strategy for data synthesis
Included articles will be presented in a table along with study characteristics

while excluded articles are referenced in an Appendix.

The prevalence rates of Modic changes will be reported in relation to the number

of individuals and/or in relation to the number of affected vertebral lumbar

levels used as the denominator.

Modic changes in relation to LBP and disability where association estimates are

reported (e g odds ratios) will be extracted from the articles.

Modic changes in relation to LBP and disability will be analyzed in two by two

tables, where odds ratios will be calculated. Confidence intervals which do not

include 1.0, will be considered statically significant.

In case of many included articles, the statistical analysis will be made as a meta-analysis

for studies that are homogeneic in relation to study population and clinical outcome, e.g. patients referred for lumbar surgery who had had provocative discography. Otherwise the different studies will be analyzed individually, and form the basis for a descriptive synthesis.

Analysis of subgroups or subsets
None planned

Contact details for further information
Dr Secher Jensen

Spine Center of Southern Denmark

Østre Hougvej 55

DK-5500 Middelfart

Denmark

Tue.Secher.Jensen@rsyd.dk

Organisational affiliation of the review
None

Review team
Mr Christofer Herlin,
Dr Tue Secher Jensen, 1) Clinical Associate Professor, Institute of Regional Health Research, University of Southern Denmark, Odense, Denmark 2) Senior Researcher, Nordic Institute of Chiropractic and Clinical Biomechanics, Odense, Denmark.
Dr Per Kjær, Department of Sports Science and Clinical Biomechanics, University of Southern Denmark, Odense, Denmark
Professor Ansgar Espeland, Department of Radiology, Haukeland University Hospital, Bergen and Department of Clinical Medicine, University of Bergen
Professor Jan Sture Skouen, Department of Global Public Health and Primary Care , University of Bergen, Norway. Department of Physical Medicine and Rehabilitation, Haukeland University Hospital, Bergen, Norway.
Professor Charlotte Leboeuf-Yde, The Spine Center of Southern Denmark, Hospital Lillebælt and Institute of Regional Health Research, University of Southern Denmark, Odense, Denmark

Professor Jaro Karppinen, 1) Center for Life Course Epidemiology and Systems Medicine, Faculty of Medicine, University of Oulu 2) Medical Research Center Oulu, Oulu University Hospital and University of Oulu, and 3) Finnish Institute of Occupational Health, Oulu, Finland
Dr Jaakko Niinimäki, Department of Diagnostic Radiology, Oulu University Hospital, Oulu, Finland
Dr Joan Solgaard Sørensen, External Lecturer, University of Southern Denmark, Odense, Denmark
Dr Kjersti Storheim, Communication and Research Unit for Musculoskeletal Disorders (FORMI), Oslo University Hospital and University of Oslo

Anticipated or actual start date
01 March 2015

Anticipated completion date
15 December 2015

Funding sources/sponsors
Funded by a grant from the Et Liv i Bevegelse (ELIB) foundation.

Conflicts of interest
None known

Language
English

Country
Denmark, Finland, Norway

Subject index terms status
Subject indexing assigned by CRD

Subject index terms
Humans; Low Back Pain; Lumbar Vertebrae; Prevalence

Stage of review
Ongoing

Date of registration in PROSPERO
27 April 2015

Date of publication of this revision
27 April 2015

DOI
10.15124/CRD42015017350

Stage of review at time of this submission	Started	Completed
Preliminary searches	No	Yes
Piloting of the study selection process	No	Yes
Formal screening of search results against eligibility criteria	Yes	No
Data extraction	No	No
Risk of bias (quality) assessment	No	No
Data analysis	No	No
