# Supplementary material for: Modic changes—Their associations with low back pain and activity limitation: A systematic literature review and meta-analysis
Source: PLoS One. 2018 Aug 1;13(8):e0200677. doi: 10.1371/journal.pone.0200677 (PMC6070210; doi:10.1371/journal.pone.0200677)
Supplement: S5 Appendix — (DOCX) [file pone.0200677.s005.docx]

**S5 Appendix. PRISMA Checklist**

| **Section/topic** | **#** | **Checklist item** | **Reported on page #** |
| --- | --- | --- | --- |
| **TITLE** | | |  |
| **Title** | **1** | **Identify the report as a systematic review, meta-analysis, or both.** | Title page |
|  |  | Modic Changes: The Association with Low Back Pain and Activity Limitation: a Systematic Literature Review and Meta-Analysis |  |
| **ABSTRACT** | | |  |
| **Structured summary** | **2** | **Provide a structured summary including, as applicable: background; objectives; data sources; study eligibility criteria, participants, and interventions; study appraisal and synthesis methods; results; limitations; conclusions and implications of key findings; systematic review registration number.** | Abstract pages 3-4 |
|  |  | Abstract  Background  Previous systematic reviews have reported positive associations between Modic changes (MC) and low back pain (LBP). Due to the narrow scope of these reviews there is a need for a comprehensive systematic review. The specific objectives were to investigate if MC is associated with non-specific LBP and/or activity limitation and if such an association is modified by other factors.  Methods and Findings  The MEDLINE, CINAHL and EMBASE databases were searched for relevant studies from first record to June 15th 2016. Prospective or retrospective cross-sectional cohort studies and case-control studies including people of all ages from general, working or clinical study populations were eligible for inclusion. Risk of bias assessment and data extraction for associations and potential modifiers or confounders were completed by pairs of independent reviewers. Meta-analysis was performed for homogeneous studies and presented as odds ratios (OR) with 95% CI.  In all, 5210 citations were identified and 31 studies were included. One study had low risk of bias. Fifteen studies (48%) reported statistically significant positive associations between MC and LBP and one study found a statistically significant negative association. Meta-analysis was performed for studies using concordant pain with provocative discography as the clinical outcome resulted in an OR of 4.01 (1.52-10.61). One out of seven studies reported a statistically significant positive association between activity limitation and MC. Lumbar disc level and disc degeneration were found to modify the association between MC and LBP.  Conclusions  The results from this comprehensive systematic review indicate that the associations between MC and LBP related outcomes are inconsistent. The high risk of bias and the heterogeneity in terms of study samples, clinical outcomes and prevalence estimates of MC and LBP may explain these findings. It is likely that new studies with low risk of bias will affect the direction and strength of the associations. |  |
| **INTRODUCTION** | | |  |
| **Rationale** | **3** | **Describe the rationale for the review in the context of what is already known.** | Page 6 |
|  |  | The association between Modic changes (MC) and non-specific LBP has been investigated in three systematic reviews published in 2008 [15, 16] and in 2015 [7]. Both reviews from 2008 concluded that such an association was present for both MC1 and MC2. However, a number of new studies has been published since, and there is a need for an updated review. The review from 2015 found positive associations only for MC1. However, this review included studies published only in English with both symptomatic and asymptomatic people between 15-50 years of age. Moreover, none of the previous reviews included other outcome measures than LBP.  The role of MC in LBP has become even more important because patients with MC following disc herniations have been reported to have favorable outcomes after treatment with antibiotics [17]. This result suggests a bacterial etiology for MC, even though studies investigating this have reported conflicting results [18-21]. The antibiotics study by Albert et al. Created worldwide headlines and much debate among clinicians and researchers due to the potential risk of bias and prospect of treating a large group of patients with long-term high-dose antibiotics [22-28].  The uncertainty about the associations between the different types of MC and LBP, the outdated [15, 16] or narrow scope [7] of the previous systematic reviews and the controversy regarding the clinical implications of MC as a diagnosis for patients with LBP, calls for a comprehensive and updated systematic literature review. |  |
| **Objectives** | **4** | **Provide an explicit statement of questions being addressed with reference to participants, interventions, comparisons, outcomes, and study design (PICOS).** | Page 6 |
|  |  | The objectives of this review were to investigate 1) if the presence of MC (incl. Types and size) in the lumbar region is associated with non-specific LBP and/or activity limitation and 2) if such associations are modified by other factors, e.g. age, sex, and other degenerative MRI findings. |  |
| **METHODS** | | |  |
| **Protocol and registration** | **5** | **Indicate if a review protocol exists, if and where it can be accessed (e.g., Web address), and, if available, provide registration information including registration number.** | Page 7 |
|  |  | A research protocol was developed in advance and registered in the PROSPERO: International prospective register of systematic reviews (http://www.crd.york.ac.uk/PROSPERO/display_record.asp?ID=CRD42015017350). |  |
| **Eligibility criteria** | **6** | **Specify study characteristics (e.g., PICOS, length of follow-up) and report characteristics (e.g., years considered, language, publication status) used as criteria for eligibility, giving rationale.** | Pages 7-8 |
|  |  | 2.2 Criteria for considering studies for this review  2.2.1 Types of studies  Prospective or retrospective cross-sectional cohort studies and case-control studies.  We chose to exclude studies with fewer than 26 individuals. This cutoff was chosen to minimize the risk of having cells in the 2x2 tables that included zero.  2.2.2 Participants  People of all ages from general, working or clinical study populations.  The following exclusion criteria were used:   - Studies including participants diagnosed with specific LBP diagnoses such as: spondylitis, discitis or spondylodiscitis, spondyloarthropathies (e.g. ankylosing spondylitis), fracture (including isthmic spondylolisthesis), spinal cord infarction, malignancy, hematological conditions, juvenile/idiopathic scoliosis. - Participants treated with radiotherapy in the lumbar region. - Participants treated with spinal surgery (although pre-intervention data were eligible for inclusion).   2.2.3 MRI findings and definitions (index test)  MC as seen on MRI of the lumbar spine.  We defined MC as signal changes seen on MRI in the vertebral bone marrow, extending from the endplate. This definition included signal changes regardless of etiology and excluded signal changes only present in the bone marrow away from the endplate.  We chose to include only studies evaluating MC in the whole lumbar spine, disc levels L1-L2 to L5-S1 (except for studies using provocative discography), based on a previous report that the association between MC and LBP is dependent on disc level [29].  2.2.4 Target condition  Non-specific low back pain.  2.2.5 Outcomes (reference standards)   1. Presence and/or intensity of LBP measured by experimental tests (e.g. provocative discography or algometry) or patient-reported. 2. Presence and/or level of activity limitation, measured by the Oswestry Disability Index (ODI), the Roland Morris Disability Questionnaire (RMDQ) or similar tools. |  |
| **Information sources** | **7** | **Describe all information sources (e.g., databases with dates of coverage, contact with study authors to identify additional studies) in the search and date last searched.** | Page 9 |
|  |  | 2.3 Search methods for identification of studies  2.3.1 Electronic searches  A systematic search of the literature was performed using a search strategy developed in collaboration with a research librarian. The three terms “lumbar spine”, “MRI” and “Modic changes” and their relevant synonyms were used as search terms, either as free text or as Medical Subject Headings.  The MEDLINE, CINAHL and EMBASE databases were searched for relevant studies from first record to June 15th 2016. No restrictions were used.  The full electronic search strategy can be found in the S1 Appendix.  2.3.2 Searching other resources  Reference lists of all included studies were examined and all authors were asked to review the list of included studies for omissions. |  |
| **Search** | **8** | **Present full electronic search strategy for at least one database, including any limits used, such that it could be repeated.** | Appendix S1 |
|  |  | **MEDLINE**  Search terms within groups were combined with the boolean operator OR.   \| **Group** \| **Search term** \| \| --- \| --- \| \| lumbar spine \| Lumbar Vertebra/ \| \|  \| Lumbosacral Region/ \| \|  \| lumb*.tw,kw. \| \|  \| low back.tw,kw. \| \| **AND** \| \| \| MRI \| Magnetic Resonance Imaging/ \| \|  \| magnetic resonance.tw,kw. \| \|  \| mri.tw,kw. \| \|  \| nmr.tw,kw. \| \|  \| diagnostic imaging.tw,kw. \| \| **AND** \| \| \| Modic \| Intervertebral Disc Degeneration/ \| \|  \| Spondylosis/ \| \|  \| Bone Marrow/ \| \|  \| spondylosis.tw,kw. \| \|  \| bone marrow.tw,kw. \| \|  \| osteochondrosis.tw,kw. \| \|  \| osteochondritis.tw,kw. \| \|  \| modic.tw,kw. \| \|  \| apophysis.tw,kw. \| \|  \| scheuermann.tw,kw. \| \|  \| schmorl*.tw,kw. \| \|  \| discovertebral.tw,kw. \| \|  \| (edema OR oedema).tw,kw. \| \|  \| ((endplate? or end plate? or end-plate?) not "motor endplate?").tw,kw. \|   Database: Ovid ”MEDLINE(R) In-Process & Other Non-Indexed Citations and Ovid MEDLINE(R) 1946 to Present” |  |
| **Study selection** | **9** | **State the process for selecting studies (i.e., screening, eligibility, included in systematic review, and, if applicable, included in the meta-analysis).** | Pages 9-10 |
|  |  | 2.4.1 Selection of studies  Two reviewers (CH, TSJ) independently screened the titles and abstracts to exclude clearly irrelevant papers. For each potentially eligible study, the full article was retrieved and independently assessed for inclusion (CH, TSJ). Any discrepancies were resolved by consensus. Where multiple publications used data from the same study sample, we chose the article with the most complete data related to the association between MC and LBP and/or activity limitation.  In cases where association data were not presented in a format that we could use for data extraction, we contacted the authors to request additional data, as recommended by the Cochrane Handbook [30].  We assessed the eligibility of non-English papers using Google Translate and, when this was impossible due to incomplete Optical Character Recognition obtained from scans of paper copies, with the help of a native speaker of the language in question. |  |
| **Data collection process** | **10** | **Describe method of data extraction from reports (e.g., piloted forms, independently, in duplicate) and any processes for obtaining and confirming data from investigators.** | Page 10 |
|  |  | Data extraction and risk of bias assessment was completed by independent reviewers (CH, PK, AE, JStS, CLY, JK, JN, JSoS, KS, TSJ), allocated in pairs (except for non-English papers, where a single assessor was used), using spreadsheets (S2 Appendix, Tab 1-3). All reviewers were pre-trained through pilot testing of the process. Inconsistencies were resolved by consensus or, if needed, by including a third reviewer (CH or TSJ).  Data regarding sample source, number of subjects, age, MRI parameters, observers, MC (including types and size), clinical outcomes, and strength of association between LBP and/or activity limitation and MC were extracted from the papers (S2 Appendix). |  |
| **Data items** | **11** | **List and define all variables for which data were sought (e.g., PICOS, funding sources) and any assumptions and simplifications made.** | Pages 10-11 |
|  |  | Data regarding possible modifiers or confounders of the association between MC and clinical outcomes were extracted and classified according to how the co-variates were analysed; a) by matching on the covariate(s), b) by restricting participant selection so that all groups had the same covariate value or c) by adjustment for covariates in the statistical analysis. For the purpose of this review, only analyses investigating single covariates were included. The reason for this was that adjustment by groups of covariates (e.g. age, sex, etc.) may change the estimate of the association, but will not provide information as to which of the group co-variates or combinations of co-variates are modifying the association. Because of the exploratory nature of this part of the review, we chose not to make a list of pre-defined candidate variables [31]. |  |
| **Risk of bias in individual studies** | **12** | **Describe methods used for assessing risk of bias of individual studies (including specification of whether this was done at the study or outcome level), and how this information is to be used in any data synthesis.** | Page 11 |
|  |  | 2.4.3 Risk of bias assessment  We based our risk of bias assessment on the QUADAS 2 tool [32]. This tool is used to evaluate the following four key domains: *study sample*, *index test*, *reference standard(s),* *timing and data analysis* based on signaling questions and questions regarding applicability. For each domain, studies were classified as having “low risk of bias”, ”high risk of bias” or “unclear” based on a number of signaling questions. Studies were classified as having an “overall low risk of bias” if all four domains were scored as “low risk of bias” [32]. We added additional signaling questions pertaining to items we found particularly important for the subject of this review. After pilot testing, we modified some of the risk of bias questions and response options, making them more intuitive to answer (S2 Appendix). The questions regarding applicability were not used in this study. The result of our risk of bias assessment was not used as an inclusion criterion. |  |
| **Summary measures** | **13** | **State the principal summary measures (e.g., risk ratio, difference in means).** | Pages 11-12 |
|  |  | 2.4.4 Statistical analysis and data synthesis  Raw data for 2x2 tables or group differences were extracted where possible to calculate odds ratio (OR) with 95% confidence interval (CI) for dichotomous outcomes or to perform t-tests for continuous outcomes. In cases where results were presented in the form of ORs or mean differences, without raw data, we present them as stated in the article, using data from the crude analysis, i.e. unadjusted. Data supplied by authors on request were treated in the same manner. ORs and 95% CIs were calculated for 2x2 tables. For tables containing 0 in one of the cells, we added 0.5 to all cells [33]. Differences in means between groups were analysed using t-test. Statistical analyses were performed using STATA (version 12.1, StataCorp, College Station, Texas, USA).  Associations between subtypes and sizes of MC and outcomes were determined with reference to participants with no MC. |  |
| **Synthesis of results** | **14** | **Describe the methods of handling data and combining results of studies, if done, including measures of consistency (e.g., I^2^) for each meta-analysis.** | Page 12 |
|  |  | Results were pooled where it was deemed possible and appropriate (e.g. homogeneous in terms of study sample or outcome), and associations reported as ORs and 95% CI. Due to the heterogeneity in terms of the prevalence estimates of MC and study sampling, a random effect model was used. I^2^ statistics were used to quantify inconsistency across studies. *Comprehensive Meta-Analysis* (version 3, Biostat, Englewood, USA) was used for meta-analysis. |  |

Page 1 of 2

| Section/topic | # | Checklist item | **Reported on page #** |
| --- | --- | --- | --- |
| **Risk of bias across studies** | **15** | **Specify any assessment of risk of bias that may affect the cumulative evidence (e.g., publication bias, selective reporting within studies).** | Page 11 |
|  |  | 2.4.3 Risk of bias assessment  We based our risk of bias assessment on the QUADAS 2 tool [32]. This tool is used to evaluate the following four key domains: *study sample*, *index test*, *reference standard(s),* *timing and data analysis* based on signaling questions and questions regarding applicability. For each domain, studies were classified as having “low risk of bias”, ”high risk of bias” or “unclear” based on a number of signaling questions. Studies were classified as having an “overall low risk of bias” if all four domains were scored as “low risk of bias” [32]. We added additional signaling questions pertaining to items we found particularly important for the subject of this review. After pilot testing, we modified some of the risk of bias questions and response options, making them more intuitive to answer (S2 Appendix). The questions regarding applicability were not used in this study. The result of our risk of bias assessment was not used as an inclusion criterion. |  |
| **Additional analyses** | **16** | **Describe methods of additional analyses (e.g., sensitivity or subgroup analyses, meta-regression), if done, indicating which were pre-specified.** |  |
|  |  | Pre-determined sensitivity analyses were performed for publication bias and overall risk of bias. The classification of association (positive, negative or contradictory) was then tested against mean age, year of publication, number of participants, and overall risk of bias using Fischer’s exact test. |  |
| **RESULTS** | | |  |
| **Study selection** | **17** | **Give numbers of studies screened, assessed for eligibility, and included in the review, with reasons for exclusions at each stage, ideally with a flow diagram.** | Page 13 |
|  |  | 3.1 Selection of studies  In all, 5210 citations were identified from the three databases, yielding 3834 records after duplicates had been removed. After review of title and abstract, 3377 records were excluded, resulting in a total of 457 papers eligible for full text assessment. Four hundred and twenty studies were excluded in the full text assessment (S3 Appendix), resulting in 37 potential candidates. Two additional studies were found through manual search [34] and by a person from the research team [35] respectively, resulting in 39 potentially acceptable studies.  In eleven of the 39 studies we requested additional data from the authors needed for analysis [34, 36-45], but received it only from three [37, 40, 41]. We thus ended up including 31 studies (See flow diagram, Fig. 1). |  |
| **Study characteristics** | **18** | **For each study, present characteristics for which data were extracted (e.g., study size, PICOS, follow-up period) and provide the citations.** | Table 1 |
|  |  | Please see Table 1 |  |
| **Risk of bias within studies** | **19** | **Present data on risk of bias of each study and, if available, any outcome level assessment (see item 12).** | Page 15 |
|  |  | 3.3 Risk of bias assessment  The results from the risk of bias assessment can be seen in Table 2. Overall, only one study [47] was classified as having overall low risk of bias, i.e. with low risk of bias in all four key domains. Five studies had three domains with low risk of bias, three studies had two domains with low risk of bias, fourteen studies had one domain with low risk of bias and eight studies were classified as having no domains with low risk of bias. |  |
| **Results of individual studies** | **20** | **For all outcomes considered (benefits or harms), present, for each study: (a) simple summary data for each intervention group (b) effect estimates and confidence intervals, ideally with a forest plot.** | Page 15-18 |
|  |  | 3.4 Association between MC and LBP  3.4.1 Association between types of MC and LBP  Across all included papers, 30 of 31 studies reported on the association between MC (regardless of type) and LBP. Of these 30, 15 found statistically significant positive associations with ORs ranging from 1.53 (95% CI 1.02–2.29) to 83.10 (95% CI 4.85-1424.05), while only one found a statistically significant negative association with a mean difference between patients with and without MC2 of -3.2 (-5.39 – -1.01) on a ”back pain score” ranging from 0 to 30 [37]. The remaining fourteen studies reported statistically non-significant findings, of which eight [37, 40, 41, 48-52] reported negative (but non-significant) estimates on at least one of their outcome measures. No studies reported contradictory statistically significant associations (Table 3).  TABLE3  Across all included articles, thirteen studies reported on the association between MC1 and LBP. Six of these found statistically significant positive associations. Five reported ORs ranging from 2.06 (95% CI 1.12–3.79) to 51.67 (95% CI 11.43-233.51) for dichotomous outcomes [46, 53-56] whereas one study reported statistically significant positive associations using continuous outcomes [29] (Table 3). The remaining seven studies reported statistically non-significant findings for associations regarding MC1 [37, 40, 52, 57-60].  Ten studies reported on the association between MC2 and LBP. Four reported statistically significant positive associations with ORs ranging from 1.53 (95% CI 1.02–2.29) to 15.46 (95% CI 1.89-126.67) [53, 54, 56, 58]. One study reported a statistically significant negative association with a mean difference between patients with and without MC2 of -3.2 (-5.39 – -1.01) [37]. The remaining five studies reported statistically non-significant findings for associations regarding MC2 [29, 55, 57, 59, 60].  The wide range of ORs and the broad and overlapping 95% CIs indicate that there is no significant difference between MC1 and MC2 in regards to their association with LBP (Table 3).  Two studies reported on the association between MC3 and LBP and one of these found a positive association with OR 2.51 (95% CI 1.05-5.97) [55] (Table 3). The remaining study reported a statistically non-significant finding for association regarding MC3 [58].  3.4.2 Association between different sizes of MC and LBP  Three studies [29, 53, 56] reported statistically significant positive associations between extensive MC and LBP; two reported ORs of 1.83 (95% CI 1.14–2.94) and 83.10 (95% CI 4.85-1424.05) and one reported on continuous outcomes, see Table 3.  However, the estimates for extensive MC were not different from those for MC of any type, regardless of size.  3.4.4 LBP intensity in patients with and without MC  None of the six clinical studies investigating the difference in LBP intensity between patients with and without MC found a significant difference between the two groups [37, 41, 47, 49, 52, 57].  3.5 Association between MC and activity limitation  3.5.1 Association between MC and activity limitation  One of seven studies (three clinical, three non-clinical and one case-control) reporting on activity limitation outcomes found a statistically significant association MC and activity limitation. Määttä et al. reported an association between activity limitation (ODI>15%) and both any MC and MC2, OR 1.47 (95% CI 1.04-2.10) and 1.56 (95% CI 1.06-2.31), respectively, but not for MC1 [53]. (Table 5)  TABLE5  3.5.2 Level of activity limitation in patients with and without MC  None of the four clinical studies investigating the difference in activity limitation levels between patients with and without MC found a significant difference between the two groups [41, 49, 52, 60]. |  |
| **Synthesis of results** | **21** | **Present results of each meta-analysis done, including confidence intervals and measures of consistency.** | Page 17 |
|  |  | 3.4.3 Pooled results  Due to the heterogeneity of the study samples, pertaining to outcome measures and study sampling, no meta-analysis was performed for these studies. Meta-analysis was however performed for the nine studies using concordant pain with provocative discography as outcome measure. Separate analyses were made for MC any, MC type 1 and MC type 2 resulting in ORs (95% CI) of 4.01 (1.52-10.61), 6.14 (2.47-15.27), and 3.15 (1.00-9.93), respectively, indicating that there was no significant difference in the association with LBP between the two types of MC. Substantial heterogeneity was identified for all three analyses with I2-values of 84, 64 and 81, respectively (Table 4). |  |
| **Risk of bias across studies** | **22** | **Present results of any assessment of risk of bias across studies (see Item 15).** | Page 15 |
|  |  | 3.3 Risk of bias assessment  The results from the risk of bias assessment can be seen in Table 2. Overall, only one study [47] was classified as having overall low risk of bias, i.e. with low risk of bias in all four key domains. Five studies had three domains with low risk of bias, three studies had two domains with low risk of bias, fourteen studies had one domain with low risk of bias and eight studies were classified as having no domains with low risk of bias.  TABLE2 |  |
| **Additional analysis** | **23** | **Give results of additional analyses, if done (e.g., sensitivity or subgroup analyses, meta-regression [see Item 16]).** | Pages 19-20 |
|  |  | 3.8 Sensitivity analysis  Of the 30 studies investigating LBP (one study did not), 15 studies reported statistically significant positive associations with MC. The results of the sensitivity analysis is reported in Table 6.  TABLE6  The publication of statistically significant positive associations between MC and LBP was not related to year of publication (p<0.79), classified as 1998-2004 (n=5), 2005-2010 (n=10) and 2011-2016 (n=15), or to the total number of participants (p<0.14), divided into <100 participants (n=12), 100-500 participants (n=11) and more than 500 participants (n=7).  As only 1 of 7 studies evaluating activity limitation was classified as having statistically significant positive associations and the remaining studies showed insignificant associations, sensitivity analysis for this outcome was not meaningful.  Only one study was classified as having “no overall risk of bias” and performing sensitivity analysis in relation to the overall risk of bias assessment was therefore not meaningful.  3.8.1 Post hoc sensitivity analysis  To further investigate the possible influence of bias and other factors in the reporting of statistically significant associations between MC and LBP, we performed post hoc analyses of the classification of association (statistically positive association, yes/no) in relation to individual risk of bias domains and signaling questions, LBP outcomes, study design, and MRI field strength.  There was a statistically significant difference (p<0.01) in the distribution of studies using continuous or dichotomous outcomes (Table 6). All 15 studies reporting significant positive associations had used dichotomous outcomes, e.g. “LBP < 6 weeks”, as compared to only half (53%) of the 15 studies not reporting significant positive association. No other statistically significant differences were identified between the two groups of studies. |  |
| **DISCUSSION** | | |  |
| **Summary of evidence** | **24** | **Summarize the main findings including the strength of evidence for each main outcome; consider their relevance to key groups (e.g., healthcare providers, users, and policy makers).** | Pages 20 + 26-27 |
|  |  | 4.1 Main findings  In summary, the results from this review showed inconsistent associations between MC and both LBP and activity limitation. Only half of the studies reported statistically significant positive associations between MC and LBP. Both pooled and individual study data indicate that there is no difference in the strength of association of MC1 and MC2 with LBP. Among patients with LBP, the intensity of LBP does not seem to differ between those with MC and those without. Only one of eight studies found an association between MC and activity limitation. Finally, our results indicate that disc level and disc degeneration modify the association between MC and LBP.  4.5 Recommendations for further research  The widely different prevalence rates reported for MC in not so different populations may indicate inconsistent phenotyping of MC. Agreed characterization of MC across studies is needed, including criteria for size and for differentiation from other signal changes (e.g., fat or oedema in osteophytes, inhomogeneous bone marrow, hemangiomas abutting the endplate), or at least a concise reporting of the methods used to evaluate these findings, in order to be able to compare results between studies.  In light of the results of our risk of bias assessment, we urge researchers to improve their reporting of the methods used. We particularly found weaknesses related to the selection of study samples, reliability testing on MRI assessments, blinding and study logistics (timing of assessments). Researchers could also assess whether other MC characteristics (e.g. location [53], extent [53], their signal after fat suppression [78]) may be more relevant to pain than MC type based on conventional T1- and T2-weighted MRI.  To be able to further our understanding of the details in the association between MC and LBP, we need large population based cohort studies with low risk of bias that allow for stratified or multivariable analyses including known and suspected modifiers.  4.6 Clinical implications of our findings  The lack of difference in pain intensity between patients with MC and patients without MC, along with the sparse knowledge around other distinguishing clinical characteristics, makes identification of patients with MC difficult without the use of MRI.  However, this may be without clinical relevance, as our finding of a more inconsistent association between LBP and MC than previously shown should call for caution when using “Modic changes” as a diagnosis, explanation for LBP, and indication for specific treatment in patients with non-specific LBP. |  |
| **Limitations** | **25** | **Discuss limitations at study and outcome level (e.g., risk of bias), and at review-level (e.g., incomplete retrieval of identified research, reporting bias).** | Pages 24-25 |
|  |  | 4.2.6. Overall risk of bias of included studies  There was an overall risk of bias in all included studies but one [47]. This risk was partly due to insufficient reporting and may not necessarily imply actual bias. Still, risk of bias needs to be taken into account when interpreting the present results and when performing new primary studies.  The most common problems within each of the four bias domains were; 1) Lack of randomly or consecutively selected study participants, which could introduce a risk of selection bias. 2) Lack of reliability testing raises concerns about misclassification and influences the reporting of prevalence rates of MC, and thus also the strengths, directions and validity of associations since these are dependent on the prevalence. 3) Lack of blinding between assessment of outcome measure and MRI results. This was mainly an issue for discography studies where patients were referred for the procedure on the basis of the results of their MRI scan, which may have introduced beliefs that could affect their reporting of pain, and 4) Failure to report on the timing of the MRI and clinical outcome assessments. The longer the time period between the two, the bigger the risk of change in either one.  4.4.1 Limitations  In this review we also included case-control studies, although they are less suited for our purpose due to the fact that the groups are from different samples, thus introducing a potential bias, as described in the Cochrane Handbook [77]. However, our sensitivity analysis did not show that study design influenced the results.  By only including studies that had evaluated MC at all lumbar levels, it is possible that we excluded high quality studies that could have informed on the association between MCs and LBP. However, the decision was made to be able to investigate the modifying effect of disc level on the association between MC and LBP since this has not been done before. |  |
| **Conclusions** | **26** | **Provide a general interpretation of the results in the context of other evidence, and implications for future research.** | Page 27 |
|  |  | 4.7 Conclusion  The results from this comprehensive systematic review show that the associations between MC and both outcomes of LBP and activity limitation are inconsistent. Also, no difference in LBP intensity or level of activity limitation was found between patients with and without MC. These results questions the conclusions from previously published reviews that MC findings may constitute a specific clinically relevant subgroup among people with LBP. Disc level and disc degeneration were identified as potential modifying factors. The heterogeneity in terms of study samples, classification of MC, clinical outcomes and prevalences of MC and LBP may explain the inconsistent associations. New studies with low risk of bias are likely to affect the direction and strength of the associations. |  |
| **FUNDING** | | |  |
| **Funding** | **27** | **Describe sources of funding for the systematic review and other support (e.g., supply of data); role of funders for the systematic review.** |  |
|  |  | The study was funded by a grant from the research foundation “ELIB – et liv I bevegelse”, (<http://elibforskning.no/forskningsprojekt/modic-changes-prevalence-and-association-with-low-back-pain-a-systematic-literature-review-and-meta-analysis/#more-375>).  TSJ: Salary funded by grants from the Foundation for Chiropractic Research and Post Graduate Education.  The funders did not influence any aspect of study design, conduct, analysis, interpretation, conclusions or preparation of manuscript. |  |

*From:*  Moher D, Liberati A, Tetzlaff J, Altman DG, The PRISMA Group (2009). Preferred Reporting Items for Systematic Reviews and Meta-Analyses: The PRISMA Statement. PLoS Med 6(6): e1000097. doi:10.1371/journal.pmed1000097

For more information, visit: **www.prisma-statement.org**.

Page 2 of 2
